# High serum C-X-C motif chemokine ligand 10 (CXCL10) levels may be associated with new onset interstitial lung disease in patients with systemic sclerosis: evidence from observational, clinical, transcriptomic and *in vitro* studies

**DOI:** 10.1016/j.ebiom.2023.104883

**Published:** 2023-11-22

**Authors:** Yehya Al-Adwi, Isabella Maria Atzeni, Berber Doornbos-van der Meer, Marcel John van der Leij, Rita Delphine Maiko Varkevisser, Bart-Jan Kroesen, Alja Stel, Wim Timens, Christiaan Tji Gan, Harry van Goor, Johanna Westra, Douwe Johannes Mulder

**Affiliations:** a University of Groningen, University Medical Centre Groningen, Department of Internal Medicine, Division of Vascular Medicine, Groningen, the Netherlands; b University of Groningen, University Medical Centre Groningen, Department of Rheumatology and Clinical Immunology, Groningen, the Netherlands; c University of Groningen, University Medical Centre Groningen, Department of Laboratory Medicine, Groningen, the Netherlands; d Department of Endocrinology, University Medical Centre Groningen, Groningen, the Netherlands; e University of Groningen, University Medical Centre Groningen, Department of Pathology and Medical Biology, Groningen, the Netherlands; f University of Groningen, University Medical Centre Groningen, Department of Pulmonary Diseases and Tuberculosis, Groningen, the Netherlands

**Keywords:** CXCL10, Biomarker, Inflammation, Fibrosis, ILD, Systemic sclerosis

## Abstract

**Background:**

Systemic sclerosis-interstitial lung disease (SSc-ILD) is the leading cause of death in patients with SSc. There is an unmet need for predictive biomarkers to identify patients with SSc at risk of ILD. Previous studies have shown that interferon (IFN) pathways may play a role in SSc. We assessed the use of C-X-C motif chemokine ligand 10 (CXCL10) as a predictive biomarker for new onset of ILD in patients with SSc.

**Methods:**

One-hundred-sixty-five (Female, N = 130) patients with SSc (SSc-ILD, N = 41) and 13 (Female, N = 8) healthy controls were investigated retrospectively. CXCL10 protein levels were measured by ELISA. We performed log rank analysis with baseline CXCL10 serum levels. CXCL10 nanoString data from lung tissues obtained from transplanted patients with SSc-ILD were extracted. Fifteen (Female, N = 10) patients with SSc (SSc-ILD, N = 7) were recruited for bronchoalveolar lavage (BAL) procedure. Lung fibroblasts were treated with BAL-fluid or serum from patients with SSc with or without ILD. Inflammatory/fibrotic genes were assessed.

**Findings:**

Serum CXCL10 levels were higher in patients with SSc-ILD compared to SSc patients without ILD [Median (IQR):126 pg/ml (66–282.5) vs. 78.5 pg/ml (50–122), *P* = 0.029, 95% CI: 1.5 × 10^−6^ to 0.4284]. Survival analysis showed that baseline CXCL10 levels >78.5 pg/ml have a 2.74-fold increased risk of developing new onset of ILD (Log-rank: *P* = 0.119) on follow-up. CXCL10 levels in BAL supernatant were not different in patients with SSc-ILD compared to SSc without ILD [76.1 pg/ml (7.2–120.8) vs. 22.3 pg/ml (12.1–43.7), *P* = 0.24, 95% CI: −19.5 to 100]. NanoString showed that CXCL10 mRNA expression was higher in inflammatory compared to fibrotic lung tissues [4.7 (4.2–5.6) vs. 4.3 (3.6–4.7), *P* = 0.029]. Fibroblasts treated with SSc-ILD serum or BAL fluids overexpressed CXCL10.

**Interpretations:**

Clinical, transcriptomic, and *in vitro* data showed that CXCL10 is potentially involved in early SSc-ILD. More research is needed to confirm whether CXCL10 can be classified as a prospective biomarker to detect patients with SSc at higher risk of developing new onset ILD.

**Funding:**

This collaborative project is co-financed by the 10.13039/501100016238Ministry of Economic Affairs and Climate Policy of the Netherlands utilizing the PPP-allowance made available by the 10.13039/100016036Top Sector Life Sciences & Health to stimulate public-private partnerships (PPP-2019_007). Part of this study is financially supported by 10.13039/100013995Sanofi Genzyme (NL8921).

**Research in context**

**Evidence before this study**

SSc is an inflammatory-fibrotic disease of the skin and internal organs where most patients develop inflammatory cues early in the disease course. Previous studies have shown that IFN pathways may govern early inflammatory SSc and SSc-ILD disease. Moreover, a major proinflammatory IFN-γ chemokine, CXCL10 was reported to predict early SSc events when high in serum of patients with SSc.

**Added value of this study**

We used clinical (systemic and local), transcriptomic, and *in vitro* data to demonstrate that CXCL10 is a critical player in early progression of SSc-ILD pathogenesis. We also report the possibility that IL-6 can induce CXCL10 expression, next to IFN-γ, at least in lung fibroblasts. This further supports the use of IL-6R blocker (Tocilizumab) to treat inflammatory SSc-ILD patients.

**Implications of all the available evidence**

ILD is associated with around 1/3 of SSc deaths. There is unmet need to find biomarkers that can predict new onset of ILD in patients with SSc which may allow early treatment. Our work shows a potential role for CXCL10 to predict new onset of ILD in patients with SSc. Future studies should investigate cut-off values for serum CXCL10 in prospective cohorts. This will enable the utilization of CXCL10 as a biomarker to precisely predict new onset of SSc-ILD.

## Introduction

Systemic sclerosis (SSc) is a fibroproliferative systemic auto-immune disease. Particularly in the early stages of the disease, inflammatory changes are present in the skin and internal organs.^1^ The high mortality rate of SSc arises from its lung complications (SSc-associated interstitial lung disease, SSc-ILD) which is responsible for a third of SSc deaths.^2^^,^^3^ Until now, several studies have aimed to find reliable biomarkers for SSc-ILD prediction and progression but without success^3^, ^4^, ^5^

Interferons (IFN) are key regulators of the innate and adaptive immune system. They induce a cascade of reactions in response to type-I T helper cells (Th1) cytokines [Interleukin (IL)-2, IL-12, IL15, IL-18 and IL-23], or to bacterial or viral stimuli.^6^ These reaction cascades involve various cytokines and chemokines that are crucial for the trafficking and maturation of effector immune cells and mesenchymal progenitors.^7^^,^^8^ In rheumatic autoimmune diseases, chemokine signalling pathways are dysregulated at various levels. Overexpression and production of IFN-induced chemokines have been reported in several autoimmune diseases including SSc.^9^, ^10^, ^11^

C-X-C motif chemokine ligand 10 (CXCL10), also known as Interferon-γ-induced protein 10 (IP-10), is a major pro-inflammatory Th1 chemokine that is involved in the pathophysiology of multiple autoimmune diseases.^12^, ^13^, ^14^, ^15^ It is released in response to IFN-γ from CD4+, CD8+, and natural killer cells. CXCL10 functions through the binding to C-X-C chemokine receptor 3 (CXCR3) and attracts inflammatory cells including monocytes, Th1, CD8+ T, NK, NKT, and dendritic cells.^15^, ^16^, ^17^ When the CXCL10/CXCR3 axis is activated, a positive feedback loop of Th1 cell recruitment is initiated perpetuating the inflammatory reaction resulting in more IFN- γ release.^18^ It has been reported that patients with the diffuse cutaneous subtype of SSc have higher serum levels of CXCL10 compared to healthy controls.^19^ In a longitudinal study, newly diagnosed patients with SSc had higher levels of CXCL10, which declined on follow-up, suggesting the involvement of CXCL10 early in the disease process.^20^

It is unclear whether CXCL10 plays a role in initiating ILD in patients with SSc. Additionally, since SSc is a systemic disease, it remains a question whether systemic CXCL10 levels mirror local levels in the lungs. Although Smith et al. suggested that CXCL10 levels are specifically higher in patients with SSc-ILD compared to SSc patients without lung complications,^21^ little is known about whether CXCL10 levels systemically (blood) and locally (lungs) correlate and may predict long-term ILD development and/or worsening of lung function. We hypothesize that CXCL10 plays a major role in early SSc-ILD development and could serve as a biomarker for the prediction of ILD. We investigated CXCL10 gene and protein expression in the systemic and local compartments and associated these findings with clinical parameters. We also performed *in vitro* studies to confirm our clinical observations and propose an alternative (inflammatory) pathway through the IL-6 axis for CXCL10 induction using the fibrosis-effector cells, the lung fibroblasts.

## Methods

### Study design

**Fig. 1:**
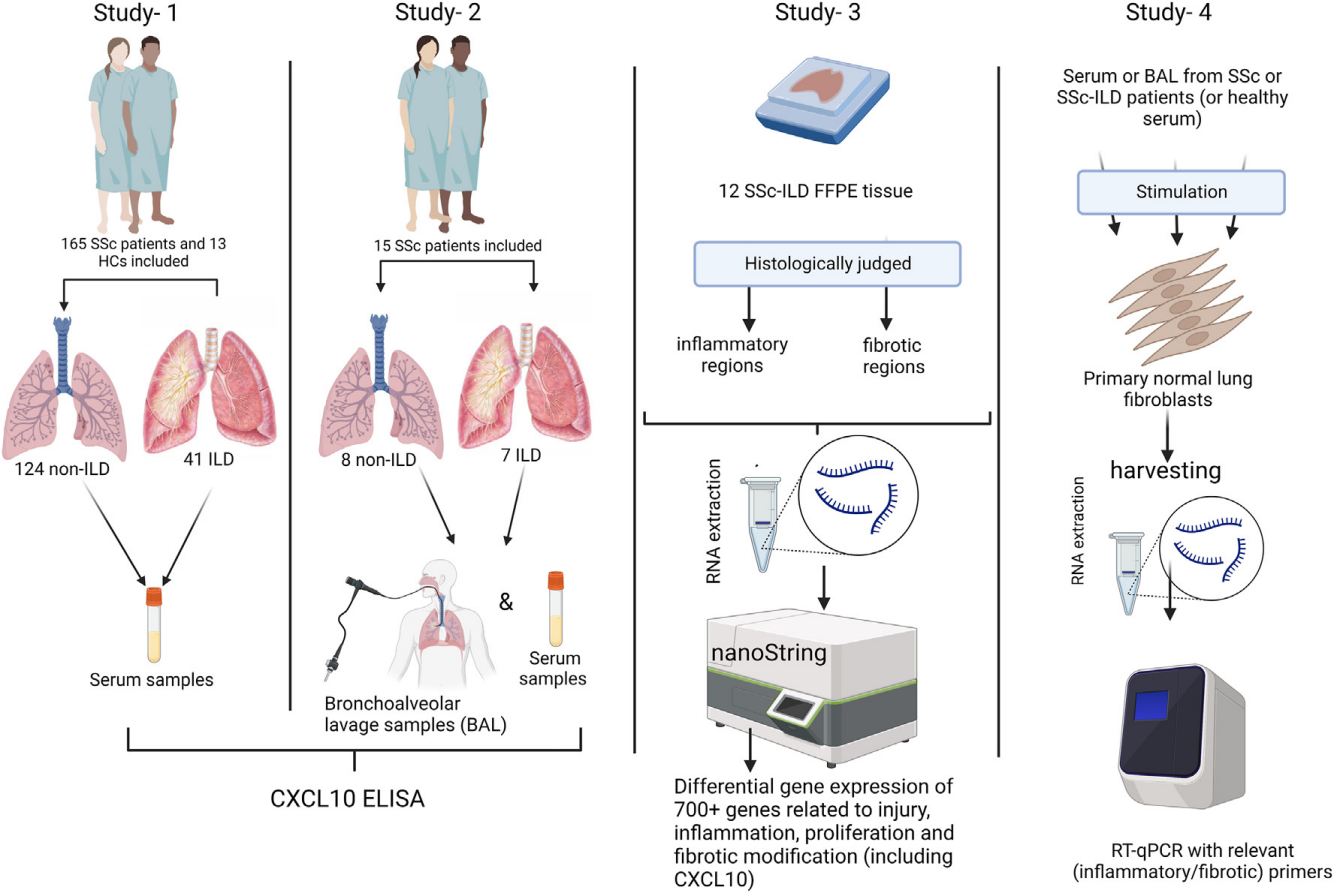
Schematic overview of the performed studies. (Study-1) is a retrospective cohort that has followed 165 patients with SSc and 13 healthy controls. (Study-2) is a cross-sectional study that recruited 15 patients with SSc for bronchoalveolar lavage. (Study-3) is a transcriptomic study on SSc-ILD lung tissue sections. (Study-4) is an *in vitro* study where lung fibroblasts were stimulated with local or systemic SSc/SSc-ILD fluids. Created by Biorender.com.

Four separate interconnecting studies including a retrospective clinical cohort, a cross-sectional clinical study, a transcriptomic study, and finally an *in vitro* study have been performed in a single research center (University Medical Center Groningen). These are discussed in detail below. Please refer to the outline in Fig. 1.

### Study- 1

#### Patients and controls

In this retrospective study, 165 patients with SSc who visited the outpatient clinic in the University Medical Centre Groningen between January 2013 and December 2020 were included for analysis. This retrospective cohort has previously been described.^22^ In short, patients between 18 and 70 years of age who fulfilled the American College of Rheumatology/European League Against Rheumatism 2013 (ACR/EULAR 2013) classification criteria for a clinical diagnosis of SSc, were included (Flowchart 1).^23^ Serum blood samples were collected as part of routine outpatient visits and stored at −20 °C. Pulmonary function tests (PFTs) were performed according to ATS/ERS guidelines as standard of care. The performed PFTs consisted of diffusing capacity for carbon dioxide (DL_CO_) and forced vital capacity (FVC). Abnormal DL_CO_ was defined as <80% of the predicted value and FVC was considered abnormal when <70% of the predicted value. ILD was diagnosed based on high-resolution computed tomography (HRCT) during this follow-up period. Time until diagnosis was defined as the difference between date of diagnosis and first outpatient visit. Thirteen age- and sex-matched healthy controls were enrolled for blood samples. The sex of participants was self-reported.

**Flowchart 1:**
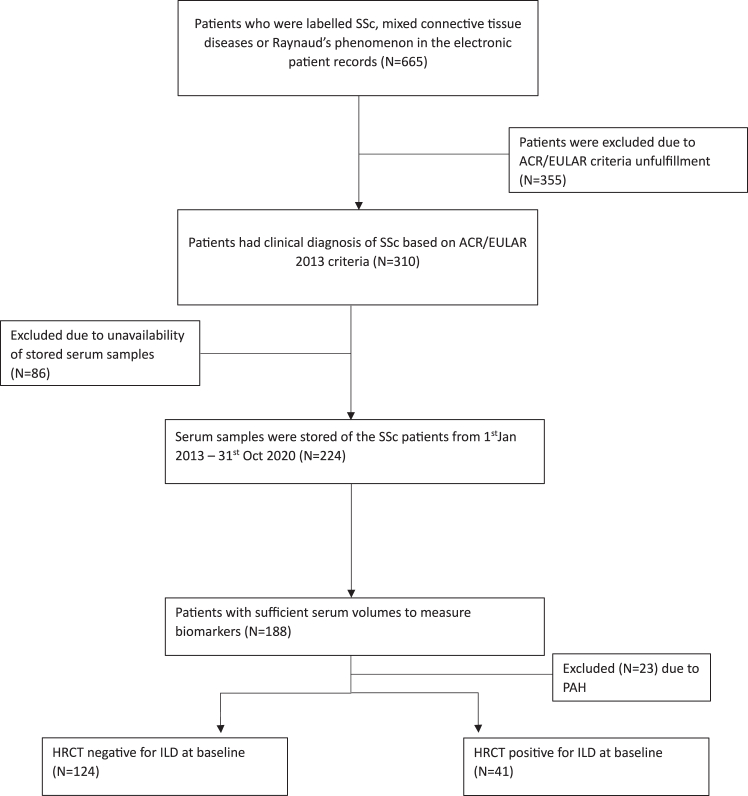
Inclusion and exclusion criteria for study-1.

### Study- 2

To investigate local levels of CXCL10, we performed a cross-sectional study recruiting SSc-treatment naïve patients visiting the Rheumatology outpatient clinic of the University Medical Centre Groningen from January 2021 until November 2021 for a bronchoalveolar lavage procedure (BAL). Of the 160 eligible patients, 15 underwent the BAL procedure, of which 7 patients had ILD (Flowchart 2). Sex of study subjects was self-reported. Clinical characteristics can be found in Supplementary Table S1.

**Flowchart 2:**
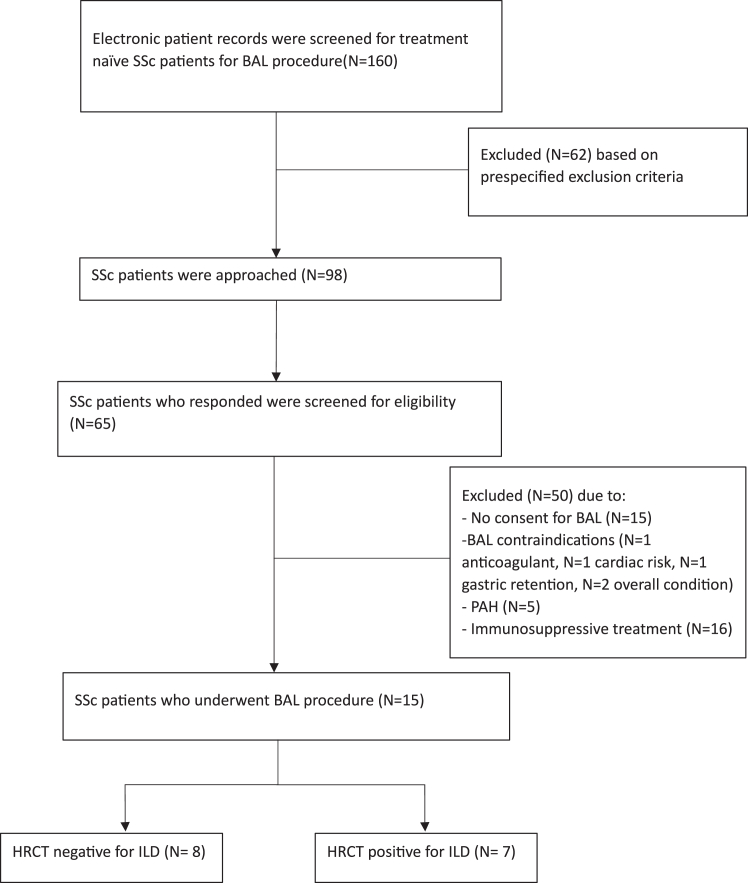
Inclusion and exclusion criteria for study-2.

#### Bronchoalveolar lavage (BAL) procedure

Briefly, after sedation, 150 ml of normal saline (0.9% NaCl) was instilled through a fiber-optic bronchoscope wedged in subsegmental bronchi in the middle of one of the right lower lobes. Next, the fluid was aspirated; typically, the recovered lavage volume was around 100–120 ml. Thereafter, the lavage fluid was filtered to remove debris and mucus and BAL cells were obtained through centrifugation. BAL supernatant was aliquoted and stored at −20 °C.

#### CXCL10 Enzyme-linked immunosorbent assay (ELISA) on serum and BAL supernatant

Three mL of BAL supernatant was concentrated (6x) using Pierce Protein Concentrator (Pierce Technologies, IL, USA) with a molecular weight cut-off value of 10 kDa to retain the CXCL10 protein (8.7 kDa). Local CXCL10 protein levels were assessed by ELISA (R&D systems, DY266, MN, USA) according to manufacturer's instructions and as described previously.^24^^,^^25^ High-performance ELISA buffer (product no.: M1940, Sanquin, Amsterdam, The Netherlands) was used during serum incubation to prevent non-specific reactions. All samples were assayed in duplicates (2x and 8x dilutions). Absorbance was read at 450 nm using SpectraMax plus 384 Microplate-Reader spectrophotometer (Molecular devices, San Jose, CA).

### Study- 3

#### NanoString nCounter human fibrosis panel in formalin-fixed paraffin-embedded (FFPE) lung sections

This transcriptomic analysis was previously performed for an exploratory project (unpublished). Twelve FFPE sections excised from 10 SSc-ILD lung transplants were used as the source of RNA for the nCounter analysis. The twelve SSc-ILD sections were examined under a light microscope by two independent scientists to determine inflammatory and fibrotic regions and were mapped on glass slides. Inflammatory regions were defined by slight non-collagenous thickening of the alveolar walls with variable numbers of mononuclear inflammatory cells, so-called non-specific interstitial pneumonia (NSIP)-like pattern while the fibrotic regions were defined by complete destruction of normal parenchymal architecture and accumulation of collagens where alveolar walls are extremely thickened with areas of diffuse fibrosis with usual interstitial pneumonia (UIP) and fibrotic NSIP patterns. Thereafter, each region of interest was scrapped off the glass slide using a scalpel and Qiagen RNeasy buffer, then placed in a test tube. Then, RNA was isolated using the RNeasy FFPE kit (QIAGEN GmbH, Hilden, Germany) according to manufacturer instructions. After isolation, RNA integrity and quantity were determined using Qubit 4 fluorometer (Life Technologies, Singapore). Next, RNA concentrations were adjusted to 100 ng/10 μL and a hybridization reaction with the capture and reporter probes was performed using a conventional thermocycler. The panel (cat no.: XT-CSO-HFIB2-12, NanoString Technologies, WA, USA) consists of 770 genes related to the initiation of damage, inflammation, proliferation, and fibrosis modification which included CXCL10. RNA samples were loaded on nCounter cartridges (cat no.: SPRINT-CAR-1.0, 12 samples) and the nanoString machine was run overnight. nSolver V4 (NanoString Technologies, WA, USA) and Rosalind (Rosalind Bio, CA, USA) software were used for data analysis. Access to full data of the nanoString study will be provided on a subsequent publication and/or upon reasonable request. Since this is an exploratory study, no official sample size was calculated. The pragmatic sample size we report here is dependent on both the availability of samples and the cartridge size of the nanoString (12 samples per cartridge). The manufacturer quality control analysis red-flagged one sample from the inflammatory group which was therefore excluded from the analysis, based on manufacturer recommendation. Clinical characteristics of the donors can be found in Supplementary Table S2).

### Study- 4

#### Stimulation of normal lung fibroblasts

Primary human parenchymal lung fibroblasts were obtained from lung tumor resection surgery (left-over/waste material) as far as possible from the tumor and checked as normal by a pulmonary pathologist. The fibroblasts were cultured using HAM's F-12 culture media (cat no.: 21127030, Gibco, NY, USA) + 10% heat-inactivated Fetal Calf Serum (FCS) + 1% l-Glutamine (cat no.: 25030081, Gibco, NY, USA) + 1% Penicillin/streptomycin (cat no.: 10378016, Gibco, NY, USA) until 90% confluency was reached. 0.5 × 10^6^ cells were seeded per well in 6-well plates (passage number 5–7). After 24 h, fibroblasts were stimulated (in duplicate) with 5% BAL fluid or 0.5% serum, in complete culture media, obtained from SSc without ILD patients or patients with SSc-ILD (BAL fluid and serum were obtained from one patient/group/experiment). Clinical characteristics of these patients can be found in Supplementary Table S3.

In a different experiment, the fibroblasts were stimulated with 10 ng/ml or 100 ng/ml of human recombinant IL-6 protein (cat no.: 200-06, Peprotech, NJ, USA), or 10 ng/ml of human recombinant TGF-β (cat no.: 100-21, Peprotech, NJ, USA), or both. Controls were composed of wells treated with complete culture medium only (CTRL) or serum from healthy non-smoking volunteers without chronic illnesses or current use of medications (pool serum). After 4 h of incubation, fibroblasts were harvested in TRIzol (cat no.: 15596-026, Invitrogen, CA, USA) and stored at −80 °C until further analysis.

#### Reverse transcriptase—quantitative polymerase chain reaction (RT-qPCR) analysis

Total RNA was extracted using chloroform (cat no.: 67-66-3, Merck, Darmstadt, Germany) to collect the aqueous phase then precipitate RNA using isopropanol/70% ethanol (cat no.: 67-63-0 and 64-17-5, Merck, Darmstadt, Germany). After DNAse digestion, RNA quantity and quality were assessed using NanoDrop 1000 spectrophotometer (Thermo Scientific, DE, USA). RNA sample quality (260/280) > 1.8 was sufficient to proceed with the analysis. Thereafter, 100 ng RNA were utilized to synthesize cDNA using M-MLV reverse transcriptase (cat no.: 28025-13, Invitrogen, CA, USA) and Oligo (dT) 12–18 primer (cat no.: 18418012, Invitrogen, CA, USA). After cDNA synthesis, 1 μL of each sample was pipetted (*in duplicate*) into a 384-well plate. Next, mRNA levels of connective tissue growth factor (ctgf), α-smooth muscle actin (αsma), and C-X-C motif chemokine ligand 10 (CXCL10), transforming growth factor-β (tgfβ) and glyceraldehyde-3-phosphate dehydrogenase (gapdh) were measured by an Applied Biosystems™ QuantStudio™ 6 Flex Real-Time PCR System (Singapore) with specific TaqMan assays (αsma [acta 2, Hs00909449_m1], ctgf [Hs00170014_m1], cxcl10 [Hs00171042_m1], and tgfβ1 [Hs00998133_m1], Applied Biosystems, Warrington, UK). The amount of target gene wasnormalized to an endogenous reference gene (gapdh [Hs99999905_m1]) and expressed as relative expression (2–ΔCT) or as fold induction compared to an unstimulated sample (2-ΔΔCT). Data were analyzed using QuantStudio Real-Time PCR software v1.3 (Applied Biosystems, Singapore).

### Sample size calculations

#### Study-1

Based on an anticipated incidence of 10% in 5 years of SSc-ILD, we calculated that a total of at least 100 patients without ILD at baseline were needed to perform a log-rank test.^24^

#### Study-2

Since previous studies have not investigated the levels of CXCL10 in BAL fluids of patients with SSc-ILD, an exact sample size could not be calculated. The original protocol was based on an earlier study in patients with severe SSc-ILD compared to those without severe SSc-ILD with an effect size of 1.7, which would necessitate a sample size of 8 patients with SSc-ILD and 4 SSc patients without ILD.^26^ Additionally, Kameda et al. 2020^27^ showed an effect size of 2.0 for CXCL10 between CTD-ILD and IPF. Based on this study, we anticipated that inclusion of 7 SSc patients in both groups would be sufficient to achieve a power of 90% at an alpha of 0.05.

#### Study-3

This is an exploratory study. Hence, no formal sample size was calculated. The pragmatic sample size we report here is dependent on both the availability of samples and the cartridge size of the nanoString (12 samples per cartridge).

#### Study-4

No formal sample size was calculated since this is an *in vitro* study. Three technical replicates were performed per experiment.

### Outcomes

The outcomes evaluated in this study included: Study-11^1^: Association of CXCL10 serum levels with new onset of ILD in patients with SSc, as a primary outcome.^2^ Baseline levels of CXCL10 and differences in serum levels of SSc, SSc-ILD and healthy controls.^3^ Correlation of baseline CXCL10 serum levels with lung function tests including FVC and DL_co_. Study-24^4^: CXCL10 levels in BAL fluid in SSc patients without ILD and patients with SSc-ILD.^5^ Correlation between serum and BAL fluid CXCL10 levels in SSc patients without ILD and SSc-ILD. Study-36^6^: Gene expression levels of CXCL10 in early inflammatory lung sections compared to late fibrotic sections. Study-47^7^: Gene expression levels of inflammatory (cxcl10), early fibrotic (ctgf) and fibrotic (tgfβ and αsma) in primary human normal lung fibroblasts after stimulation with BAL fluid or serum from patients with SSc or SSc-ILD.^8^ cxcl10 mRNA level after IL-6 cytokine treatment in lung fibroblasts.

### Statistical analysis

Statistical analyses were performed using IBM SPSS Statistics version 28, R statistical software V4.3.0 and Prism GraphPad V8. Patient characteristics are presented as means with standard deviations, median with interquartile range, and counts followed by percentages, where appropriate.

### Study-1

Baseline serum CXCL10 levels in patients with SSc with and without ILD, and healthy controls are reported as medians followed by interquartile range. Statistical differences between the groups were tested using the Mann–Whitney U tests, with additional bootstrapping. *P*-values were reported with 95% confidence intervals of the bootstrapped *P*-values. Additionally, bootstrapped differences in the medians were calculated to demonstrate the magnitude in difference. Differences in medians are reported with 95% confidence intervals. All bootstrapping analyses were conducted with 50,000 iterations.

Associations between log₁₀(CXCL10) and ILD were additionally tested using logistic regression to assess the direction and strength of association. Moreover, multivariate analyses were conducted to test for the independent association of CXCL10 with ILD. Confounders from literature were selected and evaluated in multivariate models, using stepwise backward regression and AIC criteria. Unadjusted and adjusted odds ratios with 95% confidence intervals were calculated.

Spearman's correlation coefficients were calculated for correlations between CXCL10 serum levels and FVC, and CXCL10 and DL_co_. Additionally, linear regression models were fitted stratified by ILD to assess direction and magnitude of association of CXCL10 in patients with or without ILD. Multivariate models were fitted adjusting for potential confounders, models were selected based on stepwise backward regression, using AIC criteria.

In patients without ILD at baseline, the association between high (>median) or low (<median) CXCL10 levels at baseline and new onset ILD was calculated using the Log-rank (Mantel-cox) test. The relationship between high or low CXCL10 and new onset ILD is visualized in a Kaplan–Meier curve.

### Study-2

Spearman's correlation coefficients were performed to assess correlations between serum and BAL fluid CXCL10 levels. Baseline CXCL10 levels in BAL fluid of patients with and without ILD were compared using the same methods as study-1 for serum CXCL10.

### Study-3

For the nanoString study, nSolver and Rosalind software were used for data and statistical analyses. *P*-value adjustment is performed using the Benjamini-Hochberg method of estimating false discovery rates (FDR). Adjusted *P*-values <0.05 were considered significant.

### Study-4

Statistical differences between the groups were tested using the Mann–Whitney U tests. *P*-values are reported with 95% confidence intervals.

### Ethics

All studies have complied with the ethical approvals provided below:

Study-1: According to Dutch law, this study was not subject to the Human Subjects Act (WMO, in Dutch). Additionally, the local ethics committee of the University Medical Center Groningen determined that the study is exempt from written informed consent (2019.00260).

Study-2: The study was approved by the local ethics committee of The University Medical Center Groningen (NL68835.042.20) and written informed consent was obtained from each subject prior to recruitment.

Study-3: According to Dutch law, this study was not subject to the Human Subjects Act (WMO, in Dutch). We obtained a favorable opinion from the local review committee of the University Medical Center Groningen (2019.00015).

Study-4: Fibroblasts were cultured from left-over materials from cancer lung resections. According to Dutch law, left-over/waste materials do not need an approval from a medical ethical committee.

### Role of funders

The funders had no role in the design of the study; in the collection, analyses, or interpretation of data; in the writing of the manuscript; or in the decision to publish the results.

## Results

### Study- 1

#### Patient characteristics

The clinical characteristics are presented in Table 1. At baseline, 124 patients of the SSc cohort did not have ILD while 41 patients already had developed ILD. In the ILD subgroup, % (predicted) FVC and DL_co_ were lower and immunosuppression medication usage was more common compared to SSc patients without ILD.

**Table 1:** Clinical characteristics at baseline (study-1).

	SSc patients without-ILD (N = 124)	SSc patients with-ILD (N = 41)	HC (N = 13)
Age in years, median (IQR)	61 (54–72)	65 (53–72)	53 (47–57)
Female, N (%)	102 (82.3)	28 (68.3)	8 (61.5)
Extent of skin involvement, n (%):			
LcSSc	114 (91.9)	35 (85.4)	
dcSSc	9 (7.3)	6 (14.6)	
Sine scleroderma	1 (0.8)	0 (0.0)	
Puffy fingers or sclerodactyly, n (%)	103 (83.1)	35 (85.4)	
Pitting scars or digital ulcers, n (%)	66 (53.2)	17 (41.5)	
Telangiectasia, n (%)	83 (74.1) (ND: 12)	29 (78.4) (ND: 4)	
Raynaud's phenomenon, n (%)	124 (100.0)	40 (97.6)	
Autoantibody profile, n (%):			
Anti-nucleolar antibody	29 (23.4)	22 (53.7)	
Anti-centromere	74 (59.7)	8 (19.5)	
Anti-topoisomerase I	10 (8.1)	8 (19.5)	
Anti-RNA polymerase 3	1 (0.8)	0 (0.0)	
Other	10 (8.1)	3 (7.3)	
Calcinosis cutis, n (%)	40 (36.4) (ND: 14)	11 (32.4) (ND: 7)	
Gastrointestinal involvement, n (%)	88 (73.9) (ND: 5)	25 (62.5) (ND: 1)	
HRCT Pattern, (n)			
iNSIP	–	7	
fNSIP	–	26	
UIP	–	6	
Other	–	2	
PFTs, median (IQR):			
% FVC	109.0 (95.5–120.0) (ND: 15)	94.5 (68.8–107.0) (ND: 3)	
% DLCO	74.0 (62.0–83.5) (ND: 15)	54.0 (42.5–70.5) (ND: 9)	
COPD, n (%)	28 (22.6)	6 (14.6)	
Immunosuppression	17 (13.7)	15 (36.6)	
Vasodilators	16 (12.9)	3 (7.3)	
Glucocorticoids	10 (8.1)	7 (17.1)	

lcSSc: limited cutaneous SSc; dcSSc: Diffuse cutaneous SSc; FVC: forced vital capacity, DL_co_: capacity for diffusion of carbon monoxide, COPD: chronic obstructive pulmonary disease; ND: not documented; iNSIP: inflammatory nonspecific interstitial pneumonia; fNSIP: fibrotic NSIP; UIP: usual interstitial pneumonia.

#### Serum CXCL10 concentrations are elevated in SSc-ILD patients

**Fig. 2:**
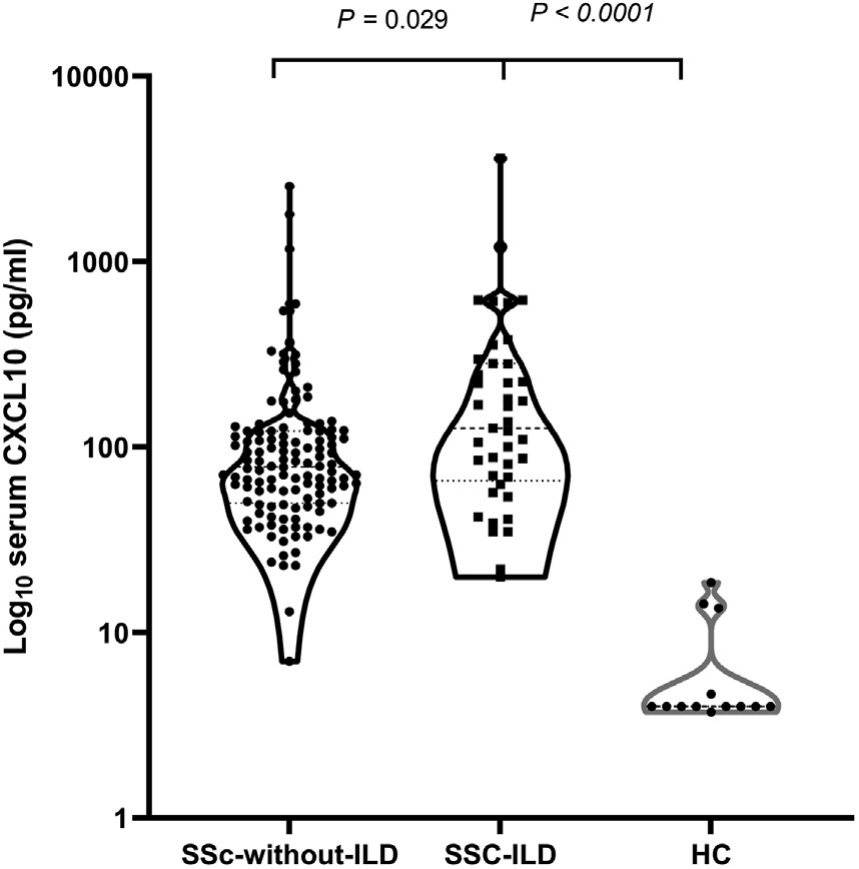
Systemic (serum) levels of CXCL10 in SSc without ILD (N = 124), SSc-ILD (N = 41) and healthy controls (N = 13). Patients with SSc with or without interstitial lung disease have statistically significant higher CXCL10 levels compared to healthy controls (Mann–Whitney U test). Patients with SSc-ILD had statistically significant higher levels of CXCL10 compared to SSc without ILD (Mann–Whitney U test). Measurements are shown as median values (dashed) with IQR (dotted) in pg/ml.

To evaluate the systemic concentration of CXCL10 in different groups, CXCL10 protein was assayed in the sera of patients with SSc-ILD, SSc without ILD, and healthy controls (Fig. 2). CXCL10 levels were higher in SSc without ILD patients [Median (IQR): 78.5 pg/ml (50–122)] compared to healthy controls [4 pg/ml (4–9.1), *P* < 0.0001, 95% CI: 3.3 × 10^−9^ to 7.9 × 10^−9^] and between patients with SSc-ILD and healthy controls [126 pg/ml (66–282.5), *P* < 0.0001, 95% CI: 6.4 × 10^−8^ to 7.2 × 10^−8^].

We also found higher levels in patients with SSc-ILD compared to patients with SSc but no ILD [126 pg/ml (66–282.5) vs. 78.5 pg/ml (50–122), *P* = 0.029, 95% CI: 1.5 × 10^−6^ to 0.43]. The difference in median CXCL10 levels between those with ILD and those without was 47.5 pg/ml with 95% CI: 5.5 to 142. See Supplementary Material for detailed statistical analyses (Supplementary 1).

#### Serum log₁₀CXCL10 concentrations are independently associated with baseline ILD

Log₁₀CXCL10 levels were associated with a 3.86 (1.56–9.18) increased odds ratio of ILD, *P* = 0.004. When adjusted for confounders (age, sex, anti-centromere autoantibody, SSc subtype, use of immunosuppressive medications and COPD), both in the complete-model and backward selected model, the association remained statistically significant [OR = 3.12 (1.24–8.55), *P* = 0.02]. Supplementary 2 shows the results of the univariate and multivariate models.

#### Serum CXCL10 concentration levels correlate negatively with lung function

**Fig. 3:**
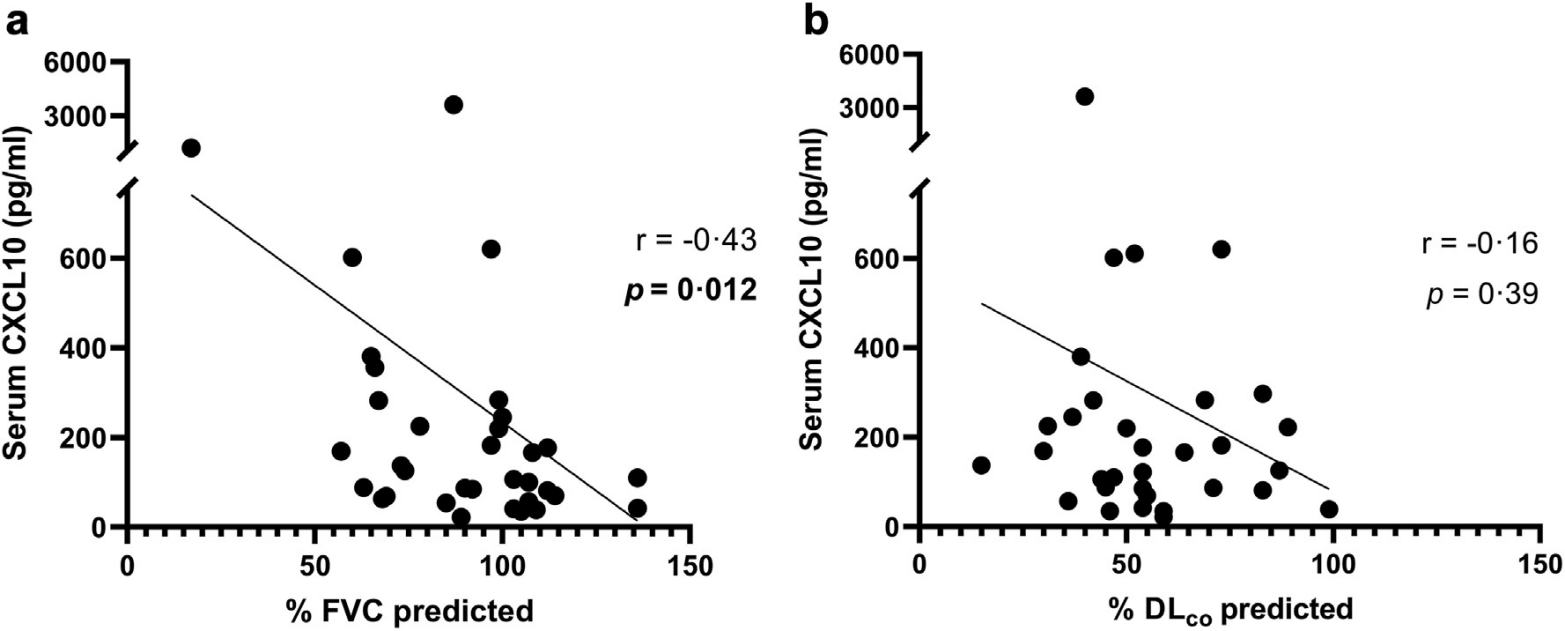
CXCL10 levels were negatively correlated with PFTs in patients with SSc-ILD. a) Spearman's correlation between serum CXCL10 and %FVC shows a moderate negative correlation (N = 38, r = −0.43, *P* = 0.012. b) Spearman's correlation between serum CXCL10 and %DLco indicates no correlation (N = 32). After adjusting for confounders, CXCL10 was negatively associated with %DLco. PFTs: pulmonary function tests, FVC: forced vital capacity, DLco: capacity for carbon monoxide diffusion.

To comprehensively examine whether systemic CXCL10 levels could be used as a proxy for deterioration of lung function in SSc-ILD, we correlated serum CXCL10 in patients with SSc-ILD with %FVC predicted and %DL_co_ predicted. Our results demonstrated that serum CXCL10 levels correlated negatively with %FVC predicted (*P* = 0.012, r = −0.43, 95% CI: −0.67 to −0.09) (Fig. 3a) but not with %DL_co_ predicted (*P* = 0.39, r = −0.16, 95% CI: −0.49 to 0.21) (Fig. 3b). To adjust for potential confounders, we performed a multivariate linear regression and found that %FVC predicted remained associated with CXCL10 levels (B = −19.6, SE = 8.47 and *P* = 0.04) both in the full and backward selected model. For %DL_co_ predicted, adjusting for potential confounders led to a change in the magnitude and *P* value with CXCL10 levels (B = −18.0, SE = 7.89 and *P* = 0.02). Supplementary 3 shows the results of the univariate and multivariate models.

When serum CXCL10 levels were correlated with the PFTs in SSc without ILD patients, we found that %DL_co_ had a statistically significant, though weak negative correlation with CXCL10 (r = −0.19, *P* = 0.047). When adjusting for confounders, the %DL_co_ predicted association with CXCL10 levels was not significant (B = −2.92, SE = 4.32 and *P* = 0.50). For %FVC predicted, no correlation was found even after adjusting for confounders. Supplementary 3 shows the results of the univariate and multivariate models.

#### Follow-up data revealed the potential of higher serum levels of CXCL10 to be associated with new onset of ILD

**Fig. 4:**
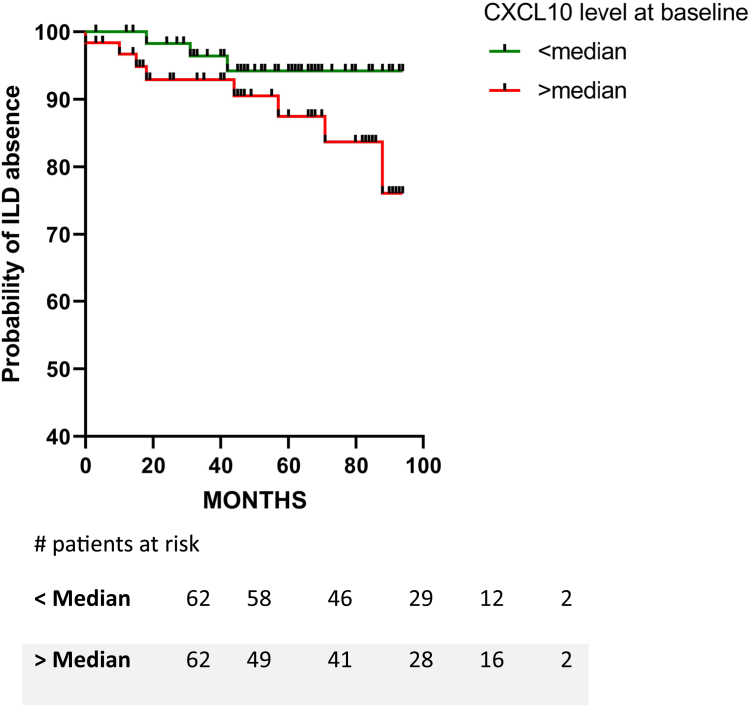
Higher CXCL10 may be associated with long-term ILD development. Kaplan–Meier survival curve according to baseline median CXCL10 concentration. CXCL10 levels >78.5 pg/ml shows a 2.74-fold increased hazard of new onset of ILD [Log-rank (Mantel-cox) test, *P* = 0.119].

Patients who did not have ILD at baseline (N = 124) were followed for a median of 56 months (range = 33–82 months). During this period, 11 patients (9%) developed ILD. When stratifying the new ILD developers according to CXCL10 median levels at baseline (below and above median CXCL10 levels), we found that CXCL10 levels >78.5 pg/ml were associated with increased hazard of developing new onset of ILD [HR = 2.74, 95% CI (0.78 to 8.42)]. Log-rank (Mantel–Cox) *P* = 0.119 (Fig. 4).

### Study- 2

#### BAL CXCL10 level is higher in patients with SSc-ILD and correlate with serum levels

**Fig. 5:**
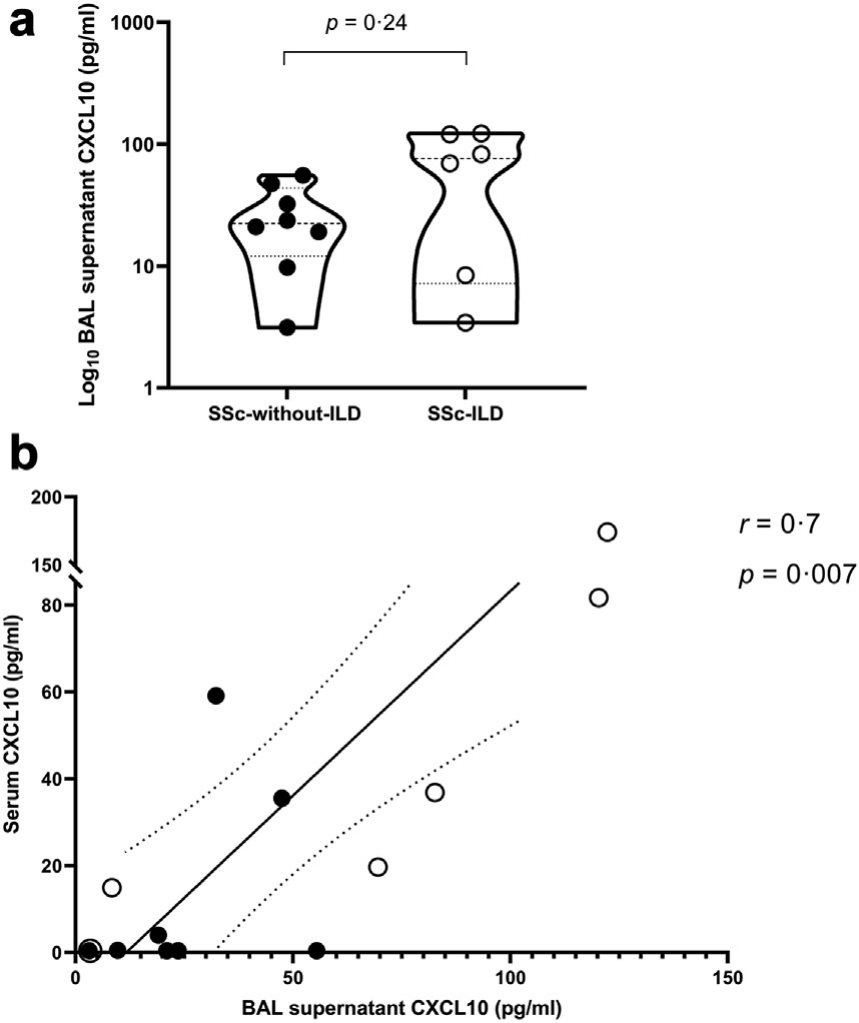
Local CXCL10 concentration levels and correlation between local and systemic CXCL10 levels. a) Comparison between CXCL10 concentration levels in BAL supernatant between SSc-without-ILD (N = 8) and patients with SSc-ILD (N = 6) (Mann–Whitney U test, *P* = 0.23). Measurements are shown as median values (dashed) with IQR (dotted) in pg/ml. b) Spearman's correlation between CXCL10 concentration levels in serum and BAL supernatant in the same patients showing statistical significance *P* = 0.007 and r = 0.7 (N = 14). Measurements are in pg/ml.

To investigate the differences in CXCL10 concentrations locally in the lung, CXCL10 levels were measured in BAL supernatants from patients with SSc without ILD and SSc-ILD [Median (IQR): 22.3 pg/ml (12.1–43.7) vs. 76.1 pg/ml (7.2–120.8), *P* = 0.24, 95% CI: −19.5 to 100]. The data show a trend of CXCL10 increase in BAL supernatant of patients with SSc-ILD compared to SSc patients without ILD (Fig. 5a). See Supplementary Material for detailed statistical analyses (Supplementary 4).

Next, CXCL10 protein levels in BAL and serum of the patients were compared (Fig. 5b). Data demonstrated a positive correlation between CXCL10 in BAL supernatant and serum (*r* = 0.7, *P* = 0.007, 95% CI: 0.25 to 0.90).

### Study- 3

#### Transcriptomic analysis of SSc-ILD lung tissue reveals overexpression of CXCL10 in inflammatory sections

**Fig. 6:**
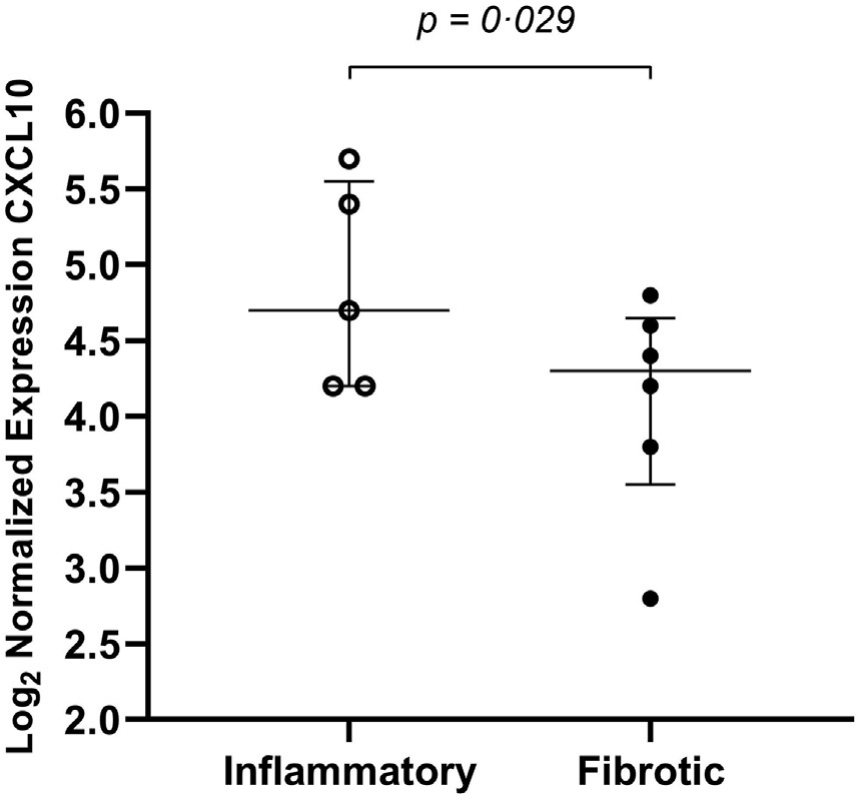
CXCL10 gene expression in SSc-ILD inflammatory and fibrotic regions. Transcriptomic analysis showed that CXCL10 is 2.3x more highly expressed in inflammatory regions compared with fibrotic regions in patients with SSc-ILD [*P* = 0.029 (t-test)]. N = 11 (5 inflammatory SSc-ILD regions and 6 fibrotic SSc-ILD regions).

By investigating FFPE lung samples of SSc-ILD tissues we wanted to detect differential gene expression between early and late phases of ILD reflected by inflammatory and fibrotic lesions. This may help to better stage and group patients with SSc-ILD with the main aim of finding novel biomarkers. We investigated changes in CXCL10 expression between early (inflammatory) and late (fibrotic) stages of lung tissues. Our data show overexpression of CXCL10 in inflammatory regions compared to fibrotic regions in SSc-ILD lung tissues [Median (IQR): 4.7 (4.2–5.6) vs. 4.3 (3.6–4.7), *P* = 0.029] (Fig. 6).

### Study- 4

#### SSc-ILD serum and BAL induce mRNA expression of cxcl10 and ctgf in lung fibroblasts

The effect of exposure of normal parenchymal human primary lung fibroblasts to BAL supernatant or serum from patients with SSc-ILD, SSc without ILD patients, or serum from healthy controls was studied. A set of genes that represents early inflammation (CXCL10), early fibrosis (ctgf) and late fibrosis (tgfβ and αsma) processes was used.

**Fig. 7:**
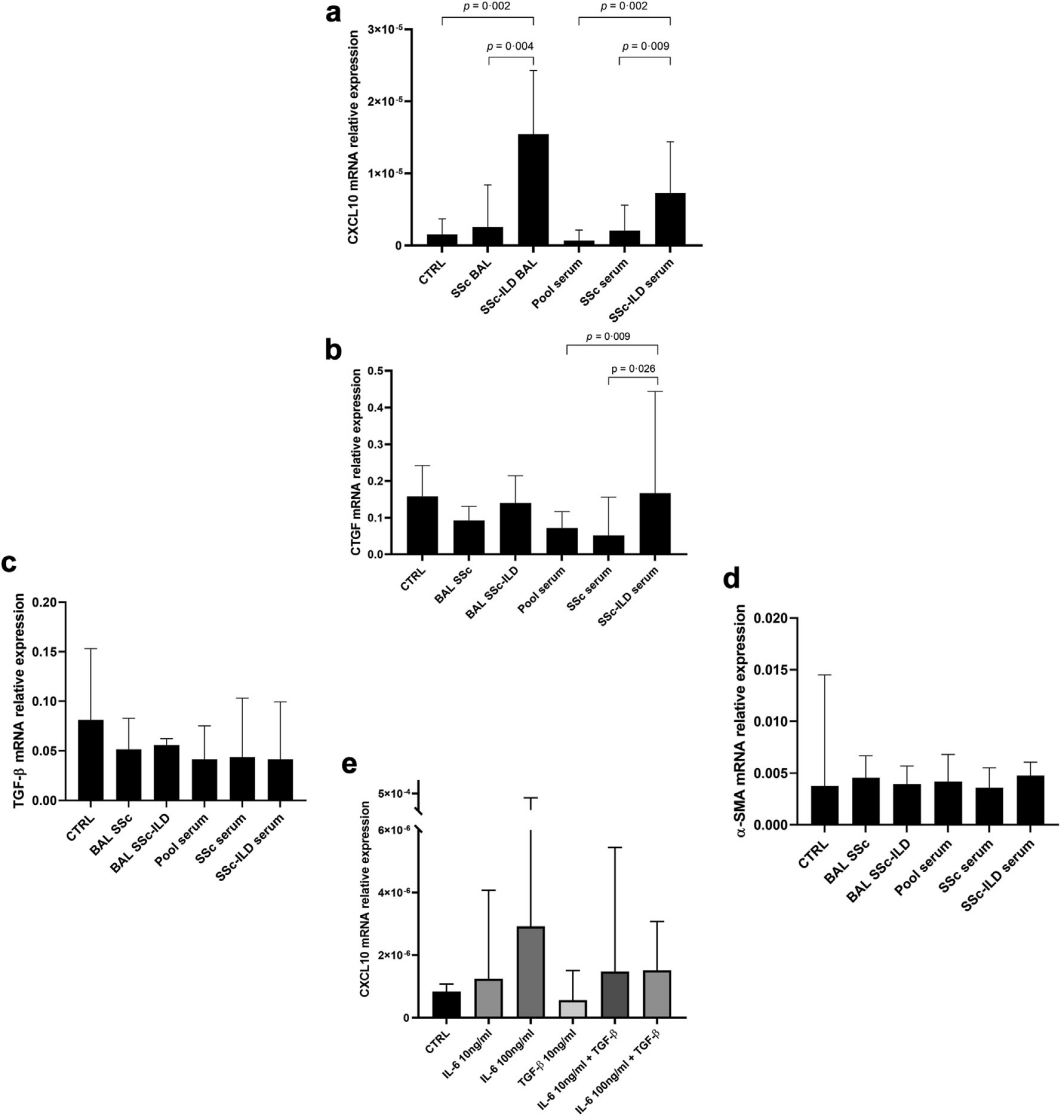
Stimulation of normal human lung fibroblasts with SSc fluids (a–d), or inflammatory or fibrotic cytokines (e). (a–d) 5% BAL supernatant from SSc without ILD (BAL SSc) or SSc-ILD (BAL SSc-ILD), or 1% serum from SSc without ILD (SSc serum) or SSc-ILD (SSc-ILD serum) or healthy control (Pool serum) was used to stimulate lung fibroblasts. (a) SSc-ILD BAL and serum-treated fibroblasts overexpressed cxcl10 compared to SSc without ILD BAL (Mann–Whitney U test, *P* = 0.004) and serum (Mann–Whitney U test, *P* = 0.0087). Also, SSc-ILD BAL-treated fibroblasts had higher cxcl10 levels compared to CTRL (Mann–Whitney U test, *P* = 0.0022). Fibroblasts treated with SSc-ILD serum significantly overexpressed cxcl10 compared to fibroblasts treated with healthy serum (Mann–Whitney U test, *P* = 0.0022). SSc-ILD serum-treated lung fibroblasts overexpressed ctgf expression compared to SSc without ILD and pool sera (Mann–Whitney U test, *P* = 0.026 and *P* = 0.0087, respectively). (c–d) No statistically significant differences in tgfβ or αsma expression when lung fibroblasts were treated with BAL or serum (Mann–Whitney U test). The results are medians of three technical experiments (n = 3) where 6 patients' biofluids were used (N = 6). (e) IL-6 (10 ng/ml or 100 ng/ml) or TGF-β (10 ng/ml) or both were used to stimulate lung fibroblasts for 4 h. IL-6 stimulated cxcl10 expression in lung fibroblasts in a concentration-dependent manner. The higher concentration of IL-6 (100 ng/ml) showed increase of cxcl10 expression with a trend towards statistical significance of *P* value = 0.055 vs. CTRL (Mann–Whitney U test). The results are medians with IQR of three technical experiments (n = 3). CTRL: culture media without stimulant.

As can be seen in Fig. 7a, cxcl10 relative gene expression was higher in fibroblasts treated with BAL obtained from patients with SSc-ILD [Median (IQR): 1.5 × 10^−5^ (1.1 × 10^−5^ to 2.4 × 10^−5^)] compared to BAL from SSc without ILD patients [2.6 × 10^−6^ (1.4 × 10^−6^ to 4.8 × 10^−6^)], *P* = 0.004, 95% CI: 5.2 × 10^−6^ to 2.1 × 10^−5^. Similarly, in fibroblasts treated with serum obtained from patients with SSc-ILD [7.3 × 10^−6^ (4.7 × 10^−6^ to 1.3 × 10^−5^)] cxcl10 was higher compared to those without ILD [2.1 × 10^−6^ (1.6 × 10^−6^ to 4.0 × 10^−6^)], *P* = 0.009, 95% CI: 2.1 × 10^−6^ to 1.1 × 10^−5^].

Also, cxcl10 relative gene expression was higher in fibroblasts treated with BAL obtained from SSc-ILD patients [1.6 × 10^−5^ (1.1 × 10^−5^ to 2.4 × 10^−5^)] compared to controls [1.6 × 10^−6^ (5.6 × 10^−7^ to 3.3 × 10^−6^), *P* = 0.002, 95% CI: 6.8 × 10^−6^ to 2.3 × 10^−5^]. Same for serum obtained from SSc with ILD patients [7.3 × 10^−6^ (4.7 × 10^−6^ to 1.3 × 10^−5^)] compared to pool healthy serum [6.5 × 10^−7^ (5.6 × 10^−7^ to 1.8 × 10^−6^), *P* = 0.002, 95% CI: 3.6 × 10^−6^ to 1.3 × 10^−5^].

ctgf gene expression was induced in lung fibroblasts exclusively using serum from patients with SSc-ILD [0.1666 (0.1057–0.3611) compared to treatment with serum from SSc without ILD [0.0516 (0.0281–0.1534), *P* = 0.026, 95% CI: 0.0112 to 0.3048] or healthy pooled serum [0.0718 (0.018–0.0879), *P* = 0.009, 95% CI: 0.0339 to 0.3275]. No induction of ctgf gene expression was observed in lung fibroblasts treated with serum from SSc without ILD and controls (Fig. 7b). No effects were observed on tgfβ or αsma expression by BAL or serum-treated lung fibroblasts (Fig. 7c–d).

#### IL-6 induces CXCL10 expression in lung fibroblasts

We treated lung fibroblasts with human recombinant IL-6 and used human recombinant TGF-β as a positive control to test which downstream processes (inflammatory or fibrotic, respectively) CXCL10 follows. Our data showed that IL-6 treatment, but not TGF-β, led to the overexpression of cxcl10 in lung fibroblasts in a concentration-dependent manner compared to control (Fig. 7e).

## Discussion

In this study, we showed that CXCL10 protein levels are elevated in systemic (serum) and local (lung) compartments of SSc-ILD compared to SSc without ILD patients, and healthy controls. Importantly, these findings show that serum CXCL10 levels in patients with SSc-ILD correlate with decreased FVC and DL_co_, and high levels may be associated with new onset of ILD in patients with SSc. At the tissue and gene level, we demonstrated that CXCL10 was overexpressed in the inflammatory regions of SSc-ILD lung tissues compared to the fibrotic regions. *In vitro* experiments showed that stimulating normal primary human lung fibroblasts with SSc-ILD serum and BAL fluids induce CXCL10 expression compared to controls and the IL-6 axis may be an alternative or parallel pathway for this activation. These findings suggest the involvement of CXCL10 in SSc-ILD pathobiology.

The elevated systemic CXCL10 levels are in line with previous data showing that 15 out of 29 patients with SSc-ILD had a statistically significant higher systemic CXCL10 level compared to SSc patients without ILD.^21^ In a different study, involving a cohort of 143 patients with SSc at different stages (early, non-fibrotic, limited cutaneous SSc, or diffuse cutaneous SSc), it was demonstrated that higher levels of CXCL10 could predict a shorter time until SSc progression. Among other SSc progression events, higher CXCL10 levels predicted worsening of lung function.^28^^,^^29^ Another study investigated serum CXCL10/11 in very early diagnosis of systemic sclerosis (VEDOSS) and patients with SSc. Data showed that patients with SSc had higher CXCL10 serum levels compared to VEDOSS. Additionally, baseline CXCL10 levels were higher in VEDOSS who shifted to SSc than those who did not shift to SSc. Moreover, receiver-operating characteristics (ROC) analyses showed that CXCL10/11 can discriminate VEDOSS subjects shifting to SSc disease.^30^ In our study, we showed that high systemic CXCL10 levels may be associated with a higher risk to develop a new onset of ILD in patients with SSc. Although the log-rank *P* value only showed trend towards statistical significance, there is almost three times increased risk of developing new onset of ILD in patients with SSc who have CXCL10 levels >78.5 pg/ml. These data also show that SSc-ILD begins with an inflammatory signature as evidenced by the high CXCL10 levels.

In a longitudinal study, Antonelli et al. (2008) demonstrated that newly diagnosed patients with SSc had statistically significant higher systemic CXCL10 levels at baseline than 5 years later. They also reported that patients with SSc-ILD had statistically significant higher CXCL10 levels compared to SSc without ILD. Interestingly, when they compared CXCL10 to CCL2 (chemoattractant related to Th2 activation) levels at baseline vs. follow-up, they found that CXCL10 levels were more prominent in early SSc while CCL2 became dominant later. This shift is due to the decrease of CXCL10 levels on follow-up while CCL2 remained unchanged.^20^ This outcome supports the notion that SSc develops from an inflammatory Th1-like phase to a fibrotic Th2-like phase over time.^20^ In the nanoString study in SSc-ILD lung tissues (unpublished), we found a statistically significant overexpression of CXCL10 mRNA in inflammatory vs. fibrotic lung tissue. This is in line with the observations of the Antonelli group, however, our results are generated at tissue and gene levels.

We measured the levels of CXCL10 in BAL supernatants to understand whether local CXCL10 levels are related to those in serum and potentially have a role in altering the immune homeostasis of the lungs. A recent study sought to correlate CXCL10 levels in BAL supernatant with serum but in a heterogeneous cohort of 16 patients with collagen vascular diseases-interstitial lung disease (CVDs-ILD) of which 5 were patients with SSc-ILD. They reported a weak correlation between BAL supernatant and serum CXCL10 levels.^27^ However, in our cohort of 15 patients with SSc who underwent a BAL procedure, we demonstrated that systemic CXCL10 levels correlate with the levels in the BAL. This discrepancy might be due to the relatively limited sample size and the heterogeneous nature of the ILD cohort reported in their study. Although speculative, our results suggest that serum CXCL10 may be a proxy reflecting locally produced CXCL10 in lungs, especially in patients with SSc-ILD. However, this will need confirmation in a larger patient group.

Regarding the clinical relevance of our data, we found that higher systemic CXCL10 levels in patients with SSc-ILD correlate with worsening FVC. Initially, CXCL10 levels were not associated with %DL_co_ predicted in patients with SSc-ILD while they were negatively associated with %DL_co_ predicted in SSc without ILD patients. However, after adjusting for confounders, the association in patients with SSc-ILD appeared while it disappeared in the SSc patients without ILD. These data are in line with what Kameda et al. reported in their heterogeneous cohort of patients with CVDs-ILD.^27^ Additionally, other studies have shown a negative correlation between lung function (FVC, DL_co_, total lung capacity, and vital capacity) and CXCR3 expression, the receptor for CXCL10, on monocytes and a positive correlation between CXCR3 and CXCL10 in patients with non-IPF disease.^31^^,^^32^ Taken together, the studies discussed including the results provided in our current study strongly support that an activated CXCL10-CXCR3 axis involving high levels of systemic and local CXCL10 correlates with worse lung function. Furthermore, it was shown that SSc-ILD patient-derived serum and BAL fluid induced CXCL10 expression in normal primary lung fibroblasts. On the other hand, CXCL10 expression was not induced using serum or BAL fluid derived from SSc patients without ILD. This activation of CXCL10 pathways in the fibrosis effector cells may lead to recruitment of more fibrotic cells and other inflammatory cells that could induce persistent fibrosis.^33^ Importantly, SSc-ILD serum induced the expression of the early fibrosis marker ctgf but not tgfβ or α-sma which may be interpreted as an early sign of fibrosis. This is in line with the overexpression of CXCL10 and its association with new ILD incidence.^34^

IL-6 is a pleiotropic inflammatory cytokine that has been shown to be one of the main driving forces of SSc and SSc-ILD early pathogenesis.^35^^,^^36^ When the IL-6—CXCL10 axis is activated, IL-6 overproduction could lead to excessive CXCL10 release as a downstream process.^37^ Our *in vitro* data show that IL-6 can induce lung fibroblasts to express more CXCL10 mRNA which may indicate an alternative/parallel pathway other than the canonical IFN-γ pathway.^38^^,^^39^ These data support the use of Tocilizumab, an IL-6R antagonist, to block the downstream signalling pathway including, but not limited to, CXCL10 overexpression. Additionally, upstream of CXCL10 overexpression is the activation of Janus Kinase (JAK) pathway(s) through IFN-γ—IFN-γR complex formation.^40^ Blocking this pathway using JAK inhibitors is gaining momentum in the treatment of SSc.^41^, ^42^, ^43^ JAK inhibitors have the potential to block inflammatory and fibrotic pathways and have proven efficacies in other autoimmune diseases.^44^, ^45^, ^46^ Future research is needed to unravel the effect of these inhibitors on CXCL10 expression.

Although this study has considerable potential in unraveling the relationship between CXCL10 and SSc-ILD by combining clinical (systemic and local), transcriptomic and *in vitro* investigations, there are some limitations. First, the sample sizes in study 1 and study 2 limit robust statistical analyses, particularly for the difference in CXCL10 levels in BAL fluid between SSc-ILD and SSc without ILD. Additional analyses adjusting for confounders were not possible. Similarly, in the analysis with new onset ILD, potential confounders could not be included. Therefore, the interpretation of the survival analysis must be taken with caution.

Second, the retrospective nature of the study meant that measurements were limited to data collected and recorded in electronic medical records. As a result, important confounders such as smoking, disease duration, mRSS could not be included. Moreover, at the time of inclusion, our center followed a standard stepwise protocol based on PFTs. Only in patients with an FVC <70% predicted and/or DLco <80% predicted, an HRCT was performed, while in those with normal values, HRCT was omitted. However, the current standard of care is to perform an HRCT in every newly diagnosed patient with SSc. This protocol might have led to missing some patients with ILD (selection bias).

Third, it was not possible to precisely establish the CXCL10 concentration in the lung since the quantification was based on the lavage volume. Additionally, the biological relevance of the correlation between serum and BAL fluid should be interpreted carefully since the source of systemic CXCL10 can be from tissues other than the lungs.^47^^,^^48^

Fourth, SSc-ILD pathogenesis is complex and involves various cell types that affect each other in para- and autocrine manners which is challenging to mimic *in vitro.*^49^ Additionally, due to the single-cell type *in vitro* model we used, long-term effects on fibroblasts could not be shown. Finally, although we have not definitely answered whether blocking IL-6 signalling would inhibit the overexpression of CXCL10 in lung fibroblasts, we are positive that future studies will be able to unravel this possible interplay.

In conclusion, our survival analysis suggests that patients with SSc with high CXCL10 may develop ILD in the future. Although it needs to be confirmed in bigger studies, it appears that systemic levels of CXCL10 can mirror lung CXCL10 levels, and it would be worth investigating whether it could serve to monitor possible loss of lung function. It seems that not only lung fibroblasts play a major role in SSc-ILD fibrogenesis, but also in earlier inflammatory phases through overexpressing cxcl10 and ctgf, and recruiting inflammatory immune cells and fibrotic effector cells. Future studies should focus on finding a cut-off value for CXCL10 concentration levels in a prospective cohort to precisely predict which patients with SSc may develop ILD. This will potentially allow early detection and treatment for such a serious complication.

## Contributors

Conceptualization, YA, JW, HvG and DJM; methodology, YA, JW and DJM.; validation, YA, RDMV, IMA, BD-vdM, MJvdL, BJK, AS, WT, CTG, HvG, JW and DJM; formal analysis, YA, RDMV, IMA, DJM; investigation, YA, IMA, HvG, JW and DJM; resources, YA, IMA, BDvdM, MJvdL, BJK, AS, WT, CTG, HvG, JW and DJM; data curation, YA and IMA; writing—original draft preparation, YA; writing—review and ed-iting, YA, RDMV, BJK, WT, HvG, CTG JW and DJM; visualization, YA, RDMV; supervision, DJM; project administration, YA; funding acquisition, DJM. All authors have read and agreed to the published version of the manuscript.

## Data sharing statement

The data underlying this article will be shared on reasonable request to the corresponding author with appropriate institutional review board approval.

## Declaration of interests

D.J. Mulder received grants from Sanofi Genzyme paid to the institution. W. Timens received consultancy fees from Merck Sharp Dohme and Bristol-Myers Squibb. The rest of the coauthors declare no conflict of interest.

## Appendix A Supplementary data

Supplementary Materials

Protocol study 1 Retrospective cohort Non-WMO

Protocol appendix study 2&4

Protocol appendix study 3

Publication License

RNA Extraction to qPCR protocols

COA CXCL10 DY266_P294754
