# Supplementary material for: High serum C-X-C motif chemokine ligand 10 (CXCL10) levels may be associated with new onset interstitial lung disease in patients with systemic sclerosis: evidence from observational, clinical, transcriptomic and in vitro studies
Source: eBioMedicine. 2023 Nov 22;98:104883. doi: 10.1016/j.ebiom.2023.104883 (PMC10708993; doi:10.1016/j.ebiom.2023.104883)
Supplement: Supplementary Materials [file mmc1.docx]

**Supplementary Materials**

Table of content:

| Content | Page |
| --- | --- |
| Extended abstract | 1 |
| Supp 1: Detailed statistical analysis and bootstrapping for Study-1 | 5 |
| Supp 2: Multivariate linear regression between CXCL10 levels, ILD status and Important clinical variables | 7 |
| Supp 3: Multivariate linear regression between FVC or DLco, CXCL10 and important clinical variables | 8 |
| Supp Table 1: Clinical characteristics of SSc patients who underwent *BAL procedure (study-2)* | 12 |
| Supp 4: Detailed statistical analysis and bootstrapping for Study-2 | 13 |
| Supp Table 2: SSc patient information from whom lung tissue sections were obtained (Study-3) | 14 |
| Supp Table 3: Clinical information regarding patients from whom biomaterials were obtained and used (Study-4) | 15 |

1. Extended abstract:

**High serum C-X-C motif chemokine ligand 10 (CXCL10) levels could be associated with new onset of interstitial lung disease in patients with systemic sclerosis:**

**Observational clinical and transcriptomic studies supported by *in vitro* work**

1. **INTRODUCTION AND OBJECTIVES:**

Systemic sclerosis (SSc) is a fibroproliferative systemic auto-immune disease. Particularly in the early stages of the disease, inflammatory changes are present in the skin and internal organs. SSc-ILD is the leading cause of death in SSc patients. There is an unmet need for predictive biomarkers to identify SSc patients at risk of ILD. Previous studies have shown that IFN pathways may play a role in SSc. We investigated an IFN-γ -induced chemokine C-X-C motif chemokine ligand 10 (CXCL10) on a systemic, local and lung tissue levels and supported these observations by *in vitro* work. We also assessed whether CXCL10 is associated with new onset of ILD in SSc patients.


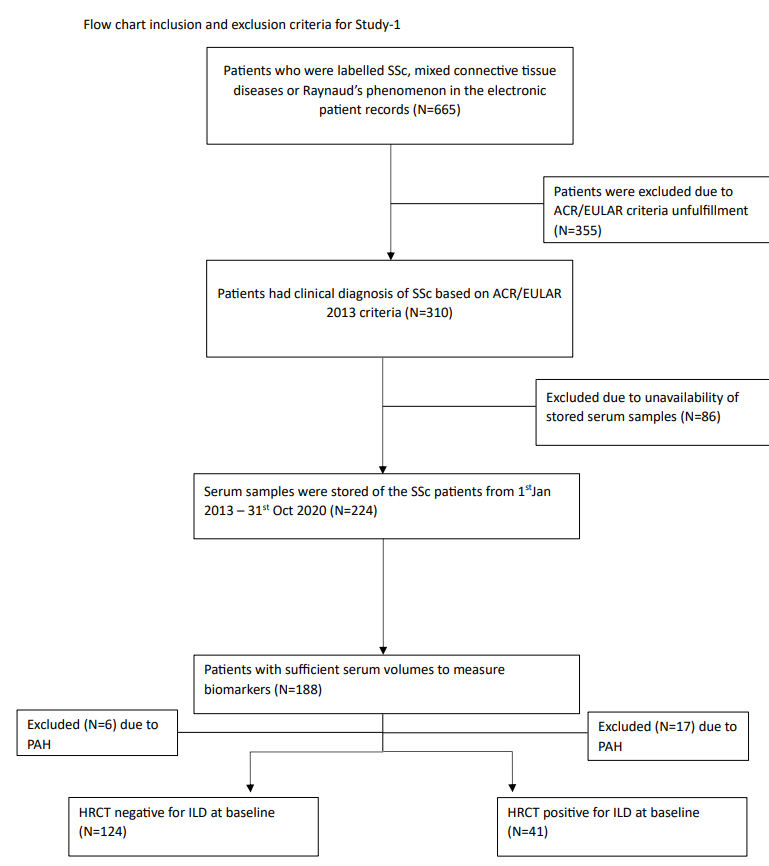


1. **MATERIALS AND METHODS**
   1. **Study design**

**We performed four interconnecting studies:**

Study-1: A retrospective clinical study with 165 patients followed from January 2013 until December 2020. Diagnosed SSc patients who fulfilled EULAR/ACR 2013 criteria and were between 18-70 years of age were included. Serum was collected and stored at -20°C. Pulmonary function tests (PFTs) including forced vital capacity (FVC) and diffusion capacity for carbon monoxide (DL_co_) were performed according to as standard of care ATS/ERS guidelines. At the time of inclusion, our centre followed a standard stepwise protocol based on PFTs. Only in patients with an FVC< 70% predicted and/or DL_co_< 80% predicted, an HRCT was performed, in those with normal values an HRCT was omitted. Importantly, interstitial lung disease (ILD) diagnosis was based on high resolution computed tomography scan (HRCT). Please refer to the Flowchart 1 for inclusion/exclusion criteria. Thirteen age- and sex- matched healthy controls were enrolled for blood samples. Regarding sample size, we anticipated the incidence of 10% in 5 years of SSc-ILD and according to this we calculated that a total of at least 100 SSc without ILD patients is needed at baseline to allow performance of log-rank test.

Flowchart 1: Inclusion and exclusion criteria for study-1.


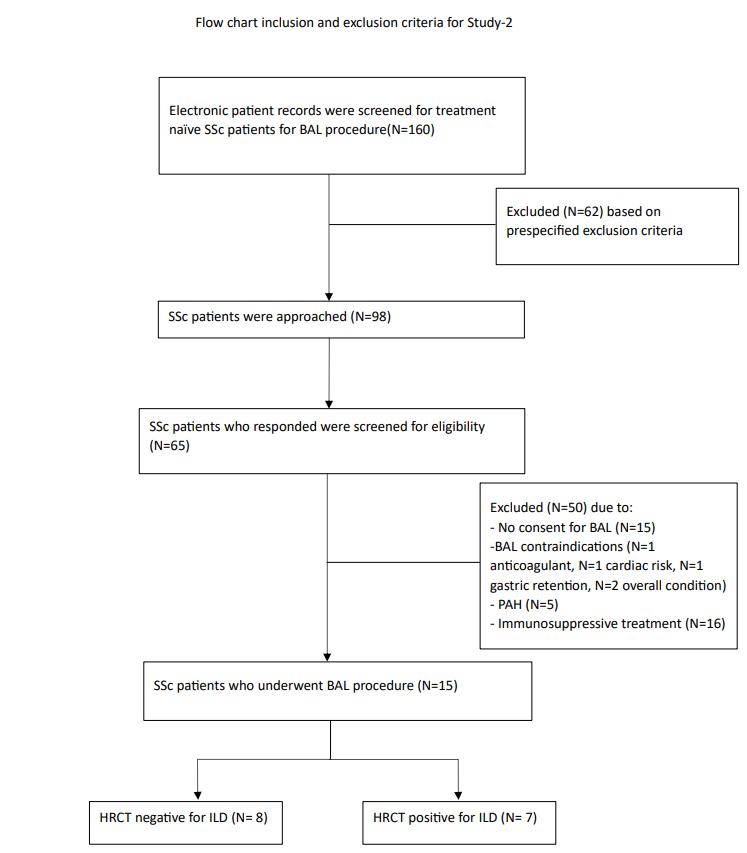
Study-2**:** A cross sectional study where we recruited 15 SSc-treatment naïve patients (7 SSc-ILD patients) from January 2021 until November 2021 for a bronchoalveolar lavage (BAL) procedure. Around 100-120mL of lavage volume was recovered from each patient. BAL was then aliquoted and saved at -20°C. SSc and SSc-ILD inclusion criteria follows Study-1 criteria except for that every SSc patient underwent an HRCT scan for ILD diagnosis confirmation. Flowchart 2 gives more information regarding inclusion and exclusion criteria. Since no previous study has investigated the levels of CXCL10 in BAL fluid in SSc-ILD patients, we recalculated the sample size for CXCL10 based on a study by Kameda et al. 2020 in which the effect sizes for comparison between CTD-ILD and IPF was 2·0. We anticipated that inclusion of 7 SSc patients in both groups would be sufficient to achieve a power of 90% at an alpha of 0·05.

Flowchart 2: Inclusion and exclusion criteria for study-2.

For Study- 1 and 2, CXCL10 ELISA on serum and BAL fluid: BAL fluid was concentrated 6x before measurement using protein concentrator with 10kDA Mwt cut-off value. Commercially available CXCL10 ELISA kit was used for both BAL fluid and serum. High performance ELISA buffer was used during serum measurements to prevent non-specific reactions. We generated a calibration curve for each plate following the manufacturer's instructions. Absorbance was read at 450nm.

Study-3: Transcriptomic (based on fibrotic gene panel of 770 genes) analysis of formalin-fixed paraffin-embedded lung tissue sections obtained from SSc-ILD lung transplant patients using NanoString nCounter Human Fibrosis panel. This is unpublished study where we use the gene of interest (cxcl10) data to further support our findings in this study on tissue level. Briefly, Twelve SSc-ILD lung sections were examined under light microscope. Inflammatory regions were defined by slight non-collagenous thickening of the alveolar walls with variable numbers of mononuclear inflammatory cells, so-called non-specific interstitial pneumonia (NSIP)-like pattern while the fibrotic regions were defined by complete destruction of normal parenchymal architecture and accumulation of collagens where alveolar walls are extremely thickened with areas of diffuse fibrosis with usual interstitial pneumonia (UIP) and fibrotic NSIP patterns.

Study-4: *In vitro* study where normal primary human lung fibroblasts were stimulated with SSc without ILD or SSc-ILD serum or BAL fluid. These fibroblasts were obtained from lung tumor resection surgery where tissues were taken as far as possible from the tumor and checked as normal by a pulmonary pathologist. Fibroblasts were cultured until confluency in a culture media (HAM F-12 + 10% FCS +1% L-Glutamine + 1% Penicillin/Streptomycin). After seeding (500,000 cells/well), cells were stimulated with 5% BAL fluid or 0·5% serum from SSc without ILD or SSc-ILD patients for 6 hours before harvesting. This experiment was replicated 3 times using one patient/group/experiment. In a different *in vitro* experiment, fibroblasts were treated with either IL-6 or TGF-β human recombinant proteins for 6 hours before harvesting. Controls were composed of wells that are either treated with complete culture media only (CTRL) or serum from healthy volunteers with no smoking, chronic illness or current use of medications (pool serum). Harvested cells were digested and total RNA was extracted for RT-qPCR using primers for genes of interest including cxcl10, ctgf, tgfβ and αsma. Gapdh was used as the housekeeping gene which the amount of target gene was normalized against it.

- 1. **Outcomes:** The primary outcome of this study was to compare CXCL10 serum levels at baseline in the SSc-ILD patients to SSc without ILD and CXCL10 at baseline association with the development of a new onset of ILD in SSc patients.
  2. **Statistical analysis:** Statistical analyses were performed using IBM SPSS Statistics version 28, R statistical software V4.3.0 and Prism GraphPad V8. Patient characteristics are presented as means with standard deviations, median with interquartile range, and counts followed by percentages, where appropriate. Mann-Whitney U tests were used for comparison between groups. Spearman’s correlation coefficients were performed to assess associations. To test for potential confounders, we performed univariate linear regressions. Confounders which were associated significantly with CXCL10 serum levels were put in multivariate linear regression model to determine their magnitude and direction. Kaplan-Meier survival curves were performed to predict ILD events and event rates were compared with Log-rank (Mantel-Cox) test. For the nanoString study, nSolver and Rosalind software were used for data and statistical analyses where P-value adjustment is performed using the Benjamini-Hochberg method of estimating false discovery rates (FDR). P-values < 0·05 were considered significant.

1. **RESULTS:**
   1. Study-1**:** Baseline serum CXCL10 levels is significantly higher in SSc-ILD patients compared to SSc without ILD [Median (IQR):126 pg/ml (66 – 282·5) vs. 78·5 pg/ml (50 – 122), *P* = 0·029, 95% CI: 1·5x10^-6^– 0·428363]. Additionally, follow-up study of SSc without ILD patients (n=124) showed that patients with CXCL10 > median levels at baseline at higher risk of developing new onset of ILD [HR= 2·74 (0·784 – 8·419), Log-rank *P* = 0·119). Serum CXCL10 levels in SSc-ILD patients were inversely correlated with %FVC predicted (r = -0·43, *P* = 0·012, 95% CI: -0·67 – 0·09).
   2. Study-2: Assaying the CXCL10 levels in BAL fluid, CXCL10 levels were higher in SSc-ILD (n=6) patients compared to patients without ILD [Median (IQR): 76·1 (7·2 – 120·8) vs 22·3 pg/ml (12·1 – 43·7), *P* = 0·24, 95% CI: -19·5 – 100). Importantly, we demonstrated that baseline serum and BAL fluid CXCL10 levels strongly correlate (*r* = 0·7, *P* = 0·007, 95% CI: 0·25 – 0·90).
   3. Study-3: transcriptomic analysis of lung tissue sections revealed that CXCL10 expression is significantly higher in inflammatory regions of SSc-ILD lung tissues compared to fibrotic regions. [Median (IQR): 4·7 (4·2-5·6) vs 4·3 (3·6-4·7), p = 0·029].
   4. Study-4: SSc-ILD biofluids (serum or BAL) induced lung fibroblasts to express significantly higher cxcl10 mRNA levels compared to biofluids from SSc without ILD or serum from healthy volunteers. Early fibrosis marker ctgf mRNA levels was significantly induced in fibroblasts treated with SSc-ILD serum compared to serum from SSc without ILD or healthy volunteers. There was no change in the expression of tgfβ or αsma mRNA levels after stimulation with SSc biofluids or healthy serum. For more details about medians, p values and point estimates, please refer to the original article.
2. **Conclusions**

Clinical, transcriptomic, and *in vitro* data showed that CXCL10 is involved in early SSc-ILD. Higher CXCL10 in SSc patients might be a potential biomarker to detect SSc patients at higher risk for the development of a new onset of ILD. Bigger cohorts are needed to confirm these observations, Moreover, future research should investigate cut-off values for CXCL10 levels in a prospective cohort to precisely predict SSc patients at high risk of developing a new onset of ILD.

2. Supp 1: Mann-Whitney U analysis, and bootstrapped results Study-1

| Test comparison | Original Mann-whitney U | Result of bootstrapped Wilcoxon |
| --- | --- | --- |
| Bootstrapping of the median between SSc-ILD and HC | W = 533, p-value = 7·021e-08 | 6·77E-08 (6·35E-08-7·17E-08) |
| Bootstrapping of the median between SSc without ILD and HC | W = 1606, p-value = 4·283e-09 | 4·88E-09 (3·25E-09-7·91E-09) |
| Bootstrapping of the median between SSc-ILD and SSc without ILD | W = 3303·5, p-value = 0·004109 | 2·90E-02 (1·48E-06-0·428363) |

Median difference in CXCL10 concentrations in serum

|  | Number of bootstrapping iterations | Median difference | 95%CI |
| --- | --- | --- | --- |
| Bootstrapping of the median between SSc-ILD and HC | 50000 | 122 | 83-216 |
| Bootstrapping of the median between SSc no ILD and HC | 50000 | 74·5 | 63·5-89·5 |
| Bootstrapping of the median between SSc-ILD and SSC no ILD | 50000 | 47·5 | 5·5-142 |


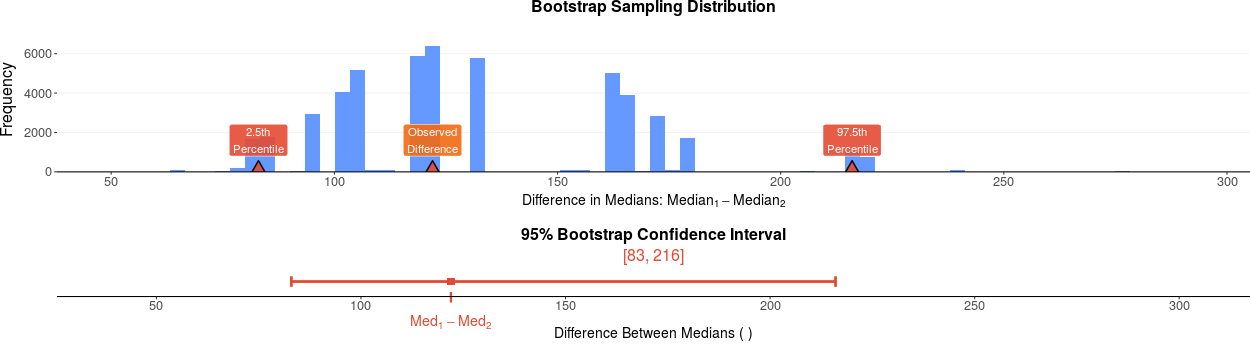

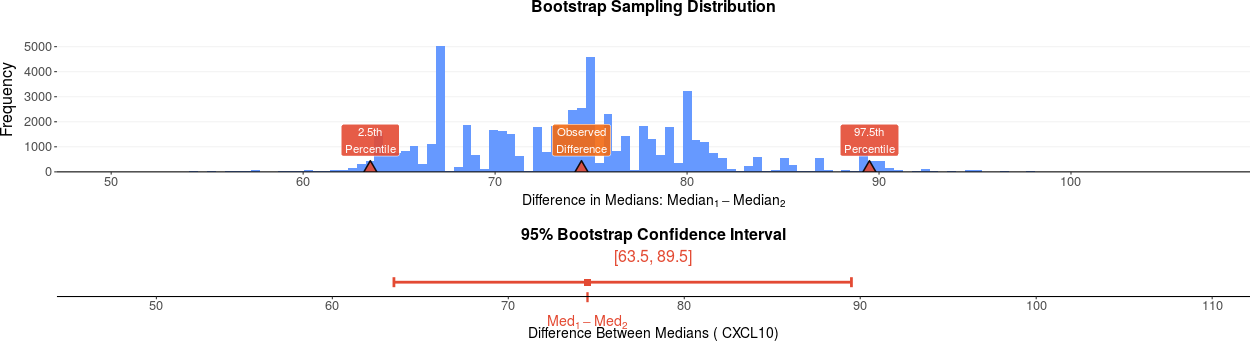


Figure 2: Median difference in SSc no ILD vs HC.

Figure 1: Median difference in SSc-ILD vs HC.


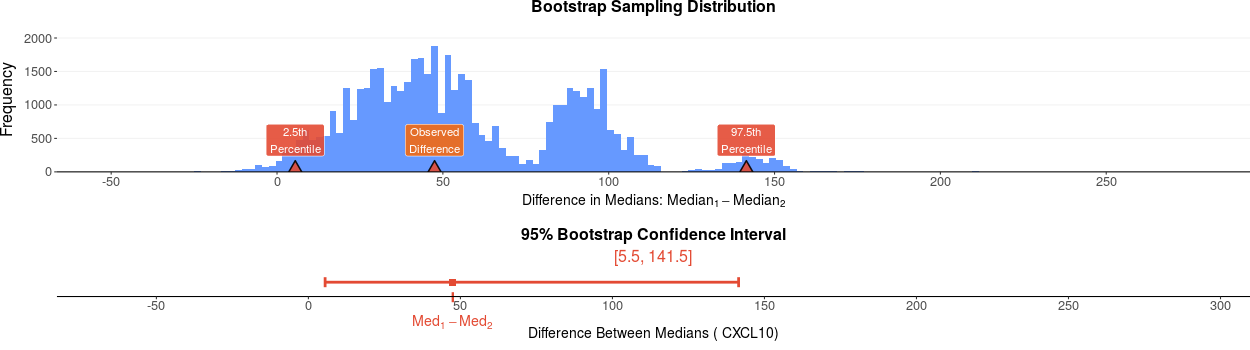


Figure 3: Median difference in SSC-ILD vs SSC no ILD.

3. Supp 2: Univariate and multivariate logistic regression between CXCL10 levels, ILD status and Important clinical variables

Univariate logistic regression

|  | OR (CI) | P |
| --- | --- | --- |
| Log(CXCL10) | 3·86 (1·56 – 9·18) | 0·004 |
| Age, in years | 1·00 (0·98 – 1·03) | 0·835 |
| Male sex, ref: female | 2·15 (0·95 – 4·78) | 0·06 |
| ACA, ref: no ACA | 0·16 (0·07 – 0·37) | <0·001 |
| DcSSc, ref: LcSSc | 2·17 (0·69 – 6·45) | 0·17 |
| Medication immunosuppression, ref: no medication | 3·63 (1·60 – 8·26) | 0·002 |

Multivariate logistic regression

|  | Full model | | Backward selected model | | |
| --- | --- | --- | --- | --- | --- |
|  | OR (CI) | P | OR (CI) | P |  |
| Log(CXCL10) | 3·03 (1·17 – 8·40) | 0·03 | 3·12 (1·24 – 8·55) | 0·02 |  |
| Age, in years | 1·01 (0·98 – 1·05) | 0·42 |  |  |  |
| Male sex, ref:female | 1·40 (0·55 – 3·48) | 0·47 |  |  |  |
| ACA, ref:no ACA | 0·15 (0·05 – 0·39) | <0·001 | 0·16 (0·06 – 0·39) | <0·001 |  |
| DcSSc, ref:LcSSc | 0·75 (0·19 – 2·68) | 0·66 |  |  |  |
| Medication immunosuppression, ref:no medication | 5·01 (1·95 –13·52) | 0·001 | 4·72 (1·85 – 12·51) | 0·001 |  |

1. Supp 3: 95% confidence intervals of regression line of %FVC and %DLco with CXCL10 at baseline in SSc-ILD group

| 95% CI of regression line %FVC and CXCL10 | -15·18 – 2·966 |
| --- | --- |
| 95% CI of regression line %DLco and CXCL10 | -16·93 – 7·044 |

univariate and multivariate linear regression between CXCL10 levels and %FVC or %DLco and important clinical variables

Individuals with ILDs, n = 34

**%FVC**

Univariate regression

|  | B | SE | P |
| --- | --- | --- | --- |
| Log (CXCL10) | -23·0 | 8·27 | 0·01 |
| Age, in years | 0·59 | 0·34 | 0·10 |
| Male sex, ref: female | -7·23 | 8·93 | 0·42 |
| ACA, ref: no ACA | 27·6 | 8·67 | 0·003 |
| DcSSc, ref: LcSSc | -28·5 | 10·8 | 0·01 |
| Medication immunosuppression, ref: no medication | 1·89 | 8·83 | 0·83 |
| COPD, ref: no COPD | 10·5 | 10·9 | 0·34 |

Multivariate regression

|  | Full model | | | Backward selected model | | |
| --- | --- | --- | --- | --- | --- | --- |
|  | B | SE | P | B | SE | P |
| Log (CXCL10) | -19·6 | 8·47 | 0·03 | -16·5 | 7·61 | 0·04 |
| Age, in years | 0·61 | 0·30 | 0·05 | 0·56 | 0·28 | 0·06 |
| Male sex, ref: female | -8·74 | 7·96 | 0·28 |  |  |  |
| ACA, ref: no ACA | 18·0 | 8·44 | 0·04 | 18·2 | 8·04 | 0·03 |
| DcSSc, ref: LcSSc | -16·3 | 11·0 | 0·15 | -17·5 | 10·2 | 0·10 |
| Medication immunosuppression, ref: no medication | -1·31 | 8·37 | 0·88 |  |  |  |
| COPD, ref: no COPD | -4·95 | 10·4 | 0·64 |  |  |  |

**%DLco predicted**

Univariate regression

|  | B | SE | P |
| --- | --- | --- | --- |
| Log(CXCL10) | -6·47 | 7·70 | 0·41 |
| Age, in years | -0·31 | 0·31 | 0·34 |
| Male sex, ref: female | -15·9 | 6·53 | 0·02 |
| ACA, ref: no ACA | -8·19 | 8·74 | 0·36 |
| DcSSc, ref: LcSSc | -9·55 | 11·7 | 0·42 |
| Medication immunosuppression, ref: no medication | 2·15 | 7·13 | 0·77 |
| COPD, ref: no COPD | -20·5 | 8·03 | 0·02 |

Multivariate regression

|  | Full model | | | Backward selected model | | |
| --- | --- | --- | --- | --- | --- | --- |
|  | B | SE | P | B | SE | P |
| Log(CXCL10) | -18·0 | 7·89 | 0·03 | -16·6 | 6·4 | 0·02 |
| Age, in years | 0·051 | 0·32 | 0·87 |  |  |  |
| Male sex, ref: female | -19·7 | 7·20 | 0·01 | -17·7 | 5·8 | 0·004 |
| ACA, ref: no ACA | -8·70 | 8·86 | 0·33 |  |  |  |
| DcSSc, ref: LcSSc | -4·04 | 11·1 | 0·72 |  |  |  |
| Medication immunosuppression, ref: no medication | -2·43 | 7·18 | 0·74 |  |  |  |
| COPD, ref: no COPD | -19·1 | 8·84 | 0·04 | -22·5 | 7·12 | 0·004 |

Individuals without ILD, n = 109

**%FVC**

Univariate regression

|  | B | SE | P |
| --- | --- | --- | --- |
| Log(CXCL10) | 2·17 | 5·02 | 0·67 |
| Age, in years | 0·47 | 0·13 | 0·001 |
| Male sex, ref: female | -9·68 | 4·55 | 0·04 |
| ACA, ref: no ACA | 5·71 | 3·68 | 0·12 |
| DcSSc, ref: LcSSc | -15·3 | 6·89 | 0·03 |
| Medication immunosuppression, ref: no medication | -4·32 | 5·04 | 0·39 |
| COPD, ref: no COPD | 12·3 | 3·03 | 0·003 |

Multivariate regression

|  | Full model | | | Backward selected model | | |
| --- | --- | --- | --- | --- | --- | --- |
|  | B | SE | P | B | SE | P |
| Log(CXCL10) | 1·02 | 5.04 | 0·84 |  |  |  |
| Age, in years | 0·35 | 0·14 | 0.015 | 0·36 | 0·14 | 0.008 |
| Male sex, ref: female | -8·87 | 4·77 | 0.066 | -9·46 | 4·45 | 0.04 |
| ACA, ref: no ACA | 0·91 | 3·79 | 0·81 |  |  |  |
| DcSSc, ref: LcSSc | -10·1 | 7·23 | 0·17 | -9·42 | 6·72 | 0·16 |
| Medication immunosuppression, ref: no medication | -2·66 | 4·91 | 0·59 |  |  |  |
| COPD, ref: no COPD | 9·27 | 4·20 | 0.03 | 9·46 | 4.08 | 0·02 |

**%DLco**

Univariate regression

|  | B | SE | P |
| --- | --- | --- | --- |
| Log(CXCL10) | -5·606 | 3·993 | 0·163 |
| Age, in years | -0·1711 | 0·1118 | 0·129 |
| Male sex, ref: female | -4·767 | 3·760 | 0·208 |
| ACA, ref: no ACA | -3·713 | 2·957 | 0·212 |
| DcSSc, ref: LcSSc | -1·295 | 5·606 | 0·818 |
| Medication immunosuppression, ref: no medication | -1·109 | 4·040 | 0·784 |
| COPD, ref: no COPD | -6·551 | 3·382 | 0·0554 |

Multivariate regression

|  | Full model | | | Backward selected model | | | |  |
| --- | --- | --- | --- | --- | --- | --- | --- | --- |
|  | B | SE | P | | B | SE | P | |
| Log(CXCL10) | -2·92 | 4·32 | 0·50 | |  |  |  | |
| Age, in years | -0·06 | 0·12 | 0·65 | |  |  |  | |
| Male sex, ref: female | -6·47 | 4·14 | 0·12 | | -6·80 | 3·89 | 0·08 | |
| ACA, ref: no ACA | -6·24 | 3·22 | 0·06 | | -6·35 | 3·06 | 0·04 | |
| DcSSc, ref: LcSSc | -0·37 | 6·18 | 0·95 | |  |  |  | |
| Medication immunosuppression, ref: no medication | 1·04 | 4·19 | 0·80 | |  |  |  | |
| COPD, ref: no COPD | -5·40 | 3·64 | 0·14 | | -6·51 | 3·34 | 0·05 | |

1. Supp Table. 1: Clinical characteristics of SSc patients who underwent *BAL procedure (study-2)*

|  | **SSc patients without ILD (n=8)** | **SSc patients with ILD (n=7)** |
| --- | --- | --- |
| Age in years, median (IQR) | 59 (52-70) | 61 (54-73) |
| Female, n (%) | 6 (75) | 4 (57·1) |
| Ethnicity, Caucasian, n (%) | 8 (100) | 7 (100) |
| Extent of skin involvement, n (%): |  |  |
| lcSSc  dcSSc  sine Scleroderma | 7 (87·5)  0 (0·0)  1 (12·5) | 5 (71·4)  0 (0·0)  2 (28·6) |
| Smoking status, n: |  |  |
| Current  Never  Ex-smoker  N/A | 2  2  3  1 | 1  3  1  2 |
| Puffy fingers or sclerodactyly, n (%) | 4 (50) | 4 (57·1) |
| Pitting scars or digital ulcers, n (%) | 3 (37·5) | 3 (42·9) |
| Telangiectasia, n (%) | 4 (50) | 3 (42·9) |
| Raynaud’s phenomenon, n (%) | 8 (100·0) | 6 (85·7) |
| Autoantibody profile, n (%): |  |  |
| Anti-centromere  Anti-topoisomerase I  Anti-RNA polymerase 3 | 4 (50)  1 (12·5)  0 (0·0) | 1 (14·3)  2 (28·6)  0 (0·0) |
| Calcinosis cutis, n (%) | 1 (12·5) | 2 (28·6) |
| Gastrointestinal involvement, n (%) | 7 (87·5) | 4 (57·1) |
| PFTs, median (IQR) |  |  |
| % FVC  % DLCO | 101 (90·5-123·5) 79·5 (71-87·5) | 93 (76·0-122·0)  66 (50-75·0) |
| COPD, n (%) | 1 (12·5) | 1 (14·3) |
| Immunosuppression  Vasodilators  Glucocorticoids | 0 (0)  7 (87·5)  1 (12·5) | 1 (14·3)  4 (57·1)  2 (28·6) |

lcSSc: limited cutaneous SSc; dcSSc: Diffuse cutaneous SSc; PFTs: pulmonary function tests; FVC: forced vital capacity; DLco: diffusion capacity for carbon monoxide; COPD: chronic obstructive lung disease.

1. Supp 4: Mann-Whitney U analysis, and bootstrapped results for Study-2

| Original Mann-whitney U | Result of bootstrapped Wilcoxon |
| --- | --- |
| W = 33·5, p-value = 0·24 | 0·56 (0·002 – 1) |

|  | Number of bootstrapping iterations | Median difference | 95% CI |
| --- | --- | --- | --- |
| Bootstrapping of the median between SSc-ILD and SSC no ILD | 50000 | 53·8 | -19·5-100 |


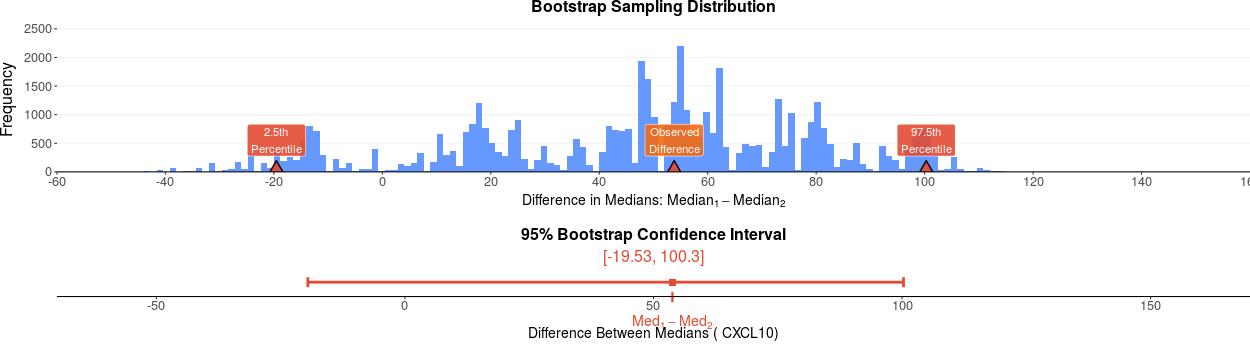


Figure 4 Median difference in BAL CXCL10 in SSC ILD vs no ILD

1. Supp Table. 2: SSc patient information of which lung tissue sections were obtained (Study-3)

|  | section name | Age | Sex | Smoking history | Lung transplant information (lung pathology) | | Date of FFPE block | | Remarks |
| --- | --- | --- | --- | --- | --- | --- | --- | --- | --- |
| Inflammatory sections (6 different sections from 6 different patients) | | | | | | | |  |  |
| 1 | A I 1 | 44 | F | former smoker; for 30 years | Bilateral pneumoectomy, NSIP with diffusing fibrosis in some regions. | | 2017 | |  |
| 2 | E II 7 | 56 | M | ND | Bilateral pneumoectomy, variable intertitial fibrosis and vascular pathology (PAH) | | 2003 | |  |
| 3 | H II 7 | 36 | M | Never | Bilateral pneumectomy, variable staging of fibrosis (diffuse and NSIP-like), PAH is also present | | 2018 | |  |
| 4 | K I 4 | 47 | F | Never | Bilateral lung transplant (section is for the right lung), GGO of NSIP pattern, arteriopathy linked with PAH | | 2019 | |  |
| 5 | G 1 | 57 | M | ND | Bilateral pneumoectomy, pleural and interstitial fibrosis, there is also severe vasculopathy | | 2018 | | excluded by QC nanoSring |
| 6 | U II 2 | 60 | M | ND | Bilateral transplant, severe diffuse interstitial fibrosis, late phase fibrosis, vasculopathy is hard to be detected | | 2011 | |  |
| Fibrotic sections (6 different sections from 6 different patients) | | | | | |  |  |  |  |
| 1 | F II 5 | 38 | M | ND | Bilateral pneumoectomy, severe vascular pathology, some of the fibrosis is consistent with PAH | | 2005 | |  |
| 2 | G I 7 | 57 | M | ND | Bilateral pneumoectomy, pleural and interstitial fibrosis, there is also severe vasculopathy | | 2018 | |  |
| 3 | H I 6 | 36 | M | Never | Bilateral pneumectomy, variable staging of fibrosis (diffuse and NSIP-like), PAH is also present | | 2018 | |  |
| 4 | R II 6 | 34 | F | ND | Bilateral explants, NSIP phenotype | | 2007 | |  |
| 5 | S II 8 | 55 | F | Never | Bilateral transplantation, bronchictases(extensive) and active inflammation, vasculopathy is present (arterial) | | 2010 | |  |
| 6 | W I 6 | 62 | M | ND | right unilateral transplant, variable picture of the lung; NSIP-like severe fibrosis progressing into advanced fibrosis with no specific pattern | | 2013 | |  |

1. Supp Table. 3: Clinical information regarding patients of whom biomaterials were obtained (Study-4)

|  | SSc-without ILD (N=3) | SSc-ILD (N=3) |
| --- | --- | --- |
| Age in years, Median (IQR) | 65 (62 – 71) | 55 (42 – 78) |
| Sex, female | 2 | 1 |
| Extent of skin involvement, n  lcSSc  dcSSc  Sine | 3 | 2  1 |
| Smoking history, n  Current  Never  Ex-smoker  N/A | 2  1 | 1  1  1 |
| Puffy finger or Sclerodactyly | 1 | 3 |
| Pitting scars or digital ulcer | 1 | 2 |
| Telangiectasia | 2 | 1 |
| Raynaud’s Phenomenon | 3 | 2 |
| Autoantibody profile  Anti-centromere  Anti-topoisomerase I  Anti- RNA polymerase 3  other | 1  2 | 3 |
| Calcinosis cutis | 0 | 2 |
