# Supplementary material for: High serum C-X-C motif chemokine ligand 10 (CXCL10) levels may be associated with new onset interstitial lung disease in patients with systemic sclerosis: evidence from observational, clinical, transcriptomic and in vitro studies
Source: eBioMedicine. 2023 Nov 22;98:104883. doi: 10.1016/j.ebiom.2023.104883 (PMC10708993; doi:10.1016/j.ebiom.2023.104883)
Supplement: Protocol study 1 Retrospective cohort Non-WMO [file mmc2.pdf]

Template Non-WMO research protocol

University Medical Center Groningen

ORGANIZATION

**Protocol ID**	**Study number: 201900260**
**Name**	A retrospective study for finding new biomarkers for the prediction and early detection of prognosis and early organ involvement in patients with systemic sclerosis.
**Title**	A retrospective study for finding new biomarkers for the prediction and early detection of prognosis and early organ involvement in patients with systemic sclerosis.
**Version**	**1**
**Date of submission**	**15-04-2019**
**Anticipated start and end date of project**	Starting date: 01-05-2019<br>End date: 01-05-2024
**Coordinating investigator /Project leader (name, affiliation)**	**Dr. D.J. Mulder**<br>Dept. Internal Medicine, div. Vascular Medicine Internal code AA41, University Medical Center Groningen, PO box 30.001, 9700 RB Groningen, The Netherlands<br><br>**Ms. I.M. Atzeni**<br>Dept. Internal Medicine, div. Vascular Medicine, Internal code AA41, University Medical Center Groningen, PO box 30.001, 9700 RB Groningen, The Netherlands.<br>Phone: +31 50 361 0116
**(Principal) investigator(s)**	**Dr. D.J. Mulder (PI)**<br>Dept. Internal Medicine, div. Vascular Medicine Internal code AA41, University Medical Center Groningen, PO box 30.001, 9700 RB Groningen, The Netherlands.<br><br>**Dr. J. Westra (co-PI)**<br>Lab Immunology<br>Dept. Rheumatology and Clinical Immunology<br>University Medical Center Groningen, PO box 30.001, 9700 RB Groningen, The Netherlands.<br><br>**Dr. C. Roozendaal (co-PI)**<br>Dept. Laboratory Medicine<br>University Medical Center Groningen, PO box 30.001, 9700 RB Groningen, The Netherlands.
**Sponsor (in Dutch:**	University Medical Center Groningen

**verrichter/opdrachtgever)**	PO Box 30.001<br>9700 RB Groningen<br>The Netherlands
**Subsidising party**	***N/A***
**Laboratory / research sites**	Lab Immunology<br>Dept. Rheumatology and Clinical Immunology<br>University Medical Center Groningen, PO box 30.001, 9700 RB Groningen, The Netherlands.<br>Phone: +31 50 361 3400

umcg

PROTOCOL SIGNATURE SHEET

Name	Signature	Date
**Principal Investigator:**<br>*D.J. Mulder*	[signature]	15/4/19

SUMMARY OF RESEARCH

Rationale: No biomarkers are available for early detection and follow-up of systemic sclerosis (SSc). Several cytokines and chemokines have been suggested in literature, but no biomarker has reached the stage of clinical applicability. Interleukin-6 (IL-6) is of special interest, as it may play an important role in the early development of interstitial lung disease (ILD) in SSc patients. Moreover, new pathogenetic mechanisms should be explored. Our research group has demonstrated that soluble RAGE (sRAGE) and high mobility group box 1 (HMGB1) are elevated in sera of SSc patients, and may serve as potential targets for early treatment. However, the clinical spectrum, the association with organ complications and disease stage and severity, and prognostic value of these new biomarkers should be elucidated.

Research question: The aim of the study is to investigate the prognostic value of novel serum biomarkers, in patients with SSc and their association with early organ complications and disease stage, severity and progression.

Study design: This study will retrospectively and sequentially assess blood samples of SSc and Raynaud's phenomenon (RP) patients. Readily available serum will be used for measurements.

Study population: 417 SSc and RP patients in whom blood was drawn for clinical purposes.

Determinants / intervention: Biomarkers in serum of 417 SSc and RP patients that has been stored for clinical practice will be retrospectively assessed.

Duration and main study outcomes: The main study outcomes are levels of selected biomarkers, such as CRP, ESR, IL-6, CXCL4, CXCL10, CCL18, thiols, sRAGE and HMGB1, their prognostic value, their association with organ involvement, disease stage, severity and progression in patients with SSc. The anticipated end date of the study is 01-05-2024.

1. RESEARCH

1.1 INTRODUCTION AND RATIONALE

Systemic sclerosis (SSc) is a progressive fibro-inflammatory autoimmune disease of which the exact trigger that initiates disease is unknown. Raynaud's phenomenon (RP), vasospastic attacks of the digital arteries, are usually the first symptom. Due to underlying fibrotic processes irreversible skin and organ involvement insidiously evolves. Patients often approach a clinician when a lot of irreversible damage has already taken place. It is, therefore, of importance to detect the disease early and treat it at an early stage to improve its prognosis. Nowadays, there are just a few diagnostic tools for patients with SSc, serum biomarkers may be a cheap and easy new solution.

Interstitial lung disease (ILD) is the main cause of death in SSc and associated with large fibroproliferative changes. Studies showed that interleukin-6 (IL-6) plays an important role in early phases, characterized by local inflammation, of ILD in SSc patients.

Furthermore, recent studies have shown that soluble RAGE (sRAGE) and HMGB1 are elevated in sera of SSc patients. Advanced glycation endproducts (AGEs) are oxidative stress derived compounds with potential proinflammatory effects. The receptor for AGEs is RAGE, which is also the receptor for high mobility group box 1 (HMGB1), a nuclear protein, which is proinflammatory when released from activated or apoptotic cells. Their exact role in SSc is yet not studied. We have recently found that *in vitro* stimulation of fibroblasts with AGE-BSA and HMGB1 leads to high production of IL-6.

Therefore, the aim of our study is to investigate the role of several cytokines and chemokines in the pathogenesis of SSc and their role in early disease detection.

1.2 What are the research objectives / question(s) of the proposed research? Distinguish a primary objective and secondary objectives if applicable.

Please specify the main and secondary objectives. The primary objective is the main question that determines study design and sample size. Max 50 words.

The aim of the study is to investigate the prognostic value of novel serum biomarkers, in patients with SSc and their association with early organ complications and disease stage, severity and progression. Relation to other clinical measures will be also investigated.

2. STUDY DESIGN

2.1. For intervention studies describe intervention(s) and co-interventions (if applicable) and control group. *Max 50 words.*

N/A

2.2. Short description of the duration and method of the study. *Max 50 words.*

This retrospective study is set up as a research program in which we will retrospectively asses data of SSc and RP patients. Sera of patients from 2013-2018 has already been taken and stored for clinical purposes. These sera will be thrown away after 5 years from the time of collection. This serum will be used for the analysis of the biomarkers for this study. Blood from SSc and RP patients will be retrospectively assessed. Blood was already drawn and stored for clinical purposes. We will measure levels of biomarkers, including IL-6, CXCL4, CXCL10, CCL18, thiols and sRAGE in these blood samples with in-house developed enzyme-linked immunosorbent assays (ELISAs). Levels of HMGB1 will be quantified by a commercial ELISA kit, according to manufacturer's instructions. Organ manifestations will be documented from medical dossiers. CRP and ESR has mostly already been measured for clinical purposes.

2.3. Define the research population, please make clear why this study should be done in this selected population.

From what source population will the subjects be drawn (what are population restrictions inherent to that data source)? Is the cohort defined at a fixed point in time or dynamic (depending on an event at subject level)? Max 50 words.

All 199 SSc and 218 RP patients from the UMCG will be included.

2.4. Inclusion- and exclusion criteria.

Inclusion criteria:

- 18 years or older
- Patients with SSc and RP

Exclusion criteria:

- Patients who objected to participate in research (Verification objection registry)

2.5. Justification for the sample size.

If applicable conduct the power analysis for your primary research question and outcome or explain how many participants will be enrolled and how this number was determined. Max 50 words.

Sample size (power 80%, alpha 0.05) is based on previous studies in ILD, with an effect size of 0.4. Therefore 100: 100, SSc: RP should be sufficient. To correct for invalid samples 417 patients will be included. We expect a low effect size due to many potential confounders. Moreover, as mentioned above, sera have already been taken for clinical purposes and stored and will be thrown away after 5 years.

2.6. The participants are:

Please choose **all** that apply:

☐ Younger than 12 years old
☐ Between the age of 12 and 16 years old
☒ 16 years or older
☒ Legally capable (i.e., able to sign informed consent)

Participation of minors and/or people with incapacities is only accepted if the proposed research cannot be performed with adults and people with capacities as participants (see article 4 WMO).

2.7. Will the participants (and/or parents/guardians if the participant is under age 16 and/or incapable of signing the informed consent) be informed about the nature, objectives, methods and risks of the study before they participate in the study?

☐ Yes - if yes, in what way?
☒ No - if no provide a brief explanation below.

Given the retrospective nature of this study, the large amount of patients which are regarded eligible for inclusion, and the low risk associated with this study, we believe that obtaining written informed consent forms and sending out patient information is not achievable. Moreover, some patients visited our department only once, and, therefore, it remains unknown whether patients have moved and whether these patients are still alive. We believe

that by contacting these patients or family members of these patients we would only increase the burden. However, any patient who has specifically objected to participate in research of any kind (Verification objection registry) will be excluded. Moreover, in 2018 we have set-up a Raynaud-lines databank, in which we ask patients visiting our center for Raynaud and SSc for their consent to use their data for research upfront. We are working on expanding this databank to a biobank, allowing a full informed consent procedure for all potential participants in the future.

2.8. Will the participants (or their parents/guardians if the participant is younger than 16 years old and/or unable to sign the informed consent) sign an informed consent prior to the study?

☐ Yes
☒ No - if no provide a brief explanation below.

Please refer to 2.7.

2.9. Will the participants be informed about the nature and purpose of the performed research after it is completed?

☐ Yes - if yes, in what way?
☒ No - if no, provide a brief explanation.

Please refer to 2.7.

2.10. Do the participants receive any reward for their participation?

☐ Yes - if yes, specify below the nature and extent of the reward.
☒ No

2.11. Will the participants be informed prior to the experiment that they can withdraw from the study at any time?

☐ Yes
☒ No - if no provide a brief explanation below.

Please refer to 2.7.

2.12. Describe the recruitment and informed consent procedures.

Describe how and by whom (investigator, supervising doctor, other person) will subjects inform about the study and ask for their consent and how much time is given for the decision to participate or not. Max 50 words.

This is a retrospective study, in which all measurement and information on patient characteristics were obtained between 2013 and 2018. Please refer to 2.7 for information about recruitment and informed consent procedures.

MAIN OUTCOMES / ENDPOINTS OR CASE DEFINITIONS

2.13. Main study parameters/ endpoints/ outcomes

Specify your dependent (outcome) measures and independent measures.

- IL-6 levels in serum and organ involvement in patients with SSc and RP.

2.14. Other (secondary) parameters, covariates, confounders

- Patient characteristics: gender, age, co-morbidities and use of medication.
- Disease characteristics: parameters on SSc organ and skin involvement, SSc-naitbody profile, disease duration, iloprost/prostacyclin use (indication, frequency and dosage), presence of an underlying connective tissue disorder, positive family history for SSc or other vascular diseases.
- Serum levels of inflammatory and fibroproliferative cytokines, including CRP, ESR, CXCL4, CXCL10 and CCL18.
- Serum levels of the following biomarkers, including HMGB1, thiols and sRAGE.

2.15. Please give an adequate description of the procedures, instruments, methods and/or tests to be used to assess all defined study parameters/endpoints

This question also involves information about the equipment/ recording devices, and other measurement instruments (e.g. questionnaires) that will be used in the research.

Demographic variables will be obtained from medical dossiers. Clinical characteristics will be collected retrospectively. These consist of age, gender, disease duration, laboratory (e.g., serological tests) and, functional test (e.g., pulmonary function tests, esophageal scintigraphy, high-resolution CT and cardiac ultrasound).

3. DATA ANALYSIS

3.1. Describe for each of the parameters (as mentioned in 4), in general terms, how the data will be presented (quantitative and/or qualitative), and how derived parameters will be calculated (if applicable). *Max 100 words.*

All parameters will be presented as quantitative data.

3.2. How will missing data be handled?
This includes an estimation of initial non-response; drop-out rate; missing scores. Max 50 words.

Only patients in which serology test were done will be included in the current study.

3.3. How will the data be statistically analysed?

The normal distribution will be tested with a Q-Q plot. Non-parametric tests (X^2, Mann-Whitney U or Kruskal Wallis) will be used in case of non-normally distributed data to determine group differences. Correlations between variables will be determined by either Pearson or Spearman correlation coefficients, depending on the normal distribution. Also, linear regression analysis will be performed to correct for confounders. A p-value below 0.05 will be considered significant.

4. DATA STORAGE

4.1. Is a Data Management Plan (DMP) available?

- ☒ Yes, if yes please add to the application
- ☐ No - if no describe below how (personal) data will be handled in relation to Dutch regulation. Which steps are taken to ensure data security and how the participant's privacy is protected?

4.2. Will the personal information of the participants be coded in the proposed research?

- ☒ Yes - if yes, who owns the encryption key?
- ☐ No - if no, describe below how the security is warranted?

D.J. Mulder and I (Isabella Atzeni) will own the encryption key.

4.3. Who has access to the source documents and any other personally identifiable data? *Max 50 words.*

Only the investigators involved in this research have access to the source documents and other personally identifiable data.

4.4. Are the participants allowed to view their own data upon request?

- ☒ Yes
- ☐ No - if no', provide a brief explanation below.

4.5. Does the research involve capturing video and/or audio data?

- ☐ Yes
- ☒ No

4.6. If yes, will these video and/or audio files be saved with an encryption in a protected folder?

N/A

5. Load and Risk

5.1. Provide a brief but specific description of the nature, duration and intensity of the physical, psychological and mental strain that participants will experience during the research. *Max 100 words.*

N/A, given the retrospective nature of this study.

5.2. Will the participants risk any injuries and/or other discomfort when they participate in the proposed research?

- ☒ Minimal risk - Minimal risk means that the chance of physical/psychological stress and discomfort is not higher than the chance of injuries/discomfort in daily life.
- ☐ More than minimal risk - provide a brief explanation below.

5.3. Do the researchers take specific precautions to prevent participants from injuries / discomfort?

- ☐ Yes – if yes, provide a brief explanation below
- ☒ No
