# Supplementary material for: High serum C-X-C motif chemokine ligand 10 (CXCL10) levels may be associated with new onset interstitial lung disease in patients with systemic sclerosis: evidence from observational, clinical, transcriptomic and in vitro studies
Source: eBioMedicine. 2023 Nov 22;98:104883. doi: 10.1016/j.ebiom.2023.104883 (PMC10708993; doi:10.1016/j.ebiom.2023.104883)
Supplement: Protocol appendix study 2&4 [file mmc3.pdf]

Assessing the role of local inflammatory, fibroproliferative, and osteogenic biomarkers in Systemic Sclerosis related Interstitial Lung Disease.

ILD-SSc study

PROTOCOL TITLE 'Assessing the role of local inflammatory, fibroproliferative, and osteogenic biomarkers in Systemic Sclerosis related Interstitial Lung Disease'.

**Protocol ID**	**SSc-ILD study (METc 2020.276)**
**Short title**	Assessing the role of local inflammatory, fibroproliferative, and osteogenic biomarkers in Systemic Sclerosis related Interstitial Lung Disease
**EudraCT number**	n/a
**Version**	~~2~~3
**Date**	~~30-06-2020~~24-07-2020
**Principal investigator**	*Dr. D.J. Mulder*<br>Dept. Internal Medicine, div. Vascular Medicine, Internal code AA41, University Medical Center Groningen, PO Box 30.001, 9700 RB Groningen, The Netherlands.<br>Phone: +31 50 361 2350<br>Fax: +31 50 361 3312
**Co-investigators**	*Ms. I.M. Atzeni*<br>Dept. Internal Medicine, div. Vascular Medicine, Internal code AA41, University Medical Center Groningen, PO box 30.001, 9700 RB Groningen, The Netherlands.<br>*Drs. Y. Al-Adwi Mohammed*<br>Dept. Internal Medicine, div. Vascular Medicine, Internal code AA41, University Medical Center Groningen, PO box 30.001, 9700 RB Groningen, The Netherlands.<br>*Dr. J. Westra*<br>Lab Immunology<br>Dept. Rheumatology and Clinical Immunology

	University Medical Center Groningen, PO box 30.001, 9700 RB Groningen, The Netherlands.<br>Phone: +31 50 361 3400<br><br>*Prof H. van Goor*<br>Lab pathology<br>University Medical Center Groningen, PO box 30.001, 9700 RB Groningen, The Netherlands.<br>Phone: +31 50 361 3400<br><br>*Dr. ir. A. van Roon*<br>Vascular Laboratory Internal Medicine<br>Dept. Internal Medicine, div. Vascular Medicine, University Medical Center Groningen, PO box 30.001, 9700 RB Groningen, The Netherlands.<br>Phone: +31 50 361 2097<br><br>*Dr CT Gan, MD, PhD, pulmonologist*, dept. Pulmonology, University Medical Center Groningen, University of Groningen, Groningen, The Netherlands.
**Sponsor (in Dutch: verrichter/opdrachtgever)**	*University Medical Center Groningen*<br><br>PO box 30.001, 9700 RB Groningen,The Netherlands.
**Subsidising party**	Sanofi Genzyme, 50 Binney Street, Cambridge, MA 02142, USA
**Independent expert (s)**	*Dr. E.J. Houwerzijl*<br>Dept. Internal Medicine, div. Vascular Medicine, University Medical Center Groningen,<br>PO box Box 30.001, 9700 RB Groningen, The Netherlands.<br>Phone: +31 50 361 0550

	E-mail: e.j.houwerzijl@umcg.nl
**Laboratory sites**	Lab Immunology<br>Dept. Rheumatology and Clinical Immunology<br>University Medical Center Groningen, PO box 30.001, 9700 RB Groningen, The Netherlands.<br>Phone: +31 50 361 3400<br><br>Lab Pathology<br>University Medical Center Groningen, PO box 30.001, 9700 RB Groningen, The Netherlands.<br>Phone: +31 50 361 3400
**Vascular Lab**	Vascular Laboratory Internal Medicine<br>Dept. Internal Medicine, div. Vascular Medicine, University Medical Center Groningen, PO box 30.001, 9700 RB Groningen, The Netherlands<br>Phone: +31 50 361 5949
**Pharmacy**	NA

PROTOCOL SIGNATURE SHEET

Name	Signature	Date
**Head of Department:** **Prof Dr ROB Gans**		
**Principal Investigator:** **Dr DJ Mulder**		~~30-06-2020~~24-07-2020

TABLE OF CONTENTS

SUMMARY 9
1 INTRODUCTION AND RATIONALE 12
2 OBJECTIVES 16
3 STUDY DESIGN 17
3.1 Design: 17
3.2 Duration and setting: 17
3.3 Schedule of visits: 17
4 STUDY POPULATION 18
4.1 Population (base) 18
4.2 Inclusion criteria 18
4.3 Exclusion criteria 19
4.4 Sample size calculation 20
5 TREATMENT OF SUBJECTS 21
6 NON-INVESTIGATIONAL PRODUCT 22
7 METHODS 23
7.1 Study parameters/endpoints 23
7.1.1 Main study parameter/endpoint 23
7.1.2 Secondary study parameters/endpoints 23
7.2 Randomisation, blinding and treatment allocation 24
7.3 Study procedures 24
7.3.1 Routine clinical assessments from which data will be used in the current study. 24
7.3.2 Study procedures ..... 26~~25~~
7.4 Withdrawal of individual subjects ..... 29~~28~~
7.4.1 Specific criteria for withdrawal ..... 29~~28~~
7.5 Replacement of individual subjects after withdrawal ..... 29~~28~~
7.6 Follow-up of subjects withdrawn from treatment ..... 29~~28~~
7.7 Premature termination of the study 29
8 SAFETY REPORTING 30
8.1 Temporary halt for reasons of subject safety 30
8.2 AEs, SAEs and SUSARs 30
8.2.1 Adverse events (AEs) 30
8.2.2 Serious adverse events (SAEs) 30
8.2.3 Suspected unexpected serious adverse reactions (SUSARs) 31
8.3 Annual safety report 32
8.4 Follow-up of adverse events 32
8.5 [Data Safety Monitoring Board (DSMB) / Safety Committee] 32
9 STATISTICAL ANALYSIS 33
9.1 Primary study parameter(s) 33
9.2 Secondary study parameter(s) 33
9.3 Other study parameters 33

9.4 Interim analysis 33
10 ETHICAL CONSIDERATIONS 34
10.1 Regulation statement 34
10.2 Recruitment and consent 34
10.3 Objection by minors or incapacitated subjects 34
10.4 Benefits and risks assessment, group relatedness 34
10.5 Compensation for injury 35
10.6 Incentives 35
11 ADMINISTRATIVE ASPECTS, MONITORING AND PUBLICATION .... 37~~36~~
11.1 Handling and storage of data and documents .... 37~~36~~
11.2 Monitoring and Quality Assurance .... 37~~36~~
11.3 Amendments .... 37~~36~~
11.4 Annual progress report .... 38~~37~~
11.5 Temporary halt and (prematurely) end of study report .... 38~~37~~
11.6 Public disclosure and publication policy .... 38~~37~~
11.7 STRUCTURED RISK ANALYSIS .... 39~~38~~
12 REFERENCES .... 41~~39~~

LIST OF ABBREVIATIONS AND RELEVANT DEFINITIONS

ABR	ABR form, General Assessment and Registration form, is the application form that is required for submission to the accredited Ethics Committee (In Dutch, ABR = Algemene Beoordeling en Registratie)
AE	Adverse Event
AR	Adverse Reaction
CA	Competent Authority
CCMO	Central Committee on Research Involving Human Subjects; in Dutch: Centrale Commissie Mensgebonden Onderzoek
CV	Curriculum Vitae
DSMB	Data Safety Monitoring Board
EU	European Union
EudraCT	European drug regulatory affairs Clinical Trials
GCP	Good Clinical Practice
IB	Investigator's Brochure
IC	Informed Consent
IMP	Investigational Medicinal Product
IMPD	Investigational Medicinal Product Dossier
METC	Medical research ethics committee (MREC); in Dutch: medisch ethische toetsing commissie (METC)
RP	Raynaud's phenomenon
(S)AE	(Serious) Adverse Event
SPC	Summary of Product Characteristics (in Dutch: officiële productinfomatie IB1-tekst)
Sponsor	The sponsor is the party that commissions the organisation or performance of the research, for example a pharmaceutical company, academic hospital, scientific organisation or investigator. A party that provides funding for a study but does not commission it is not regarded as the sponsor, but referred to as a subsidising party
SUSAR	Suspected Unexpected Serious Adverse Reaction
SSc	Systemic Sclerosis
Wbp	Personal Data Protection Act (in Dutch: Wet Bescherming Persoonsgevens)
WMO	Medical Research Involving Human Subjects Act (in Dutch: Wet Medisch-wetenschappelijk Onderzoek met Mensen

SUMMARY

Rationale:

Systemic sclerosis (SSc) is a rare progressive autoimmune disease hallmarked by severe vasculopathy, leading to skin and internal organ complications and premature mortality. A considerable proportion of SSc patients develop Systemic Sclerosis related Interstitial Lung Disease (SSc-ILD), for which treatment options are limited. Although extensive SSc-ILD has been shown to be associated with large fibroproliferative changes, early phases of limited SSc-ILD seem to be characterized by local inflammation. Pre-clinical and early clinical studies indicate that interleukin-6 may play a central role during the early phase of SSc-ILD. Additionally, patients with SSc are prone to enhanced calcification of skin (calcinosis cutis) and vasculature. This calcification process is strongly associated with local inflammation in the skin, which is a process that may very well also occur in internal organs and serve as an early proxy for long-term SSc-related complications. In fact, in patients with SSc, diffuse pulmonary ossifications are frequently observed, which may indicate a common pathway of systemic inflammation, fibrosis, and calcification in interstitial lung disease (ILD) in SSc.

Hypothesis:

Assessment of the proinflammatory, profibrotic and osteogenic profile of the lungs in patients with SSc could serve as early markers of disease, and could potentially facilitate upfront treatment in patients with limited SSc-ILD.

Objectives:

1. To compare the proinflammatory, profibrotic and osteogenic cytokine profile in BAL fluid between SSc patients without ILD, with limited ILD, and with extensive ILD.
2. To compare the proinflammatory, profibrotic and osteogenic differentiation of fibroblasts isolated from lung BAL fluid and bronchial biopsies between SSc patients without ILD, with limited ILD, and with extensive ILD.
3. To compare the expression of markers involved in the calcification process in bronchial biopsies assessed by immunohistochemistry between SSc patients without ILD, with limited ILD, and with extensive ILD.
4. To assess the association of diffuse pulmonary ossifications on HRCT with the cytokine profile in BAL fluid, the fibroblast differentiation in lung fibroblasts, and calcification process in lung tissue in patients with SSc

5. To assess the cytokine profile in BAL fluid, the fibroblast differentiation in lung fibroblasts, and calcification process in lung tissue with systemic factors, including serum markers of inflammation, fibrosis, and the calcification process, non-invasive markers of micro- and macrovasculopathy, and the extent of skin and other internal organ complications in patients with SSc.

Study design:
This is an explorative substudy of the CALC-SSc study (NL65651.042.18, METC 2018.373), with a *case-control* and *prospective longitudinal* design. It largely follows its hypothesis and studies the same patient population base as this study.

Study population:
Participants will be 18-70 years old. They will have to give written informed consent prior to study activities. SSc will be classified based on 2013 ACR/EULAR criteria, yielding the following groups:

- 10 SSc-ILD patients with limited lung disease (*"limited SSc-ILD*"), not qualifying for prompt treatment.
- 5 SSc-ILD patients with extensive lung disease ("*extensive SSc-ILD"*), before initiation of first-line systemic therapy.
- 5 SSc patients without SSc-ILD ("*SSc without ILD")*.

Intervention:
Not applicable (purely observational).

Main study parameters/endpoints:

1. Level of IL-6 in BAL fluid
2. Inflammatory, fibroproliferative and osteogenic cytokines in BAL fluids
3. Degree of proinflammatory, profibrotic and osteogenic differentiation of fibroblasts isolated from lung BAL and bronchial biopsies
4. Degree of tissue markers involved in the calcification process and SSc progression in bronchial biopsies
5. Degree of other serum markers of the calcification process: T50, calcium, phosphorus, parathormone, fetuin-A, Fibroblast Growth Factor-23 (FGF23), αKlotho;
6. Degree and extent of pulmonary involvement of ILD and pulmonary ossifications by lung function (FVC, FEV1, DLCO), and HRCT

7. Degree of vasculopathy by: noninvasive nailfold capillary microscopy; arterial stiffness; digital artery involvement as assessed with ultrasound;
8. Clinical parameters on SSc organ and degree of skin and additional organ involvement, SSc-antibody profile, medication use, and comorbidities.

Nature and extent of the burden and risks associated with participation, benefit and group relatedness:

Attempts will be made to have patients visit our center only once for assessment of all study parameters. Blood drawing will be combined with routine clinical practice assessments to minimize the venipuncture burden. Bronchoscopy may result in minor discomfort (not needing medical attention) in <10%, and in <1% it may result in more severe complications (pneumonia and bleeding) needing medical attention and intervention. By excluding participants using anticoagulants or anti-platelet drugs, the risk of bleeding is limited as much as possible.

1 INTRODUCTION AND RATIONALE

Systemic sclerosis (SSc) is a rare progressive connective tissue autoimmune disease with a major burden of morbidity and mortality (1). Unfortunately, treatment options are limited and disease course is often insidious (2). Cold-induced digital artery spasms known as Raynaud's phenomenon (RP) are usually the first presenting symptom. Although RP occurs in the general population as harmless discomfort, it may progress in SSc patients to serious ischemic complications (3,4). Thereafter, some patients develop "non-Raynaud" symptoms such as skin fibrosis (scleroderma), gastrointestinal dysmotility, renal crisis, and severe and potentially lethal lung involvement such as interstitial lung disease (ILD) and pulmonary arterial hypertension (PAH) (5,6). Although several treatment options are currently applied in patients with end-organ involvement, no curative, disease-modifying therapy is available. This stresses the need for early detection of the disease activity allowing early interventions.

Although the etiology of SSc is currently incompletely understood, it is generally agreed that it is an autoimmune disease, with low-grade inflammation being an early step in the disease course (7). In addition to RP, patients with early signs of SSc generally have SSc specific antibodies and distinct abnormalities of the capillaries, which can be clearly visualized using nail-fold capillary microscopy. Furthermore, many patients present so-called "puffy fingers" which are the result of interstitial edema following low-grade inflammation and endothelial damage (8). In later stages of the disease, this process will progress to fibrosis as a result of myofibroblast activation and abundant collagen deposition in the extracellular matrix in skin, vasculature and internal organs, hallmarking the disease as "systemic sclerosis". During the phase of low-grade inflammation, it might be possible to initiate potential disease-modifying therapies. However, these interventions often consist of immune-modulatory therapies associated with a considerable burden of opportunistic infections and other complications. Therefore, there is a need to better characterize patients at risk and to identify organ involvement at an early stage.

Calcinosis cutis is a difficult to treat complication of SSc, which is characterized by prominent skin calcifications arising without an apparent disorder in calcium-phosphate metabolism (9). Importantly, these patients are prone to severe disease and an exaggerated vasculopathy indicated by more telangiectasia, giant capillaries and a reduced capillary density than those without calcinosis cutis (9–11). This active calcification process not only occurs at sites recognized as calcinosis cutis but can also be detected at skin sites without overt calcification (12). At these sites, overexpression of calcification related matrix proteins such as osteonectin and matrix gammacarboxyglutamic acid protein (MGP) have been

observed. Both of these proteins play an important role in the metabolism of calcification and are commonly found in bony tissue. Osteonectin was not only overexpressed in the dermal matrix, but also in fibroblasts and endothelial cells. Although these observations were more prominent in SSc patients with overt calcinosis cutis, the fact that similar mechanisms seem to play a role subclinically in those without overt cutaneous calcifications suggests that calcification may be an ubiquitous process in SSc. Additionally, αKlotho (Kl) which is a crucial regulator of calcium homeostasis, is significantly decreased in the microvasculature of SSc skin (13). Also, circulating αKlotho levels were shown to be decreased (14). No association was found with the severity of organ involvement, which may reflect the poor representability of circulating αKlotho measurement. Importantly, soluble Kl administration may effectively improve microvascular endothelial cells from SSc patients in vitro by acting as a powerful proangiogenic factor (15).

Many SSc patients will suffer from cardiovascular disease (17). In patients with SSc, increased “stable” calcification has been clearly found in the arteries. For example, coronary calcification assessed by coronary CT seems to be present. Another study showed that signs of coronary calcification by coronary CT were present in 56.2% of SSc patients and in only 18.8% of age-, sex-, and race-matched controls (18). Also, intracerebral vascular calcifications, an independent risk factor of ischemic stroke in the general population, were found by non-contrast CT scan in 32% of asymptomatic SSc patients but in only 9% of controls (19). In line with the above, serum levels of fetuin-A, a natural calcification-inhibitor, are decreased in SSc, irrespective of calcinosis cutis, and are strongly associated with vasculopathy (20,21). Fetuin-A also has antifibrotic characteristics, participates in tissue remodeling, and is negatively associated with endothelin-1 (ET-1), which is a key marker involved in SSc vasculopathy (20,22). Additionally, recent studies have demonstrated that patients with SSc have a specific interferon-1 (IFN-1) signature that can be assessed in the peripheral blood as well as in skin (23). Furthermore, it has been shown that the angiogenic repair is hampered, partly by a decreased recruitment of angiogenic cells from the bone marrow. These include endothelial progenitor cells and angiogenic T cells and putatively an increase in angiopoietin 2 and TIE2 positive monocytes (24). These pathways may interact with the calcification process, promoting inflammation, calcification, and ultimately fibrosis.

A potential switch may be through RAGE, Toll-like receptor (TLR) 4 or TLR 8. These receptors are activated by several damage associated molecular proteins (DAMPs), including nuclear protein high mobility group box-1 (HMGB1), which is released from necrotic cells, and carbonyl products and advanced glycation endproducts (AGEs), which form during glycoxidative stress and/or ‘dicarbonyl stress’. AGEs, HMGB1, soluble RAGE and TLR 4 and

TLR 8 expression are elevated in SSc and their expression in scleroderma skin is more intense than in normal skin (25–28). Recent reports have indicated the role of TLR 4 and TLR 8 in innate immune signaling driving the persistent fibrotic response in SSc (26,27). They are putatively associated with profibrotic and calcification processes. In fact, we have preliminarily observed that *in vitro* stimulation of human cultured fibroblasts with AGE-modified proteins enhances the production of interleukin-6 (IL-6) and INF-1 (unpublished data). Whether this holds true for SSc derived fibroblasts needs further study. Activation of these receptors by the above-mentioned DAMPs could lead to profibrotic and osteogenic differentiation of dermal fibroblasts in SSc.

Currently, the calcification process is not assessed in clinical practice. Although patients with SSc generally do not have a disturbed calcium phosphate metabolism, several serum markers are available to assess the active calcification process *in vivo*. Recently, a nanoparticle-based assay (T50) has been developed that detects, in the presence of artificially elevated calcium and phosphate concentrations, the spontaneous transformation of spherical colloidal primary calciprotein particles (CPPs) to elongate crystalline secondary CPPs (29). T50 is associated with overall mortality in haemodialysis patients (30). In renal transplant recipients, a reduced serum T50 has been shown to be associated with increased risk of all-cause mortality, cardiovascular mortality, and graft failure (31,32). Importantly, in patients with systemic lupus erythematosus (SLE), which is also associated with premature cardiovascular complications, T50 was shown to be associated with ongoing systemic inflammation as mirrored by increased disease activity (33). These data underscore that T50 may also be of value in diseases, which are not accompanied by calcium and phosphate disturbances (33).

SSc is characterized by vasculopathy, low-grade inflammation, and ultimately fibrosis of skin and organ systems. It is mandatory to better characterize disease pathways and select biomarkers that occur early in the course of the disease, to identify high-risk patients and allow early, low cost and low risk disease-modifying therapies. Currently, opportunities for assessing the involvement of other organs are in routine care limited to esophageal scans (as a late sign of esophageal involvement) and effusion abnormalities as a sign of pulmonary involvement. Since calcification is strongly associated with local inflammatory disease, the process could very well occur in internal organs and serve as a proxy for long-term related complications. With the combination of nail-fold capillaroscopy and serology, it is currently possible to perform a reasonable SSc risk assessment, but accuracy is not high enough to detect early disease and potential organ involvement.

Interleukin-6 (IL-6) has been shown to play an important role in SSc by regulating the function of immune and non-immune cells. IL-6 is also known to induce TGFβ production and to enhance TGFβ-signaling in dermal and cardiac fibroblasts. Conversely, TGFβ regulates the expression of IL-6 by lung fibroblasts and airway smooth muscle cells. We have recently shown that fibroblasts are capable of producing extremely high levels of IL-6 locally after stimulation with TGFβ and other potential disease triggers [Atzeni EULAR 2019 submitted]. Dermal fibroblasts from patients with SSc express increased levels of IL-6. Increased serum IL-6 levels predict higher mortality risk, worse skin involvement and increased pulmonary decline in SSc. In congruence with fibroblasts, alveolar macrophages obtained from SSc-ILD patients also appear to produce IL-6 in extremely high levels after stimulation with granulocyte-macrophage colony-stimulating factor (GM-CSF). This is in line with the elevated serum levels of IL-6 early in the disease. Importantly, IL-6 is only predictive of ILD outcome in patients with early disease, where it is not predictive in more progressed stages, indicating a more inflammatory state in early disease. Recently, a profound impact of IL-6R blockade on the activated fibroblast phenotype was shown using explant dermal fibroblast cultures from SSc patients before and after treatment. These data underline the importance of IL-6 in the early inflammatory phases of SSc and could be a novel target for early treatment of SSc-ILD.

Diffuse pulmonary ossifications (DPO) are a well-known phenomenon in patients with fibrosing interstitial lung diseases (ILD), especially in patients with idiopathic pulmonary fibrosis (IPF) (34). Although IPF is a fibrotic disease, DPO is also frequently reported in more inflammatory ILD, i.e. in patients in a non-specific interstitial pneumonitis (NSIP) pattern on HRCT. Strikingly, DPO were observed especially in patients with NSIP with underlying SSc than in those with other causes of NSIP (34). Whether this a local process limited to the lungs, or an expression of a systemic calcification propensity is unknown. Furthermore, we hypothesize that the calcification process in the lungs is strongly related to local, as well as systemic inflammation in SSc, and the lung calcifications may be an early sign of pulmonary inflammation.

In conclusion, we hypothesize that assessment of the proinflammatory, profibrotic and osteogenic profile of the lungs in patients with SSc could serve as early markers of disease, and could potentially facilitate upfront treatment in patients with limited SSc-ILD. Furthermore, we hypothesize that proinflammatory, profibrotic and osteogenic pathways in lungs of SSc patients are interrelated, especially early in the disease course.

2 OBJECTIVES

1. To compare the proinflammatory, profibrotic and osteogenic cytokine profile in BAL fluid between SSc patients without ILD, with limited ILD, and with extensive ILD.
2. To compare the proinflammatory, profibrotic and osteogenic differentiation of fibroblasts isolated from lung BAL fluid and bronchial biopsies between SSc patients without ILD, with limited ILD, and with extensive ILD.
3. To compare the expression of markers involved in the calcification process in bronchial biopsies assessed by immunohistochemistry between SSc patients without ILD, with limited ILD, and with extensive ILD.
4. To assess the association of diffuse pulmonary ossifications on HRCT with the cytokine profile in BAL fluid, the fibroblast differentiation in lung fibroblasts, and calcification process in lung tissue in patients with SSc
5. To assess the cytokine profile in BAL fluid, the fibroblast differentiation in lung fibroblasts, and calcification process in lung tissue with systemic factors, including serum markers of inflammation, fibrosis, and the calcification process, non-invasive markers of micro- and macrovasculopathy, and the extent of skin and other internal organ complications in patients with SSc.

3 STUDY DESIGN

3.1 Design:

This is a substudy of the CALC-SSc study (NL65651.042.18, METC 2018.373). The nature of this substudy is explorative. It is an observational study with *case-control* and *prospective longitudinal* design, comparing the following groups of subjects:

- 10 SSc-ILD patients with limited lung disease, not qualifying for prompt treatment (*"limited SSc-ILD").*
- 5 SSc-ILD patients with extensive lung disease before initiation of first-line systemic therapy (i.e. cyclophamide or MMF) recommended by current international guidelines ("*extensive SSc-ILD"*).
- 5 SSc patients without SSc-ILD not receiving systemic anti-inflammatory treatment ("*SSc without ILD")*.

3.2 Duration and setting:

Patients will be recruited from the outpatient clinics of the department of Internal Medicine, division Vascular Medicine and department of Rheumatology and Clinical Immunology of the UMCG.

3.3 Schedule of visits:

For eligible patients who signed informed consent, an attempt will be made to plan all study activities on a single day. Wherever possible, data that has already been collected for clinical practice will be used in this study, to limit the burden of this study.

4 STUDY POPULATION

4.1 Population (base)

20 patients will be recruited from the out- and inpatients clinics of the department of internal medicine, rheumatology and clinical immunology, and pulmonology.

4.2 Inclusion criteria

In order to be eligible to participate in this study, a subject must meet several criteria.

All subjects in the patient groups (i.e. *"SSc without ILD", "limited SSc-ILD",* and "*extensive SSc-ILD group"*) should meet the following criteria:

- Age 18-70 years
- Written informed consent
- Formal diagnosis of Systemic Sclerosis, as determined by a total of ≥9 from adding the maximum weight (score) in each of the following categories (2013 ACR/EULAR criteria):
  - Skin thickening of the fingers of both hands extending proximal to the metacarpophalangeal joints (sufficient criterion) (*9 points*)
  - Skin thickening of the fingers (only count the higher score)
    - Puffy fingers (*2 points*)
    - Sclerodactyly of the fingers (distal to the metacarpophalangeal joints but proximal to the proximal interphalangeal joints) *(4 points*)
  - Fingertip lesions (only count the higher score)
    - Digital tip ulcers (*2 points*)
    - Fingertip pitting scars (*3 points*)
  - Telangiectasia (*2 points*)
  - Abnormal nailfold capillaries (*2 points*)
  - Pulmonary involvement (maximum score is 2)
    - Pulmonary arterial hypertension (*2 points*)
    - Interstitial lung disease (*2 points)*
  - Raynaud's phenomenon, defined as bi- or triphasic attacks of white or blue discoloration of fingers and/or toes, elicited by cold and/or emotion (*3 points*):
  - SSc-related autoantibodies (maximum score is 3)
    - Anticentromere (*3 points*)
    - anti–topoisomerase I [anti–Scl-70] (*3 points*)
    - anti–RNA polymerase III (*3 points*)

All subjects in the "*SSc-ILD group"* should meet the following criteria:

- A formal diagnosis of SSc, and a diagnosis of ILD based on:
  - typical SSc-ILD pattern on HRCT: nonspecific interstitial pneumonia (NSIP) and usual interstitial pneumonia (UIP), characterized by basal ground-glass opacities, reticular changes or honeycombing in the lung fields.

- SSc-ILD patients are classified as "*extensive*" when they present with:
  - Suggestive symptoms of ILD including dyspnea and non-productive cough, AND
  - Objective findings of *extensive* lung disease:
    - Decline in FVC% by >10% in the preceding 3–12 months, AND/OR
    - FVC <70%, AND/OR
    - >20% lung involvement on HRCT
- SSc-ILD patients are classified as "*limited*" when they present with:
  - No or only limited symptoms suggestive of ILD, AND
  - Objective findings of *limited* lung disease:
    - No decline in FVC% by >10% in the preceding 3–12 months, AND/OR
    - FVC >70%, AND/OR
    - <20% lung involvement on HRCT

4.3 Exclusion criteria

A potential subject who meets any of the following criteria will be excluded from participation in this study:

- Patients who are mentally incompetent and cannot sign a Patient Informed Consent or are unwilling to sign a Patient Informed Consent
- Vascular event in the preceding 3 months (35)
- Chemotherapy in the preceding 3 months (35)
- Inflammation of unknown origin, sepsis, or vasculitis
- ILD as a result of other connective tissue diseases.
- Idiopathic pulmonary fibrosis.
- Pulmonary Arterial Hypertension based on cardiac ultrasound and confirmed with right-sided cardiac catheterization.
- Other lung diseases associated with inflammation or fibrosis (e.g. COPD, asthma, cystic fibrosis, lung cancer).
- Severe pulmonary function impairment due to which bronchoscopy is too high a risk.
- Current (within 6 months) smoking.
- Current use of any systemic anti-inflammatory or steroid treatment, with the exception of NSAIDs, including:
  - non-biologicals such as MTX, MMF, azathioprine, and cyclophosphamide
  - biologicals such as Il6 receptor antagonists or rituximab
  - steroids including oral and intravenous prednisolone
- Previous use of systemic anti-inflammatory treatment, with the exception of NSAIDs, including:
  - non-biologicals such as MTX, MMF, azathioprine, and cyclophosphamide
  - biologicals such as Il6 receptor antagonists or rituximab

  - only steroid use for less than 7 days, more than 6 months ago, are allowed
- Current use of corticosteroid inhalation therapy.
- Current (within <7 days) use of antibiotics.
- Current use of anti-coagulant or anti-platelet therapy to minimize bleeding risk after bronchoscopy.

4.4 Sample size calculation

Since no previous studies have investigated levels of proinflammatory, profibrotic, or osteogenic markers in BAL fluids of limited SSc-ILD patients, no formal sample size can be calculated. Since IL-6 has been investigated most extensively in previous cohorts, and since IL-6 is a well-validated cytokine, for which direct treatment options are available, we have chosen IL-6 as the primary outcome parameter for this study. In a previous study in patients with severe SSc-ILD compared with those without severe SSc-ILD, BAL IL-6 levels were 221 (SD 62) and 121 (SD 55) respectively, and 50 (SD 8) healthy controls (66)12. Although this study is not comparable to the current study, as patients in both groups had complaints or dyspnoea and a decreased FVC to an extent that is not present in limited ILD, and HRCT were not used for assessing disease severity it gives an indication of the variance of this biomarker. The effect size was 1.7, which would necessitate a sample size of 8 limited SSc-ILD patients and 4 SSc patients without ILD. In order to anticipate unexpected dropouts, we will include 10 participants with limited SSc-ILD, 5 SSc patients without ILD, and 5 patients with extensive SSc-ILD

5 TREATMENT OF SUBJECTS

Not applicable

6 NON-INVESTIGATIONAL PRODUCT

Not applicable.

7 METHODS

7.1 Study parameters/endpoints

Outcome parameters will be assessed in a blinded manner. Details of all procedures will be extensively explained in section 7.3.

7.1.1 Main study parameter/endpoint

1. Level of IL-6 in BAL fluid

7.1.2 Secondary study parameters/endpoints

2. Inflammatory, fibroproliferative, and osteogenic cytokines obtained from BAL fluids and bronchial biopsies, including:
    a. Immunological BAL: alveolar macrophages, lymphocytes, CD4/CD8-ratio, neutrophils, eosinophils, mast cells by flow cytometry and cytospins.
    b. Cytokines and chemokines including CXCL4 (chemokine [C-X-C motif] ligand 4), CCL18 (chemokine [C-C motif] ligand 18); Krebs von den Lungen-6 (KL-6)
3. Isolation of fibroblasts from BAL and bronchial biopsies to study proinflammatory, profibrotic and osteogenic differentiation by expression of alpha smooth muscle actin (αSMA) and interferon-regulated genes IFI44 (interferon-induced protein 44); IRF5 (interferon regulatory factor 5)
4. Degree of tissue markers involved in the calcification process and SSc progression in bronchial biopsies: degree of fibrosis (TGFβ, collagen); αKlotho; FGF23; myxovirus resistance gene A (MxA) which is a marker for IFNα, TLR 4 and TLR 8;
5. Levels of the serum markers of inflammation and fibrosis (IL-6, CRP, CXCL4, CCL18, and KL-6, and calcification process (T50; calcium; phosphorus; parathormone; fetuin-A; FGF23; αKlotho)
6. Degree of pulmonary involvement of ILD and pulmonary ossifications: lung function (FVC, FEV1, DLCO) and HRCT (see details below);
7. Degree of vasculopathy by: noninvasive nailfold capillary microscopy; arterial stiffness (carotid-brachial and carotid-radial); digital artery involvement as assessed with ultrasound (see details below);
9. Clinical parameters on SSc organ and degree of skin and additional organ involvement, SSc-antibody profile, medication use, and comorbidities:
    a. cardiac involvement by cardiac ultrasound
    b. renal involvement by 24-hours creatinine clearance, albumin excretion/24 hours, and glomerular erythrocyturia by microscopy

c. gastro-intestinal involvement by conventional esophageal motility scan (these data are available from clinical practice)
d. skin involvement by modified Rodnan Skin Score.

7.2 Randomisation, blinding and treatment allocation

Not applicable.

7.3 Study procedures

For all assessments specific standard operating procedures (“SOPs”) are available since all procedures are already in use for clinical practice. The protocols will be adapted for use in this study. Attempts will be made to plan all study procedures on a single day. Vascular function studies will be performed at our Vascular Lab. Bronchoscopies will be performed at the department of pulmonology.

7.3.1 Routine clinical assessments from which data will be used in the current study.

All patients with SSc who visit the UMCG are subjected to the so called “zorgpad systemische sclerose”. This entails a yearly detailed assessment of organ involvement using a predefined national protocol. Data coming from these assessments will be used in the current study, the procedures will not be repeated. These include the following procedures:

- Physical examination
- full lab assessment
- urine analysis
- lungfunction tests
- vascular lab

On indication:

- lung ultrasound
- cardiac ultrasound
- HRCT of thoraxoesophageal scintigraphy
- bronchoscopy with BAL and bronchial biopsies (rarely performed for clinical reasons, only when there is clinical doubt on the underlying lung disease)

All other procedures are considered “Study procedures” and are outlined in paragraph 7.3.2.

7.3.1.1 Clinical parameters

Patients visiting the ambulatory clinical for the “zorgpad systemische sclerose” are subjected to a physical examination, which included a full cardiopulmonary check-up, assessment of

SSc-related skin abnormalities such as digital ulcers, pitting scars, teleangiectasia, sclerodactyly, scleroderma, puffy fingers, calcinosis cutis, and tendon friction rubs. Additionally, the Modified Rodnan Skin Score (mRSS) are assessed in which 17 body areas are examined by clinical palpation and scored based on examiner judgment of skin thickness on a 4-point ordinal scale (0 = normal thickness; 1 = mild thickening; 2 = moderate thickening; and 3 = severe thickening). The range of the mRSS is thus from 0 (no skin thickening) to 51 (grade 3 change in all 17 body areas)). The areas examined in the mRSS scheme include the right and left fingers, hands, forearms, upper arms, thighs, lower legs and feet, as well as the face, anterior chest and abdomen (39). At least yearly, a full lab assessment is performed which includes: blood count, urate, nt-pro-BNP, calcium, albumin, phosphorus, parathormone, CRP and ESR. These are measured by routine clinical practice techniques. Additionally, the degree of cardiac involvement is assessed by cardiac ultrasound performed by the cardiologist. Renal involvement by 24-hours creatinine clearance, albumin excretion/24 hours, and glomerular erythrocyturia by microscopy. Potential pulmonary involvement is defined as the diffusion capacity of the lung for carbon monoxide (uncorrected for alveolar volume, DLCO) or forced vital capacity (FVC) <70% assessed by pulmonary function tests. Additionally, a lung ultrasound is performed using the simplied scoring system of 14 intercostal spaces, assessing B-lines and pleural thickening and irregularities. If necessary, when the DLCO or FVC is abnormal or when judged by the physician, HRCT and/or cardiac US are performed as additional tests.
Definite pulmonary involvement is defined as an interstitial lung disease pattern on HRCT or documented pulmonary hypertension by mean pulmonary artery pressure 25 mmHg at rest measured by right heart catheterisation. Oesophageal involvement is assessed by oesophageal scintigraphy with 99mTc colloid.

7.3.1.2 Standard vascular lab assessments

Nail-fold capillaroscopy is performed using a high-resolution camera, using the method as described by van Roon as part of the routine clinical assessments (40). The following characteristics are assessed: widened capillaries, giant capillaries, capillary density, and total loop width, which have been shown to be reproducible parameters with high intra-class correlation coefficients (41). From these images and SSc patterns ('Early', 'Active' or 'Late'), defined by Cutolo (42), summarized in the "microangiopathy evolution score" (MES) (43) and DU risk score (measured by Capillaroscopic Skin Ulcer Risk Index (CSURI) (44), Prognostic Index for Digital Lesions (PILD) (45) are assessed.

Pulse Wave Velocity: Carotid to brachial and brachial to radial and brachial to ulnar PWV (cbPWV and crPWV) will be assessed with the Sphygmocor apparatus as developed by

O'Rourke (Sphygmocor EM-3; AtCor Medical, Sydney, Australia) (60). In brief, a high-fidelity pressure sensor will be used to flatten but not occlude the artery in question, using gentle pressure. When the two surfaces are flattened, circumferential pressures are equalized and an accurate pressure waveform can be recorded; this technique is termed applanation tonometry (61).

Digital artery involvement assessed with ultrasound: Digital artery involvement will be assessed with ultrasound according to Schmidt et al. Hereby we use Doppler and spectral analysis of the brachial, radial and ulnar artery. Additionally, finger pressure measurements of all 5 fingers of both hands will be performed (62–64).

7.3.2 Study procedures.

7.3.2.1 Blood sampling

1x 10 cc tubes of blood will be collected for plasma and 1x 10 cc tubes for serum storage.
3x 10 cc EDTA tubes will be collected for PBMCs isolation.
1x 6 cc tube of blood will be collected for RNA for interferon signature (PaxGene).

Collection of blood will be preferably combined with route assessment, limiting extra venipunctures.

IL-6, CRP, CXCL4, CCL18, KL-6, calcium; phosphorus; parathormone; fetuin-A; FGF23; αKlotho will be measured by commercially available ELISAs, or routine procedures available at the UMCG clinical chemical lab. T50 by maturation time of calciprotein particles in serum. From whole blood, peripheral blood mononuclear cells (PBMCs) will be isolated and stored at -80 °C in liquid nitrogen for determination of angiogenic T cells and Tie2 positive monocytes. Frequencies of CD3+, CD4+ and CD8+ angiogenic T cell populations will be analysed in thawed PBMCs by flow cytometry on the LSR II (BD Bioscience) using antibodies against surface markers CD3-PerCP, CD8-AF700, CD31-PB and CXCR4-BV605 (CD184) (all from Biolegend). Compensation will be done using isotype controls.

7.3.2.2 Bronchoscopy

Preferably, patients will be included for whom a bronchoscopy is performed for clinical purposes. In these patients, the number of biopsies will be no more than 8 in total. Additionally, immunologic BAL is a routine procedure in patients will ILD, and this fluid will be used for the measurement of the additional markers for the study.

Bronchoscopy procedure

Bronchoscopy will be performed using established guidelines (49). Patients are not allowed to drink or eat food after 0.00 the night prior to the bronchoscopy. On arrival for bronchoscopy, two ml of lidocaine 2% will be instilled in the mouth 3-5 times, on the vocal cords and into the trachea to inhibit coughing. The total lidocaine dose must not exceed 3 mg/kg. Bronchoscopy is performed during conscious sedation, which is carried out according to routine clinical practice using intravenously administered propofol or midazolam. Then, a bronchial wash is performed with 20 ml saline according to routine clinical practice. Next, bronchial biopsies are taken from subsegmental carinae in the right or left lower lobe using cup forceps. A maximum of 8 biopsies will be taken. Next, bronchial epithelial cells will be harvested from the right main bronchial by brushing as described elsewhere (50). If hemostasis is necessary, xylometazoline will be applied locally.

Analysis of lung BAL fluid

BAL analysis will be performed using standard techniques, described extensively earlier (51–53). The volume of the whole BAL sample will be determined. The samples will be gently mixed using a wide bore plastic pipette, and placed in a shaking water bath at 37°C for 15 min to ensure complete homogenization. The total cell count will be measured using a standard haemocytometer. The homogenized sputum will be centrifuged at 350×g for 10 min. The supernatant will be aspirated and stored at -70°C pending analysis. The cell pellet will be resuspended in 10 mL phosphate-buffered saline, filtered through a nylon gauze (pore size approximately 1 mm), cytocentrifuged for 3 min at 1,500 rpm (Shandon cytocentrifuge 3; Shandon Southern Instruments, Sewickley, PA, USA), and stained with May-Grünwald-Giemsa. Differential cell counts will be performed by counting at least 500 cells on one coded cytospin in a blind way by a qualified cytopathologist. In addition, if required, lysis of RBC will be performed before processing. A BAL sample will be considered adequate when the amount of fluid instilled is at least 100 mL, and recovered BAL fluid is at least 10 mL (excluding the first portion). For IL-8 measurements, BAL supernatant is first concentrated using filters (Centricon-3; Millipore; Bedford, MA) (50). IL-6 (Biolegend, San Diego, CA, USA) (54) and CCL18 (55) are measured by ELISA, CXCL4 by fluorescence-based immunoassay (Merk Millipore, Billerica, MA, USA) (56), KL-6 by chemiluminescent enzyme immunoassay (57).

Handling of bronchial biopsies

The first 4 biopsies will be processed for scRNA-Seq analysis. Two biopsies will be fixed in 4% neutral buffered formalin, processed and embedded in paraffin. A haematoxylin-eosin (H-E) staining will be used for judging the biopsy quality. These HE slides allow a first

impression of the degree of inflammation and gives an indication of the types of inflammatory cells. A general characterization of the infiltrate will be performed. Lymphocytes: T cell subsets (CD3, CD4, CD8, CXCR3, CXCR10), total B cells (CD20). Mast cells: Giemsa, tryptase. Neutrophils: neutrophil elastase, CD11 and CD18 expression. Eosinophils: EG2. Macrophages: CD68. NFkappaB expression. In addition, 2 biopsies will be embedded in Tissue Tek mounting medium, snap frozen in liquid isopentane and finally stored at -80°C. One biopsy will be stored in RNAse free medium at -80 for isolation of RNA.

Fibroblasts will be sourced from BAL fluid and bronchial biopsies. This has previously been shown to be feasible in material from patients with various types of interstitial lung diseases (58). An aliquot of BAL-sample or collagenase-digested bronchial biopsy specimen will be centrifuged (300 g, 10 min) and plated at a density of approximately 40,000 cells/cm2 in a medium consisting of Minimum essential medium Eagle α modification (Sigma-Aldrich, Inc, St Louis, MO, USA) supplemented with 13% heat-inactivated fetal bovine serum (PromoCell, Heidelberg, Germany), 2 mM L-glutamine, 100 U/ml penicillin, 0.1 g/l streptomycin, 2.5 mg/l amphotericin B and 10 mM HEPES (all from Sigma-Aldrich). Obtained lung fibroblasts will be stimulated with TGF-β to study their *in vitro* transcriptome (i.e. IL-6, IL-8,and CXCL-1) production and profibrotic signature (RNA expression (rtPCR) and protein production (ELISA) of Connective Tissue Growth Factor (CTGF), collagen 1 α (Col1α), α smooth muscle actin (α SMA)). To test whether this profibrotic signature is IL-6 mediated, cultured fibroblast will be incubated with either anti-IL-6 or anti-sIL-6 receptor antibody alone, or both, as described earlier (59).

Evaluation High Resolution CT of lungs:

<u>No additional HRCT scans will be performed for this study.</u> In all patients included, the HRCT scans that have already been taken for routine care will be carefully reevaluated.

Two independent pulmonologists will review clinically obtained HRCT images without reference to any clinical or histologic information. All findings will be scored on a lobar basis; the left upper lobe will be considered as two lobes (the lingula will be considered as a separate lobe). Diagnosis and terminology will be based on the Fleischner Society: Glossary of Terms for Thoracic Imaging. Pulmonary involvement on HRCT will be evaluated with the Warrick score (46) expressed in the following semi-quantitative scoring: [0 = normal (0 points); 1 = mild (< 8 points); 2 = moderate (from 8 to 15 points) and 3 = marked (> 15 points).

Evaluation of pulmonary ossification will be based on a previous publication. Small opacities will be recorded as pulmonary ossifications if they were intrapulmonary nodules of calcific

attenuation identifiable on bone window settings with a width of 2500 HU and a level of 500 HU and maximal short-axis diameter less than 4 mm. Calcified nodules greater than 4 mm in diameter and/or those suspected of being calcified granulomata will be recorded separately. All POs will be analyzed for shape and number. lung, and thus, individual dendriform structures will not be recorded.

7.4 Withdrawal of individual subjects

Subjects can leave the study at any time for any reason if they wish to do so without any consequences. The investigator can decide to withdraw a subject from the study for urgent medical reasons.

7.4.1 Specific criteria for withdrawal

Not applicable.

7.5 Replacement of individual subjects after withdrawal

If the subject withdraws, he or she will not be replaced by a new subject.

7.6 Follow-up of subjects withdrawn from treatment

Not applicable.

7.7 Premature termination of the study

No predefined criteria for premature termination of the study have been adopted. If, however, during the conductance of the study information becomes available showing that continuation of the study would result in a significant risk for the patients, the principal investigator and project leader will decide to terminate the study.

8 SAFETY REPORTING

8.1 Temporary halt for reasons of subject safety

In accordance to section 10, subsection 4, of the WMO, the sponsor will suspend the study if there is sufficient ground that continuation of the study will jeopardise subject health or safety. The sponsor will notify the accredited METC without undue delay of a temporary halt including the reason for such an action. The study will be suspended pending a further positive decision by the accredited METC. The investigator will take care that all subjects are kept informed.

8.2 AEs, SAEs and SUSARs

8.2.1 Adverse events (AEs)

Adverse events are defined as any undesirable experience occurring to a subject during the study, whether or not considered related to the investigational product. All adverse events reported spontaneously by the subject or observed by the investigator or his staff will be recorded.

8.2.2 Serious adverse events (SAEs)

A serious adverse event is any untoward medical occurrence or effect that

- results in death;
- is life-threatening (at the time of the event);
- requires hospitalisation or prolongation of existing inpatients' hospitalisation;
- results in persistent or significant disability or incapacity;
- is a congenital anomaly or birth defect; or
- any other important medical event that did not result in any of the outcomes listed above due to medical or surgical intervention but could have been based upon appropriate judgment by the investigator.

An elective hospital admission will not be considered as a serious adverse event.
The investigator will report all SAEs to the sponsor without undue delay after obtaining knowledge of the events, except for the following SAEs: <*specify which SAEs do not require immediate reporting by the investigator to the sponsor, if applicable*> .

The sponsor will report the SAEs through the web portal *ToetsingOnline* to the accredited METC that approved the protocol, within 7 days of first knowledge for SAEs that result in death or are life threatening followed by a period of maximum of 8 days to complete the initial preliminary report. All other SAEs will be reported within a period of maximum 15 days after the sponsor has first knowledge of the serious adverse events.

8.2.3 Suspected unexpected serious adverse reactions (SUSARs)

Adverse reactions are all untoward and unintended responses to an investigational product related to any dose administered.

Unexpected adverse reactions are SUSARs if the following three conditions are met:

1. the event must be serious (see chapter 9.2.2);
2. there must be a certain degree of probability that the event is a harmful and an undesirable reaction to the medicinal product under investigation, regardless of the administered dose;
3. the adverse reaction must be unexpected, that is to say, the nature and severity of the adverse reaction are not in agreement with the product information as recorded in:
   - Summary of Product Characteristics (SPC) for an authorised medicinal product;
   - Investigator's Brochure for an unauthorised medicinal product.

The sponsor will report expedited the following SUSARs through the web portal *ToetsingOnline* to the METC:

- SUSARs that have arisen in the clinical trial that was assessed by the METC;
- SUSARs that have arisen in other clinical trials of the same sponsor and with the same medicinal product, and that could have consequences for the safety of the subjects involved in the clinical trial that was assessed by the METC.

The remaining SUSARs are recorded in an overview list (line-listing) that will be submitted once every half year to the METC. This line-listing provides an overview of all SUSARs from the study medicine, accompanied by a brief report highlighting the main points of concern.

The expedited reporting of SUSARs through the web portal Eudravigilance or ToetsingOnline is sufficient as notification to the competent authority.

The sponsor will report expedited all SUSARs to the competent authorities in other Member States, according to the requirements of the Member States.

The expedited reporting will occur not later than 15 days after the sponsor has first knowledge of the adverse reactions. For fatal or life threatening cases the term will be maximal 7 days for a preliminary report with another 8 days for completion of the report.

8.3 Annual safety report

In addition to the expedited reporting of SUSARs, the sponsor will submit, once a year throughout the clinical trial, a safety report to the accredited METC, competent authority, and competent authorities of the concerned Member States.

This safety report consists of:

- a list of all suspected (unexpected or expected) serious adverse reactions, along with an aggregated summary table of all reported serious adverse reactions, ordered by organ system, per study;
- a report concerning the safety of the subjects, consisting of a complete safety analysis and an evaluation of the balance between the efficacy and the harmfulness of the medicine under investigation.

8.4 Follow-up of adverse events

All AEs will be followed until they have abated, or until a stable situation has been reached. Depending on the event, follow up may require additional tests or medical procedures as indicated, and/or referral to the general physician or a medical specialist.
SAEs need to be reported till end of study within the Netherlands, as defined in the protocol.

8.5 [Data Safety Monitoring Board (DSMB) / Safety Committee]

Not applicable.

9 STATISTICAL ANALYSIS

9.1 Primary study parameter(s)

Since this is a pilot study, statistics will be primarily descriptive. Participants will be described with their demographical and medical data. Further, all study parameters will be evaluated with descriptive statistical methods. Results will be expressed as mean with standard deviation for normal parametric data and median and interquartile range for non-parametric data. Dichotomous, categorical, or ordinal data will be described as numbers and percentages. Additionally, effect sizes between group differences will be expressed, using cohens D.

Formal statistical testing will be considered explorative. The overall between-group differences will be tested using One-way Analysis Of Variance (ANOVA), correction for multiple comparisons. The primary comparison in this study will be comparing IL-6 levels in BAL fluid between participants in the limited SSc-ILD with participants in SSc without ILD group. Secondarily, a comparison will be made between extensive SSc-ILD group and limited SSc-ILD. Difference between patient groups will be tested using student's t-test or if non-normally distributed with the Mann-Whitney U test. A two-sided *P*-value less than 0.05 will be considered statistical significant.

9.2 Secondary study parameter(s)

Binary or ordinal data will be compared with Chi square or Fisher's exact test.
Spearman's rho will be applied for association. Change in parameters over time in one group will be tested using student's t-test for paired variables, or the Wilcoxon signed-rank test if non-normally distributed.

9.3 Other study parameters

Not applicable.

9.4 Interim analysis

Not applicable.

10 ETHICAL CONSIDERATIONS

10.1 Regulation statement

The study will be conducted according to the principles of the Declaration of Helsinki (64th WMA General Assembly, Fortaleza, Brazil, October 2013) and in accordance with the Medical Research Involving Human Subjects Act (WMO). The Medical Ethical Committee of the University Medical Center in Groningen has to approve the study.

10.2 Recruitment and consent

Patients will be recruited from the outpatient clinics of the UMCG. Only the treating physician will select those patients that may be potentially eligible for the study. These patients will be invited by the investigator to participate in the study by sending a letter as well as the patient information form. In this information, patients will find a full explanation of the study, advantages and disadvantages of participating, and contact information of the research team members working on this study.
Moreover, the letter contains contact information of an independent physician, to whom subjects can address questions about the research before, during and after the study. The patients will be given 2 weeks to consider their decision. If the patient is interested in the study, he or she will be asked to return the reply form to the investigator. Written informed consent will be obtained before any study activities will be performed. The patient information letter and informed consent form are attached as a separate document.

The screening procedure will be as follows. The treating physician will pre-screen potentially eligible patients on the major in- and exclusion criteria. Importantly, patients using anti-coagulant drugs and anti-platelet agents will not be pre-selected. After the consent procedure, the investigator will assess all in- and exclusion criteria again using a specifically designed section of the CRF be ticking all items of the list.

10.3 Objection by minors or incapacitated subjects

Not applicable.

10.4 Benefits and risks assessment, group relatedness

Participation in the study is on a voluntary basis. Patients will receive restitution of all costs of transportation and 50 euro as compensation for the time to perform study procedures. Patients will not receive priority for treatment of other diseases in the clinic during this study. Also, participants will not be informed by the investigator concerning the results of the tests

performed for research purposes in the study (i.e. those mentioned under: 7.3.2:“ Study procedures” which includes “Blood sampling” (7.3.2.1) and “Bronchoscopy” (7.3.2.2) results). The treating physician will remain responsible for informing the participant on results assessed outside of the setting of this study (mentioned under 7.3.1.1 and 7.3.1.2), and to perform addition tests and treatment if considered indicated. Participation in the proposed study is accompanied with only minor risks. The blood samples will be drawn by means of venipunctures that will be performed during the visit to the outpatient clinic and the number of venipunctures will therefore not differ from that performed in clinical practice. Special attempts will be made to combine study procedures and venipunctures with regular visits, hence keeping the number of extra punctures to a minimum. Bronchoscopy may result in minor discomfort (not needing medical attention) in <10%, and in <1% it may result in more severe complications (pneumonia and bleeding) needing medical attention and intervention. By excluding participants using anticoagulants or anti-platelet drugs, the risk of bleeding is limited as much as possible.

10.5 Compensation for injury

The sponsor/investigator has a liability insurance which is in accordance with article 7 of the WMO.

The sponsor (also) has an insurance which is in accordance with the legal requirements in the Netherlands (Article 7 WMO). This insurance provides cover for damage to research subjects through injury or death caused by the study.
The insurance applies to the damage that becomes apparent during the study or within 4 years after the end of the study.

10.6 Incentives

All travel expenses will be reimbursed during the study following the regulations of the UMCG. Additionally, patients will be offered 50 euro as compensation for the time to perform study procedures.

11 ADMINISTRATIVE ASPECTS, MONITORING AND PUBLICATION

11.1 Handling and storage of data and documents

For the study, a clinical database and electronic Case Report Form (eCRF) will be designed. Web-based access will be secured and the system has an audit trail to track data changes. Access rights will depend on the role of each individual. Investigators and staff can complete the CRF and update data if necessary, the monitor can perform data validation plan and issue data clarification requests. Data management will perform the data validation either by the pre-programmed electronic edit checks or by manual queries. At the end of the study when all queries have been solved, data management will request approval from the investigator to lock the database. Data will never be shared with third parties.
A subject identification code list will be made to link the data to the subject in order to be able to trace data to an individual subject. This code will not be based on patient initials and birth-date. Patients will receive a code starting with an "S", followed by a number, which will be assigned chronologically to each patient being screened for the study, starting with "S01". The key to the code will be safeguarded by the investigator since the data will be kept for a period of 15 years. The handling of personal data will comply with the Dutch Personal Data Protection Act (in Dutch: AVG (Algemene Verordening Gegevensbescherming). Biomaterials will also be encrypted by the subject identification code. Tissue from the lung biopsies will be kept for a maximum period of 15 years.

11.2 Monitoring and Quality Assurance

Since this is a low-risk study, monitoring of the conduct of the study will be performed by a colleague from a different department, not involved in the study who has been trained for performing monitoring visits.
Monitoring will be performed by certified personnel. During monitoring visits the following will be verified: if compliance with the protocol is maintained, qualification of investigational staff, if investigator has access to adequate number of subjects, if informed consents are properly signed and dated, if (S)AEs and SUSARs have been recorded and reported adequately, maintenance and calibration equipment, and recording subject withdrawal, non-compliance. This will be reported on monitoring visit forms. Please refer to the monitoring plan for details.

11.3 Amendments

A 'substantial amendment' is defined as an amendment to the terms of the METC application, or to the protocol or any other supporting documentation, that is likely to affect to a significant degree:

- the safety or physical or mental integrity of the subjects of the trial;
- the scientific value of the trial;
- the conduct or management of the trial; or
- the quality or safety of any intervention used in the trial.

All substantial amendments will be notified to the METC and to the competent authority.

Non-substantial amendments will not be notified to the accredited METC and the competent authority, but will be recorded and filed by the sponsor.

11.4 Annual progress report

The sponsor/investigator will submit a summary of the progress of the trial to the accredited METC once a year. Information will be provided on the date of inclusion of the first subject, numbers of subjects included and numbers of subjects that have completed the trial, serious adverse events/ serious adverse reactions, other problems, and amendments.

11.5 Temporary halt and (prematurely) end of study report

The sponsor will notify the accredited METC and the competent authority of the end of the study within a period of 90 days. The end of the study is defined as the last patient's last visit.

The sponsor will notify the METC immediately of a temporary halt of the study, including the reason of such an action.

In case the study is ended prematurely, the sponsor will notify the accredited METC and the competent authority within 15 days, including the reasons for the premature termination.

Within one year after the end of the study, the investigator/sponsor will submit a final study report with the results of the study, including any publications/abstracts of the study, to the accredited METC and the Competent Authority.

11.6 Public disclosure and publication policy

Publication policy is in agreement with the CCMO publication statement. Nor the sponsors, nor the principal investigator, nor the subsidizing party has a right of veto regarding the way of publishing the results.

11.7 STRUCTURED RISK ANALYSIS

Potential issues of concern

This is not an interventional study. This study has no specific benefits for the participating patients and the study also has no major risks. A bronchoscopy with BAL and bronchial biopsies is a standard procedure. However, this procedure is performed for the sole purpose of the study in most participants.

Synthesis

Bronchoscopy may result in minor discomfort (not needing medical attention) in <10%, and in <1% it may result in more severe complications (pneumonia and bleeding) needing medical attention and intervention. By excluding participants using anticoagulants or anti-platelet drugs, the risk of bleeding is limited as much as possible. Additionally, the procedure is performed by a very experienced pulmonologist.

To answer our main objectives, a bronchoscopy met BAL and bronchial biopsies is needed in patients in whom currently this procedure is not routinely performed. This study will give a unique insight into the early changes that occur in the disease. This is important, because no disease modifying treatment is available. An early intervention of the inflammatory components of the disease is a logical and realistic approach since new biologicals are currently available that could be prescribed for this group. It is however unclear whether patients with early lung involvement really have an inflammatory profile. This study is the first step to a new treatment approach, and is believed to be essential and ethically sound to be performed before subjecting patients to an interventional study of which the risks a much higher.

Additionally, attempts will be made to have patients visit our center only once for assessment of all study parameters. Blood drawing will be combined with routine clinical practice assessments to minimize the venipuncture burden.

12 REFERENCES

1. Hao Y, Hudson M, Baron M, Carreira P, Stevens W, Rabusa C, et al. Early Mortality in a Multinational Systemic Sclerosis Inception Cohort. Arthritis Rheumatol (Hoboken, NJ). 2017;69(5):1067–77.
2. Denton CP, Khanna D. Systemic sclerosis. Lancet (London, England). 2017 Apr 13;
3. Koenig M, Joyal F, Fritzler MJ, Roussin A, Abrahamowicz M, Boire G, et al. Autoantibodies and microvascular damage are independent predictive factors for the progression of Raynaud's phenomenon to systemic sclerosis: a twenty-year prospective study of 586 patients, with validation of proposed criteria for early systemic sclerosi. Arthritis Rheum. 2008 Dec;58(12):3902–12.
4. Spencer-Green G. Outcomes in primary Raynaud phenomenon: a meta-analysis of the frequency, rates, and predictors of transition to secondary diseases. Arch Intern Med. 1998;158(6):595–600.
5. Solomon JJ, Olson AL, Fischer A, Bull T, Brown KK, Raghu G. Scleroderma lung disease. Eur Respir Rev. 2013;22(127):6–19.
6. Steen VD, Medsger TA. Changes in causes of death in systemic sclerosis, 1972-2002. Ann Rheum Dis. 2007 Jul;66(7):940–4.
7. Greenblatt MB, Aliprantis AO. The immune pathogenesis of scleroderma: context is everything. Curr Rheumatol Rep. 2013 Jan;15(1):297–8.
8. Minier T, Guiducci S, Bellando-Randone S, Bruni C, Lepri G, Czirjak L, et al. Preliminary analysis of the very early diagnosis of systemic sclerosis (VEDOSS) EUSTAR multicentre study: evidence for puffy fingers as a pivotal sign for suspicion of systemic sclerosis. Ann Rheum Dis. 2014 Dec;73(12):2087–93.
9. Steen VD, Ziegler GL, Rodnan GP, Medsger Jr TA. Clinical and laboratory associations of anticentromere antibody in patients with progressive systemic sclerosis. Arthritis Rheum. 1984 Feb;27(2):125–31.
10. Vayssairat M, Hidouche D, Abdoucheli-Baudot N, Gaitz JP. Clinical significance of subcutaneous calcinosis in patients with systemic sclerosis. Does diltiazem induce its regression? Ann Rheum Dis. 1998 Apr;57(4):252–4.
11. Baron M, Pope J, Robinson D, Jones N, Khalidi N, Docherty P, et al. Calcinosis is associated with digital ischaemia in systemic sclerosis-a longitudinal study. Rheumatology (Oxford). 2016 Dec;55(12):2148–55.
12. Davies CA, Jeziorska M, Freemont AJ, Herrick AL. Expression of osteonectin and matrix Gla protein in scleroderma patients with and without calcinosis. Rheumatology (Oxford). 2006 Nov;45(11):1349–55.
13. Imura A, Tsuji Y, Murata M, Maeda R, Kubota K, Iwano A, et al. alpha-Klotho as a regulator of calcium homeostasis. Science. 2007 Jun 15;316(5831):1615–8.
14. Hajialilo M, Noorabadi P, Tahsini Tekantapeh S, Malek Mahdavi A. Endothelin-1, alpha-Klotho, 25(OH) Vit D levels and severity of disease in scleroderma patients. Rheumatol Int. 2017;37(10):1651–7.
15. Mazzotta C, Manetti M, Rosa I, Romano E, Blagojevic J, Bellando-Randone S, et al. Proangiogenic effects of soluble alpha-Klotho on systemic sclerosis dermal microvascular endothelial cells. Arthritis Res Ther. 2017 Feb 10;19(1):20–7.
16. Irkle A, Vesey AT, Lewis DY, Skepper JN, Bird JL, Dweck MR, et al. Identifying active vascular microcalcification by (18)F-sodium fluoride positron emission tomography. Nat Commun. 2015 Jul 7;6:7495.
17. Cannarile F, Valentini V, Mirabelli G, Alunno A, Terenzi R, Luccioli F, et al. Cardiovascular disease in systemic sclerosis. Ann Transl Med. 2015 Jan;3(1):8-5839.2014.12.12.
18. Khurma V, Meyer C, Park GS, McMahon M, Lin J, Singh RR, et al. A pilot study of subclinical coronary atherosclerosis in systemic sclerosis: coronary artery calcification in cases and controls. Arthritis Rheum. 2008 Apr 15;59(4):591–7.
19. Heron E, Hernigou A, Chatellier G, Fornes P, Emmerich J, Fiessinger JN. Intracerebral calcification in systemic sclerosis. Stroke. 1999;30(10):2183–5.
20. Belloli L, Ughi N, Massarotti M, Marasini B, Biondi ML, Brambilla G. Role of fetuin-A in systemic sclerosis-associated calcinosis. J Rheumatol. 2010 Dec;37(12):2638–9.

21. Schafer C, Heiss A, Schwarz A, Westenfeld R, Ketteler M, Floege J, et al. The serum protein alpha 2-Heremans-Schmid glycoprotein/fetuin-A is a systemically acting inhibitor of ectopic calcification. J Clin Invest. 2003 Aug;112(3):357–66.
22. Wang XQ, Hayes MT, Kempf M, Fraser JF, Liu PY, Cuttle L, et al. Fetuin-A: a major fetal serum protein that promotes “wound closure” and scarless healing. J Invest Dermatol. 2008;128(3):753–7.
23. Brkic Z, van Bon L, Cossu M, van Helden-Meeuwsen CG, Vonk MC, Knaapen H, et al. The interferon type I signature is present in systemic sclerosis before overt fibrosis and might contribute to its pathogenesis through high BAFF gene expression and high collagen synthesis. Ann Rheum Dis. 2016 Aug;75(8):1567–73.
24. Moritz F, Schniering J, Distler JHW, Gay RE, Gay S, Distler O, et al. Tie2 as a novel key factor of microangiopathy in systemic sclerosis. Arthritis Res Ther. 2017;19(1):102–5.
25. Yoshizaki A, Komura K, Iwata Y, Ogawa F, Hara T, Muroi E, et al. Clinical significance of serum HMGB-1 and sRAGE levels in systemic sclerosis: association with disease severity. J Clin Immunol. 2009;29(2):180–9.
26. Bhattacharyya S, Varga J. Emerging roles of innate immune signaling and toll-like receptors in fibrosis and systemic sclerosis. Curr Rheumatol Rep. 2015 Jan;17(1):474-014-0474-z.
27. Ah Kioon MD, Tripodo C, Fernandez D, Kirou KA, Spiera RF, Crow MK, et al. Plasmacytoid dendritic cells promote systemic sclerosis with a key role for TLR8. Sci Transl Med. 2018 Jan 10;10(423):10.1126/scitranslmed.aam8458.
28. Davies CA, Herrick AL, Cordingley L, Freemont AJ, Jeziorska M. Expression of advanced glycation end products and their receptor in skin from patients with systemic sclerosis with and without calcinosis. Rheumatology (Oxford). 2009 Aug;48(8):876–82.
29. Pasch A, Farese S, Graber S, Wald J, Richtering W, Floege J, et al. Nanoparticle-based test measures overall propensity for calcification in serum. J Am Soc Nephrol. 2012;23(10):1744–52.
30. Lorenz G, Steubl D, Kemmner S, Pasch A, Koch-Sembdner W, Pham D, et al. Worsening calcification propensity precedes all-cause and cardiovascular mortality in haemodialyzed patients. Sci Rep. 2017;7(1):13366–8.
31. Keyzer CA, de Borst MH, van den Berg E, Jahnen-Dechent W, Arampatzis S, Farese S, et al. Calcification Propensity and Survival among Renal Transplant Recipients. J Am Soc Nephrol. 2016 Jan;27(1):239–48.
32. Pasch A, Block GA, Bachtler M, Smith ER, Jahnen-Dechent W, Arampatzis S, et al. Blood Calcification Propensity, Cardiovascular Events, and Survival in Patients Receiving Hemodialysis in the EVOLVE Trial. Clin J Am Soc Nephrol. 2017 Feb 7;12(2):315–22.
33. Dahdal S, Devetzis V, Chalikias G, Tziakas D, Chizzolini C, Ribi C, et al. Serum calcification propensity is independently associated with disease activity in systemic lupus erythematosus. PLoS One. 2018 Jan 24;13(1):e0188695.
34. Egashira R, Jacob J, Kokosi MA, Brun AL, Rice A, Nicholson AG, et al. Diffuse pulmonary ossification in fibrosing interstitial lung diseases: Prevalence and associations. Radiology. 2017;
35. Derlin T, Janssen T, Salamon J, Veldhoen S, Busch JD, Schon G, et al. Age-related differences in the activity of arterial mineral deposition and regional bone metabolism: a 18F-sodium fluoride positron emission tomography study. Osteoporos Int. 2015 Jan;26(1):199–207.
36. Rominger A, Saam T, Wolpers S, Cyran CC, Schmidt M, Foerster S, et al. 18F-FDG PET/CT identifies patients at risk for future vascular events in an otherwise asymptomatic cohort with neoplastic disease. J Nucl Med. 2009;50(10):1611–20.
37. de Boer SA, Hovinga-de Boer MC, Heerspink HJ, Lefrandt JD, van Roon AM, Lutgers HL, et al. Arterial Stiffness Is Positively Associated With 18F-fluorodeoxyglucose Positron Emission Tomography-Assessed Subclinical Vascular Inflammation in People With Early Type 2 Diabetes. Diabetes Care. 2016 Aug;39(8):1440–7.
38. Pruijm M, Lu Y, Megdiche F, Piskunowicz M, Milani B, Stuber M, et al. Serum calcification propensity is associated with renal tissue oxygenation and resistive index in patients with arterial hypertension or chronic kidney disease. J Hypertens.

2017;35(10):2044–52.
39. Clements P, Lachenbruch P, Siebold J, White B, Weiner S, Martin R, et al. Inter and intraobserver variability of total skin thickness score (modified Rodnan TSS) in systemic sclerosis. J Rheumatol. 1995 Jul;22(7):1281–5.
40. van Roon AM, Smit AJ, van Roon AM, Bootsma H, Mulder DJ. Digital ischaemia during cooling is independently related to nailfold capillaroscopic pattern in patients with Raynaud's phenomenon. Rheumatology (Oxford). 2016 Jun;55(6):1083–90.
41. Hofstee HM, Serne EH, Roberts C, Hesselstrand R, Scheja A, Moore TL, et al. A multicentre study on the reliability of qualitative and quantitative nail-fold videocapillaroscopy assessment. Rheumatology (Oxford). 2012 Apr;51(4):749–55.
42. Cutolo M, Pizzorni C, Tuccio M, Burroni A, Craviotto C, Basso M, et al. Nailfold videocapillaroscopic patterns and serum autoantibodies in systemic sclerosis. Rheumatology (Oxford). 2004 Jun;43(6):719–26.
43. Sulli A, Secchi ME, Pizzorni C, Cutolo M. Scoring the nailfold microvascular changes during the capillaroscopic analysis in systemic sclerosis patients. Ann Rheum Dis. 2008 Jun;67(6):885–7.
44. Sebastiani M, Manfredi A, Vukatana G, Moscatelli S, Riato L, Bocci M, et al. Predictive role of capillaroscopic skin ulcer risk index in systemic sclerosis: a multicentre validation study. Ann Rheum Dis. 2012 Jan;71(1):67–70.
45. Smith V, De Keyser F, Pizzorni C, Van Praet JT, Decuman S, Sulli A, et al. Nailfold capillaroscopy for day-to-day clinical use: construction of a simple scoring modality as a clinical prognostic index for digital trophic lesions. Ann Rheum Dis. 2011 Jan;70(1):180–3.
46. Warrick JH, Bhalla M, Schabel SI, Silver RM. High resolution computed tomography in early scleroderma lung disease. J Rheumatol. 1991;
47. Meerwaldt R, Graaff R, Oomen PH, Links TP, Jager JJ, Alderson NL, et al. Simple non-invasive assessment of advanced glycation endproduct accumulation. Diabetologia. 2004 Jul;47(7):1324–30.
48. Meerwaldt R, Links T, Graaff R, Thorpe SR, Baynes JW, Hartog J, et al. Simple noninvasive measurement of skin autofluorescence. Ann N Y Acad Sci. 2005 Jun;1043:290–8.
49. Honeybourne D, Babb J, Bowie P, Brewin A, Fraise A, Garrard C, et al. British Thoracic Society guidelines on diagnostic flexible bronchoscopy. Thorax. 2001.
50. Tanino M, Betsuyaku T, Takeyabu K, Tanino Y, Yamaguchi E, Miyamoto K, et al. Increased levels of interleukin-8 in BAL fluid from smokers susceptible to pulmonary emphysema. Thorax. 2002;
51. In't Veen JCCM, De Gouw HWFM, Smits HH, Sont JK, Hiemstra PS, Sterk PJ, et al. Repeatability of cellular and soluble markers of inflammation in induced sputum from patients with asthma. Eur Respir J. 1996;
52. Lapperre TS, Snoeck-Stroband JB, Gosman MME, Stolk J, Sont JK, Jansen DF, et al. Dissociation of lung function and airway inflammation in chronic obstructive pulmonary disease. Am J Respir Crit Care Med. 2004;
53. Emad A, Emad Y. Levels of cytokine in bronchoalveolar lavage (BAL) fluid in patients with pulmonary fibrosis due to sulfur mustard gas inhalation. J Interf Cytokine Res. 2007;
54. Wang Q, Van Timmeren MM, Petersen AH, Yuan J, Moser J, Brouwer E, et al. Age-determined severity of anti-myeloperoxidase autoantibody-mediated glomerulonephritis in mice. Nephrol Dial Transplant. 2017;
55. Elhai M, Hoffmann-Vold AM, Avouac J, Pezet S, Cauvet A, Leblond A, et al. Performance of Candidate Serum Biomarkers for Systemic Sclerosis–Associated Interstitial Lung Disease. Arthritis Rheumatol. 2019;
56. Valentini G, Riccardi A, Vettori S, Irace R, Iudici M, Tolone S, et al. CXCL4 in undifferentiated connective tissue disease at risk for systemic sclerosis (SSc) (previously referred to as very early SSc). Clin Exp Med. 2017;
57. Benyamine A, Heim X, Resseguier N, Bertin D, Gomez C, Ebbo M, et al. Elevated serum Krebs von den Lungen-6 in systemic sclerosis: a marker of lung fibrosis and severity of the disease. Rheumatol Int. 2018;
58. Lehtonen ST, Karvonen HM, Harju T, Sormunen R, Lappi-Blanco E, Hilli M, et al.

Stromal cells can be cultured and characterized from diagnostic bronchoalveolar fluid samples obtained from patients with various types of interstitial lung diseases. APMIS. 2014;
59. Mihara M, Moriya Y, Kishimoto T, Ohsugi Y. Interleukin-6 (IL-6) induces the proliferation of synovial fibroblastic cells in the presence of soluble Il-6 receptor. Rheumatology. 1995;
60. Wilkinson IB, Fuchs SA, Jansen IM, Spratt JC, Murray GD, Cockcroft JR, et al. Reproducibility of pulse wave velocity and augmentation index measured by pulse wave analysis. J Hypertens. 1998 Dec;16(12 Pt 2):2079–84.
61. O'Rourke MF, Gallagher DE. Pulse wave analysis. J Hypertens Off J Int Soc Hypertens. 1996 Dec;14(5):S147-57.
62. Luders S, Friedrich S, Ohrndorf S, Glimm AM, Burmester GR, Riemekasten G, et al. Detection of severe digital vasculopathy in systemic sclerosis by colour Doppler sonography is associated with digital ulcers. Rheumatology (Oxford). 2017 Nov 1;56(11):1865–73.
63. Schmidt WA, Krause A, Schicke B, Wernicke D. Color Doppler ultrasonography of hand and finger arteries to differentiate primary from secondary forms of Raynaud's phenomenon. J Rheumatol. 2008 Aug;35(8):1591–8.
64. Schmidt WA, Wernicke D, Kiefer E, Gromnica-Ihle E. Colour duplex sonography of finger arteries in vasculitis and in systemic sclerosis. Ann Rheum Dis. 2006 Feb;65(2):265–7.
65. Boellaard R, Delgado-Bolton R, Oyen WJ, Giammarile F, Tatsch K, Eschner W, et al. FDG PET/CT: EANM procedure guidelines for tumour imaging: version 2.0. Eur J Nucl Med Mol Imaging. 2015 Feb;42(2):328–54.
66. Bolster MB, Ludwicka A, Sutherland SE, Strange C, Silver RM. Cytokine concentrations in bronchoalveolar lavage fluid of patients with systemic sclerosis. Arthritis Rheum. 1997;40(4):743-751.
