# Supplementary material for: High serum C-X-C motif chemokine ligand 10 (CXCL10) levels may be associated with new onset interstitial lung disease in patients with systemic sclerosis: evidence from observational, clinical, transcriptomic and in vitro studies
Source: eBioMedicine. 2023 Nov 22;98:104883. doi: 10.1016/j.ebiom.2023.104883 (PMC10708993; doi:10.1016/j.ebiom.2023.104883)
Supplement: Protocol appendix study 3 [file mmc4.pdf]

Assessing the role of interleukin-6 in local inflammatory and fibroproliferative changes in Systemic Sclerosis related Interstitial Lung Disease: a proof of concept study.

IL(D)-6-SSc study

(non-WMO study protocol)

Date of document: 11-12-2018
Version number: 1.0

INHOUDSOPGAVE

1. STUDY ORGANIZATION 3
2. PROTOCOL SIGNATURE SHEET 5
3. ABSTRACT 5
4. BACKGROUND 5
5. METHOD 6
  5.1 Description study design 6
  5.2 Design 7
  5.3 Population 7
  5.4 Recruitment and informed consent/objection 8
  5.5 Research Data Management Plan (RDMP) 9
  5.6 Management of biomaterials 11
  5.7 Burden, Risks & Benefits (Prospective studies only) 13
  5.8 Incidental findings 13
  5.9 Data analysis 13
  5.10 Participant information after the study 13
  5.11 Research revenue 13
6. REFERENCES 14
7. APPENDICES (if applicable) 14

1. STUDY ORGANIZATION

**Study title**	**Assessing the role of interleukin-6 in local inflammatory and fibroproliferative changes in Systemic Sclerosis related Interstitial Lung Disease: a proof of concept study.**
**Planned start date**	01-01-2019
**Estimated completion date**	01-01-2021
**Project coordinator**	DJ Mulder, MD, PhD, internist-vascular specialist, dept. Internal Medicine/Vascular Medicine, University Medical Center Groningen, University of Groningen, University of Groningen, Groningen, The Netherlands.
**Principal investigators UMCG**	DJ Mulder, MD, PhD, internist-vascular specialist, dept. Internal Medicine/Vascular Medicine, University Medical Center Groningen, University of Groningen, University of Groningen, Groningen, The Netherlands.<br><br>J Westra, PhD, Assistant Professor and Laboratory Manager, Dept. Rheumatology & Clinical Immunology, University Medical Center Groningen, University of Groningen, University of Groningen, Groningen, The Netherlands.
**Researcher(s) UMCG**	A Eman Abdulle, MD, PhD student, dept. Internal Medicine/Vascular Medicine, University Medical Center Groningen, University of Groningen, Groningen, The Netherlands.<br><br>M Bijl, MD, PhD, rheumatologist/clinical immunologist, dept. Internal Medicine/Rheumatology, Martini Hospital Groningen, University of Groningen, Groningen, The Netherlands.<br><br>RA Bos, MD, PhD, rheumatologist, Department of Rheumatology, Medisch Centrum Leeuwarden , The Netherlands.<br><br>CT Gan, MD, PhD, pulmonologist, dept. Pulmonology, University Medical Center Groningen, University of Groningen, Groningen, The Netherlands.<br><br>Prof JK Burgess, PhD, Professor Extracellular Matrix in Disease Pathogenesis, Rosalind Franklin Fellow, Department of Pathology and Medical Biology, Medical Biology Section, University Medical Center Groningen, University of Groningen, Groningen, The Netherlands.<br><br>Prof W Timens PhD, Head of Department of Pathology and Medical Biology, Medical Biology Section, University Medical Center Groningen, University of Groningen, Groningen, The Netherlands.<br><br>Corry-Anke Brandsma, PhD PhD, Department of Pathology and Medical Biology, Medical Biology Section, University Medical Center Groningen, University of Groningen, Groningen, The Netherlands.

**Corresponding researcher UMCG**	DJ Mulder, MD, PhD, internist-vascular specialist, dept. Internal Medicine/Vascular Medicine, University Medical Center Groningen, University of Groningen, University of Groningen, Groningen, The Netherlands.
**(Principal) investigator other centers**	NA
**Sponsor (in Dutch: verrichter/opdrachtgever)**	UMCG
**Financial support/ Subsidising party**	NA
**Collaboration with non-profit Laboratory / research sites (in- and outside UMCG)**	NA
**Collaboration with commercial parties / companies (in- and outside UMCG)**	NA
**Name bio- or databank and bankmanager**	J.J. Duker<br>Biobank Pathologie<br>Department of Pathology and Medical Biology, Medical Biology Section, University Medical Center Groningen, University of Groningen, Groningen, The Netherlands.<br>j.j.duker@umcg.nl☒ approved by the Board of Directors UMCG<br>☐ not approved by the Board of Directors UMCG
**Name previous study ('FAIR data') and (principal) investigator**	NA

2. PROTOCOL SIGNATURE SHEET

The undersigned (Principal) investigator and head of department UMCG confirm that the study and its procedures will comply with the present study protocol and the nWMO Kaderreglement UMCG. Without ethical approval the data/biomaterials will not be used for other (research) purposes (e.g. 'FAIR data').

Name	Signature	Date
**(Principal) investigator UMCG:**	DJ Mulder	11-12-2018
**Head of the department UMCG:**	ROB Gans	11-12-2018
**Other (if applicable)**		

Please also refer to the “Biobank Pathologie” protocol, v 1.2: 21-02-2018, attached to this document.

3. ABSTRACT

- **Background**
  Systemic sclerosis (SSc) is an autoimmune condition characterized by fibrosis of skin and internal organs. Interstitial lung disease (SSc-ILD) is a major problem in SSc. In early disease, interleukin-6 may play a crucial role and may offer a potential target for disease modifying interventions.

- **Main research question**
  To investigate the pathophysiological role of IL-6 in SSc-associated ILD

- **Design (including population, confounders/outcomes)**
  Cross-sectional and experimental

- **Expected results**
  With the current approach we aim to demonstrate a central role of Il-6 in early SSc-ILD. If our hypothesis proves to be valid, it will pave the way for a clinical interventional study in patients with early SSc-ILD.

4. BACKGROUND

- **Introduction and rationale**
  Systemic sclerosis (SSc) is an autoimmune condition characterized by fibrosis of skin and internal organs. In SSc, fibroblasts play a central role in the biology of the disease by interacting with endothelial cells and leukocytes in a complex biological network involving cytokines and adhesion molecules, resulting in excess deposition of extracellular matrix proteins and in tissue stiffening. Interstitial lung disease (SSc-ILD) is a major problem in SSc, since it accounts for about one-third of deaths and no disease modifying treatment is currently available. Recent reports have shown that early phases of SSc-ILD are associated with more pronounced inflammation, which may change to a more pro-fibrotic phenotype leading to irreversible fibrosis in later stages of disease.

Currently, only patients with extensive lung disease who have a poor prognosis and progressive fibrotic course qualify for treatment. First-line treatment options are limited to cyclophosphamide or Mycofenolate Mofetil (MMF). Due to adverse side effects and absence of studies in early SSc-ILD, these therapies are reserved for those patients with severe disease. We hypothesize that early treatment of the inflammatory phase in SSc-ILD would be favorable in preventing progression to irreversible lung fibrosis with interventions that targeted anti-inflammatory effects and had limited side effects.

Interleukin-6 (IL-6) has been shown to play an important role in SSc by regulating the function of immune and non-immune cells. IL-6 is known to induce TGFβ production and to enhance TGFβ-signaling in dermal and cardiac fibroblasts. Conversely, TGFβ regulates the expression of IL-6 by lung fibroblasts and airway smooth muscle cells. We have recently shown that dermal fibroblast are capable of producing extremely high levels of IL-6 locally after stimulation. Dermal fibroblasts from patients with SSc express increased levels of IL-6 and an increased serum IL-6 levels predict higher mortality risk, worse skin involvement and increased pulmonary decline in SSc. In congruence with fibroblasts, alveolar macrophages obtained from SSc-ILD patients also appear to produce IL-6 at extremely high levels after GM-CSF stimulation. Elevated serum levels of IL-6 are associated with early disease. Importantly, IL-6 is only predictive of ILD outcome in patients with early disease, where it is not reflective in more progressed stages. Recently, a profound impact of IL-6R blockade on the activated fibroblast phenotype was shown using explant dermal fibroblast cultures from SSc patients before and after treatment. These data underline the importance of IL-6 in the early inflammatory phases of SSc and could be a novel target for early treatment of SSc-ILD.

Research question

We hypothesize that IL-6 plays a major role in early phase of the development of interstitial lung disease (ILD) in patients with Systemic Sclerosis (SSc). Since CRP levels in SSc patients with limited disease are generally only slightly elevated, the role of IL-6 is thought to be a local one, acting within the target tissue of the lung. In order to establish this role, several objectives need to be addressed.

Objectives:

1. to assess upregulation of inflammatory markers, including IL-6, in histological samples from patients with SSc-ILD
2. to assess the pro-inflammatory signature of lung fibroblasts obtained from lung biopsies and their IL-6 production after stimulation and whether this signature is influenced favorably by blockage of the IL-6 pathway.

5. METHOD

5.1 Description study design

Design:

This is a proof of concept study to establish the role of IL-6 in SSc-ILD, for which lung tissue have been obtained from waste material obtained from clinical practice including lung-explants following lung transplantation, obduction, and diagnostic procedures (VATS of open lung biopsies) for SSc-ILD. In vitro experiments on fibroblasts isolated from lung biopsies obtained for clinical practice.

5.2 Design

5.2.1 Mono- or multicenter study	Mono-center study<br>yes	Multicenter study<br>no
5.2.2 Retrospective study (available data/ biomaterials only) or prospective study (data/ biomaterials from [some] participants will be collected in the future).	Retrospective study<br>yes	Prospective study<br>no
5.2.3 Cross-sectional or follow-up study	Cross-sectional study<br>yes	Follow-up study<br>no
5.2.4 Quantitative or qualitative study (click both if mixed-method)	Quantitative study<br>yes	Qualitative study<br>Yes

5.2.4 Pilot study: yes

Methods and materials

Histopathology study:
- IHC staining of lung biopsy material using H&E, myofibroblast (α-Smooth muscle actin, αSMA) endothelial cells (CD31), macrophages (CD68), cytokines and chemokines including IL-6. Colocalization of IL-6 with inflammation and fibrosis (Massons Trichrome and collagen Ia1 stain). Interferon-I upregulation (myxovirus resistance protein (MxA)).

In *vitro/ex vivo* studies:
Fibroblast will be sourced from BAL and biopsies. This has previously been shown to be feasible in material from patients with various types of interstitial lung diseases [Lehtonen 2014].

Measures:
Primary human lung fibroblasts will be stimulated with TGF-β to study
- Profibrotic signature: Connective Tissue Growth Factor, Col1, αSMA by RNA expression and protein production.
- IL-6 production
- Migratory capacity and contractility activity by Using wound healing (scratch assays) and transwell expt for migration/proliferation. Collagen gel contraction assays to assess fibroblast contractility.
- RNA profiling before and after stimulation with TGF
- Culture of lung fibroblast obtained from bronchus brushes and bronchoalveolar lavage (BAL) from patients with SSc-ILD and effect of systemic treatment on profibrotic signature, IL-6 production and RNA profiling.

5.3 Population

5.3.1 Inclusion and exclusion criteria

- Inclusion criteria
    - Diagnosis of Systemic Sclerosis, as determined by 2013 ACR/EULAR criteria, as established by the treating physician.
    - A formal diagnosis of SSc-ILD based of typical lung function or HRCT abnormalities as established by the treating physician.

- Exclusion criteria
    - ILD as a result of other connective tissue disease, idiopathic pulmonary fibrosis
    - Other lung diseases associated with inflammation or fibrosis, including COPD, asthma, cystic fibrosis, lung cancer.

<u>5.3.2 Number of participants</u>
- Target total number of participants: 25 specimen
- Target number of UMCG participants: 25

<u>5.3.3 Study subjects</u>

• Healthy volunteers	no
• Patients	yes

<u>5.3.4 Subject classification</u>

• Participants ≥ 16 years	yes
• Children between 12 and 16 years *(written informed consent will be obtained from child and both parents - if both have authority, or guardian [or parents/guardian only if incapacitated child])*	no
• Children < 12 years *(written informed consent will be obtained from both parents - if both have authority, or guardian)*	no

<u>5.3.5 Incapacitated adults</u>

Participants are incapacitated/ decisionally incompetent adults *(written informed consent will be obtained from legal representative)*	no

5.4 Recruitment and informed consent/objection

5.4.1 <u>Retrospective study (tick all that apply)</u>

☐ Not applicable <u>(see section 5.2.2)</u>

☒ Data will be copied from (electronic) patient records (e.g. 'nieuw EPD' UMCG)

- A list of specimen potentially suitable for the study will be supplied to the researcher. In the patient record, data will be identified (if available) which are needed for interpretation of the study results, and copied to a case report form, these include:
    - Age specimen was obtained, sex, race, body weight, length
    - Clinical characteristics of SSc, including SSc subtype (diffuse cutaneous or limited cutaneous), EULAR/ACR 2013 criteria (proximal skin thickening, skin thickening of fingers, finger tip lesions, telangiectasia, nail-fold capillaroscopy, presence of PAH or ILD, Raynaud's phenomenon, SSc-related antibodies), presence of calcinosis cutis, gastrointestinal dysmotility, cardiac involvement, renal involvement
    - Characteristics of ILD, i.e. HRCT characteristics, lung function (FVC and DLCO uncorrected), if available 6 minute walking test
    - Routine lab values including CRP,BSE,blood count, creatinine, liverfunction tests
    - Reason for biopsy
    - Outcome (remission, improvement, stabilization, worsening on therapy, death, cause of death, lung transplantation)
    - Current therapy, history of DMARD therapy
- Total number of patients who will not be asked informed consent for screening: 0

- Total number of UMCG patients who will not be asked informed consent for screening: 25

☒ Data/biomaterials will be obtained from an already existing internal or external (UMCG) bio- or databank (see Section 1. Study organization).
Biobank Pathologie ☐ Data/biomaterials will be obtained from a previous study ('FAIR data' - internal/external; see Section 1. Study organization).
<text>

5.4.2 Prospective study
☒ Not applicable (see section 5.2.2)

5.4.3 Objection (Registry)

in case one or more participants will not be asked informed consent, the objection registry will be checked for these participants and the data from those who objected will be excluded from the analyses.	yes

5.4.4 Informed consent (IC): access to identifiable participant data

in case one or more study team members will have access to direct/indirect identifiable participant data, informed consent will be/has been obtained for this access.	NA

5.4.5 IC: Collaboration with commercial parties

In case of collaboration with commercial/profit organizations, informed consent will be/has been obtained for this type of collaboration	NA

5.4.6 IC: Linking with other registries

In case the data will be linked with other registries, informed consent will be/has been obtained for this linkage(s)	NA

5.4.7 IC: Incidental findings

In case there is a risk of incidental findings, informed consent will be/has been obtained to return findings to the participant	NA

5.4.8 IC: FAIR Data

In case data collected for the present study will be shared for future studies, informed consent will be obtained for this	NA

5.4.9 IC: other aspects
NA

5.4.10 Withdrawal

• Can participants withdraw informed consent before publication and will all data/ biomaterials of that participant be destroyed	NA
• Does the participant information letter contain information on how to withdraw	NA

No IC will be obtained, UMCG objection registry will be checked.

5.5 Research Data Management Plan (RDMP)

In this study the data will be collected, processed, and archived in accordance with the General Data Protection Regulation (GDPR) and the FAIR (Findable, Accessible, Interoperable, Reusable) principles

under the responsibility of the Principal Investigator. A research data management plan (RDMP) will be drawn up to describe the further operational details and procedures.

X the RDMP section below is completed
☐ a separate RDMP document will be attached to this protocol

5.5.1 Data collection

- Only essential baseline characteristics and data required to answer the research question(s) will be collected. yes
- Tooling (eg. software and procedures) used for collecting, processing, analysing, and storing data will be compliant with the UMCG policy and Standard Operating Procedures in the UMCG Research Toolbox. yes

5.5.2 Anonymization and pseudonymization

- Data will be anonymised during data collection (i.e. data cannot be linked back to the participant) Yes
- Data will be pseudonymized by use of a code list during data collection. yes
- Indirect and direct identifiable information collected will be minimized and only collected for the purpose of this study yes
- Direct identifiable information will be stored separately from pseudonymized data in an electronic file yes

5.5.3 Data access (during the study)

- Direct identifiable information can only be accessed by the Principal Investigator and study delegates after authorization by the Principal Investigator. yes
- Pseudonymized/anonymized data can only be accessed by the Principal Investigator and study delegates after authorization by the Principal Investigator. yes
- Data roles, responsibilities, access and authorization - during the study and after study completion - will be managed and documented (e.g. in the RDMP, on study delegation log). yes

5.5.4 Data sharing (during and after study completion)

In case data (and biomaterials) will leave the UMCG, will you contact the loket Contract Research to arrange the proper contracts? (Loket_Contract_Research@umcg.nl) yes

5.5.5 Data storage (during and after study completion)

- Digital data will be archived on the UMCG network complying with strict UMCG security and back-up policy. yes
- Paper source data and study files will be archived within the UMCG facilities. yes

- Source data, study files and digital data will be stored 15 years after the study is completed. yes

5.5.6 Data re-use and access after completion of the present study ('FAIR data') NA ☒

5.6 Management of biomaterials

Will biomaterials be collected, processed, analyzed and/or stored for the purpose of this study No **skip section 5.6**

<text>

5.6.1 Retrospective study (see sections 1, 5.2.2, and 5.4.1) NA ☒

If biomaterials will be used from a secondary/further use biobank that has not been approved by the Board of Directors of the UMCG, how will be prohibited that biomaterials necessary for future diagnostic/treatment purposes will be used in the present study.

<text>

5.6.2 Biomaterials collection

- Only biomaterials required to answer the research question(s) will be collected yes/no
- What biomaterials will be collected
  <text>
- How will the biomaterials be collected and processed
  <text>

5.6.3 Pseudonymization and access to biomaterials

- Does the storage unit of the biomaterials comprise information that the participant (in)directly identifies, other than the participant's number and / or the sample number. yes/no
  <if yes, explain>
- Biomaterials can only be accessed by the Principal Investigator and study delegates after authorization by the Principal Investigator yes/no
  <if no, explain>

5.6.4 Sharing of biomaterials (during and after study completion)

In case biomaterials (and data) will leave the UMCG, will you contact the loket Contract Research to arrange the proper contracts? (Loket_Contract_Research@umcg.nl) NA/yes/no

<if no, explain>

5.6.5 Biomaterials storage (during and after study completion)

- Where and how will the biomaterials be stored
  <text>
- Biomaterials will be stored 15 years after the study is completed yes/no
  <If no, explain and give number of years>
- What will be done with the remaining biomaterials after study completion (eg. destroyed, returned to biobank/previous study, stored)
  <text>

5.6.6 Biomaterials re-use and access after completion of the present study ('FAIR data') NA ☒ **skip section 5.6.6**

- Biomaterials will become available and shared for re-use and participants will be asked informed consent for this ('FAIR data') yes/no
  <if no, specify and explain>
- Biomaterials will be made findable by including the description of the study (and type of biomaterials in the UMCG FAIR data catalogue and other discipline specific catalogue(s). yes/no
  <if no, explain>
- Review procedure, conditions and agreements for re-use of biomaterials and access to biomaterials by other researchers will be drawn up. yes/no
  <if no, explain>

5.7 Burden, Risks & Benefits (Prospective studies only)

- If participants are patients: Can be deviated from the standard care / diagnostic procedures (e.g. can medical treatment be postponed or limited) — NA
  <text>
- Burden
  <text>

	Yes, minimal risk/burden	Yes, more than minimal risk/burden	No
• Will the participants risk any injuries and/or other discomfort when they participate in the proposed study	☐	☐	☐

<text>

- Participant benefits/reward/incentives:
  <text>

5.8 Incidental findings

	yes, minimal risk	yes, ≥ substantial risk	No
• Is there a risk of incidental findings?	☐	☐	☒

<text>

If yes,
- Procedure to assess if a finding should be returned to the participant, or not
  <text>
- Procedure to inform the participant
  <text>

5.9 Data analysis

- Power analysis
  NA
- Statistical analysis
  Statistical analysis will be carried out using IBM SPSS Statistics version 23 and will be largely descriptional. If applicable, effect sizes will be calculated which will help calculating sample sizes for future studies. Non-parametric statistical test will be performed due to small number. Differences between groups were tested with Fisher's exact test. Spearman's rho will be applied for association. Values of $p<0.05$ are considered statistically significant. Data will be described as median (IQR) or number (percentage).

5.10 Participant information after the study

Will participants be informed about the study results — no

5.11 Research revenue

In case the study will result in revenues (e.g. as a result of the use of data/biomaterials or successful licensing of intellectual property or manufactured products), will you contact the loket Contract Research to arrange the proper contracts? — NA

<if no, explain>

Describe what will be done with the revenues.

<text>

6. REFERENCES

Available upon request.

7. APPENDICES (if applicable)

Please also refer to the "Biobank Pathologie" protocol, v 1.2: 21-02-2018, attached to this document.
