# Supplementary material for: High serum C-X-C motif chemokine ligand 10 (CXCL10) levels may be associated with new onset interstitial lung disease in patients with systemic sclerosis: evidence from observational, clinical, transcriptomic and in vitro studies
Source: eBioMedicine. 2023 Nov 22;98:104883. doi: 10.1016/j.ebiom.2023.104883 (PMC10708993; doi:10.1016/j.ebiom.2023.104883)
Supplement: RNA Extraction to qPCR protocols [file mmc6.docx]

**RNA Extraction to qPCR protocols**

**RNA isolation**

*Start with putting on the centrifuge at 4C.

- Remove the medium from cells to the ELISA eppendorf cups and add 1 ml TRIzol reagent to each well
- Incubate 5 minutes at 15-30°C and put each sample in a separate RNA-free eppendorf tube
- Perform directly RNA isolation or store samples at -80°C. When applicable, take samples from -80°C freezer and completely thaw samples
- Add 0,2 ml chloroform to all tubes and vortex/shake samples **at least for 15 seconds**
- Incubate for 2-3 minutes at RT
- After incubation, centrifuge tubes for 15 minutes at 4°C (10000 rpm)
- Collect the aqueous upper layer in new RNA-free eppendorf cups (around 430 µL)
- Add 0,5 ml isopropanol to each sample and vortex
- Incubate 10 minutes at RT
- Centrifuge for 10 minutes at 4°C (10000 rpm)
- Decant supernatant and add 1 ml ice-cold 75% ethanol and vortex briefly
- Centrifuge for 5 minutes at 4°C (10000 rpm)
- Decant ethanol and dry pellet during 1h by put the tubes upside down
- After drying of the pellets perform DNAse treatment

**DNAse treatment**

- Prepare for n+1 samples a mixture of 2,5 µl DNAse buffer + 22,5 µl RNAse-free water+1 µl DNAse (take the DNAse out of the freezer just before adding it to the mixture - or else enzyme activity will be reduced).
- Add 26 µl mixture to each sample and resuspend the pellet ( by gently tapping the tubes)
- Incubate for 30 minutes in the incubator at 37°C
- Add 5 µl inactivation reagent to each sample, resuspend and incubate for 2 minutes at RT
- Centrifuge briefly
- RNA is present in the supernatant and cDNA can be synthesized according to protocol ‘cDNA synthesis’
- Measure RNA concentration with nanodrop (on ice)
- For all samples with RNA concentration ≤ 100 ng/μl use all (= 25 μl)* RNA and put in small PCR tube
- Samples that exceed 100 ng/μl:
  - RNA needed (μl) = 2500 / (RNA concentration in sample)
  - Put RNA in small PCR tube and supplement with water till 25 μl is reached

*Usually, it is not 25µL but 23µL max. Therefore, add water accordingly til 25µL total volume.

**cDNA Synthesis**

- Heat the samples for 10 minutes at 65°C in water and after that put the samples on ice
- Prepare for n+1 samples a RT mix:
  - 25 mM dNTP’s
  - M-MLV Reverse Transcriptase enzyme*
  - 5x First Strand buffer
  - 0,1 M DTT
  - 7,5 µM oligo (dt)24
  - DEPC water
- Add 15 µl mix to each sample
- Spin down shortly the PCR tubes in the spinner at the PCR room
- Put PCR tubes in PCR machine for 1h at 41°C, 5 minutes at 95°C and ∞ at 4°C
- Store cDNA at -20°C

​*Addition of enzymes should be always the last step of making a mixture

**qPCR workflow**

- Apply 1 µl cDNA in duplo into a 384-wells plate
- Centrifuge plate under a vacuum using the DNA speedvac at a medium drying range for 10 minutes (ERIBA)
- Add desired primer per sample: 0,5 µl primer/probe + 7 µl water + 2.5 µl taqman premix
- Add 10 µl to each well
- Seal plate (Optical Seal by Applied Biosystems) and centrifuge shortly (no vacuum)
- Put plate in the Quanstudio 7 Flex (ERIBA)
- Go to Quanstudio Real-Time PCR software
- Choose new experiment
- Setup -->  experiment properties --> Scan the plate barcode, choose: Quantstudio Flex System, 384-well, comparative Ct (ΔΔCt), TaqMan Reagents, standard
- Setup --> run method --> Volume per well is 10 μl, delete first step, second step is 5 minutes at 95˚C, third step is 15 seconds at 95˚C and last step is 30 seconds at 60˚C. Number of cycles is 40.
- Choose save --> save as and save experiment in folder qPCR-documents

        Go to run --> start run and click on the machine number
