# Supplementary material for: High serum C-X-C motif chemokine ligand 10 (CXCL10) levels may be associated with new onset interstitial lung disease in patients with systemic sclerosis: evidence from observational, clinical, transcriptomic and in vitro studies
Source: eBioMedicine. 2023 Nov 22;98:104883. doi: 10.1016/j.ebiom.2023.104883 (PMC10708993; doi:10.1016/j.ebiom.2023.104883)
Supplement: COA CXCL10 DY266_P294754 [file mmc7.pdf]

R&D SYSTEMS
a bio-techne brand

Human CXCL10/IP-10 DuoSet ELISA, 15 Plate

Catalog Number: DY266
Expiration: 21 Jun 2026
Lot: P294754

Certificate of Analysis

SPECIFICATIONS

REAGENT	PART NUMBER	# OF VIALS	AMOUNT PER VIAL	WORKING CONCENTRATION	LOT #
Capture	840420	3	120 µg	2.00 µg/mL	ADN0621041
Detection	840421	3	1.20 µg	20.0 ng/mL	AAP1521041
Standard	840422	3	55.0 ng	31.2-2000 pg/mL	1513403
Streptavidin-HRP	893975	3	N/A	40-fold dilution	P289330

PREPARATION & STORAGE

Store unopened kit at 2-8 °C. Do not use past kit expiration date.

REAGENT	PREPARATION	STORAGE OF OPENED/RECONSTITUTED MATERIAL
Capture	Reconstitute with 0.5 mL of PBS	Store at 2-8 °C for up to 8 weeks or aliquot and store at -20 °C to -70 °C in a manual defrost freezer for up to 12 weeks.*
Detection	Reconstitute with 1.0 mL of Reagent Diluent	
Standard	Reconstitute with 0.5 mL of Reagent Diluent	Store reconstituted standard at 2-8 °C for up to 8 weeks or aliquot and store at -70 °C for up to 12 weeks.*
Streptavidin-HRP	Dilute with Reagent Diluent	Store undiluted at 2-8 °C for up to 12 weeks. DO NOT FREEZE.*

*Provided this is within the expiration date of the kit.

It is hereby certified that the above product has been tested for proper performance and function under our established Quality Control Testing criteria.
It is authorized by our Quality Assurance program to be released for sale.

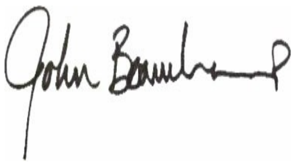

John Beauchamp
Manager, Quality Assurance/Regulatory Affairs
This C of A was updated on 7/30/2021, Rev. 0

For Research Use or Further Manufacturing Purposes Only

bio-techne®
Global bio-techne.com info@bio-techne.com techsupport@bio-techne.com TEL +1 612 379 2956
USA TEL 800 343 7475 Canada TEL 855 668 8722 China TEL +86 (21) 52380373
Europe | Middle East | Africa TEL +44 (0)1235 529449
